# Convenient Method of Synthesizing Aryloxyalkyl Esters from Phenolic Esters Using Halogenated Alcohols

**DOI:** 10.3390/molecules23071715

**Published:** 2018-07-13

**Authors:** Xueyang Jiang, Junting Zhou, Yue Zhou, Haopeng Sun, Jian Xu, Feng Feng, Wei Qu

**Affiliations:** 1 Department of Natural Medicinal Chemistry, China Pharmaceutical University, Nanjing 210009, China; ajyang@stu.cpu.edu.cn (X.J.); zhoujunting0920@163.com (J.Z.); xujian201009@126.com (J.X.); 2 Department of Pharmacy Administration & Clinical Pharmacy, Peking University, Beijing 100191, China; yuezhouzhy@163.com; 3 Department of Medicinal Chemistry, China Pharmaceutical University, Nanjing 210009, China; sunhaopeng@163.com; 4 Key Laboratory of Biomedical Functional Materials, China Pharmaceutical University, Nanjing 210009, China; 5 Food and Drug Research Institute, Jiangsu Food & Pharmaceutical Science College, Huaian 223003, China

**Keywords:** aryloxyalkyl esters, phenolic esters, acyl transfer

## Abstract

A facile one-pot synthetic method of building aryloxyalkyl esters was developed using various types of phenolic esters with halogenated alcohols. The ready availability of both starting materials, coupled with the required simple experimental technique, enables the current synthetic method of producing aryloxyalkyl esters in a fast and efficient way. It is noteworthy that acyl transfer was demonstrated in this reaction.

## 1. Introduction

**Scheme 1:**
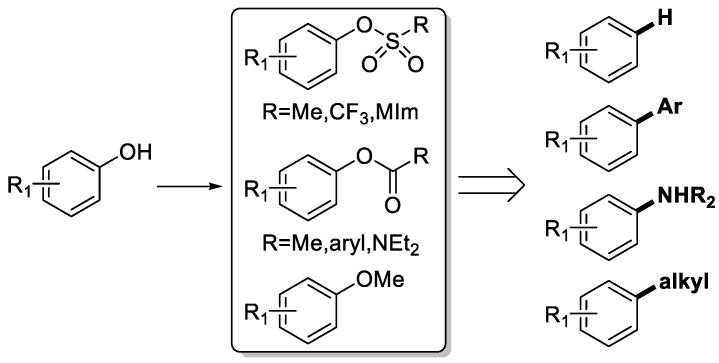
The structures of C-O electrophiles and their subsequent conformation of C-C bonds, C-N bonds, and C-H bonds.

Phenols are typically protected by various groups to enhance their leaving ability in cross-coupling reactions [1,2,3]. Thus, phenols have usually been converted to their corresponding aryl C-O electrophiles, such as fluoroalkylsulfonates [4,5], sulfamates [6,7,8], sulfonates [9,10,11,12], carbonates [13,14,15], carbamates [16,17], ethers [18], phosphoramides, phosphonium salts, phosphates, and pivalates [19,20,21,22]. Besides the preferable availability of phenol derivatives, this method also proves to be cost-effective, as well as an efficient reaction route to form C-C bonds, C-N bonds, and C-H bonds (Scheme 1) [23,24,25,26,27,28]. Phenolic esters, a type of aryl C-O electrophiles, are considered as notable functional groups or protecting groups in organic synthesis, and can be found in various bioactive natural products, agrochemicals, pharmaceuticals, and functional polymers [29,30,31].

**Scheme 2:**
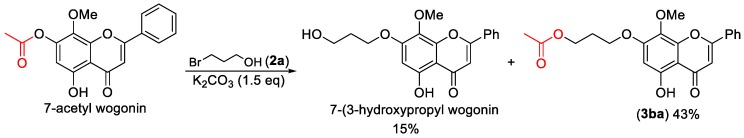
The new method to synthesize aryloxyalkyl ester.

To further the interest in the modification of flavonoids [32], we tried to synthesize 7-(3-hydroxypropoxy) wogonin by modifying the 7-acetate of wogonin with 3-bromoethanol. Unfortunately, the desired product (7-hydroxypropoxy wogonite) was rarely obtained. Aryloxyalkyl ester (**3ba**), however, was serendipitously obtained through the reaction displayed in Scheme 2. Previous study has indicated that aryloxyalkyl esters were important moieties in organic syntheses, primarily due to ubiquitous applications in pharmaceuticals, electrophosphorescent materials, plasticizers, and luminescent materials [33,34,35,36]. The preparation of aryloxyalkyl esters was mainly though a two-step process: Introduction of an aliphatic chain, followed by esterification with carboxylic acids or anhydrides [37,38]. Undoubtedly, the efficiency of these protocols was restricted from the view of step economy for the synthesis of a variety of substituents. Herein, we disclose a convenient synthesis of aryloxyalkyl esters from phenolic esters with halogenated alcohol.

## 2. Results and Discussion

First, we investigated the effects of reaction parameters in this new aryloxyalkyl esterification reaction (Table 1). Phenyl acetate (**1a**) was chosen as a model substrate and subjected to a preliminary condensation condition by using 3-bromoethanol (**2a**) (1.05 equiv) and K_2_CO_3_ (1.5 equiv) in commercial grade acetone under reflux for 6 h. Gratifyingly, the phenolic ester extension reaction proceeded smoothly, with concomitant cleavage of the ester bond and construction of the ether bond, delivering 3-phenoxypropyl acetate (**3aa**) in 59% isolated yield (entry 1). Changing the solvent from acetone to tetrahydrofuran or acetonitrile resulted in inferior results (entries 2 and 3). However, when *N*,*N*-dimethylformamide (DMF) was used as the solvent, **3aa** was obtained in comparably good conversions (entry 4). By increasing the amount of potassium carbonate, further enhancive conversion was observed, which have been used to establish the optimal conditions (entry 5). No reaction was observed without a base (entry 6), whereas a weak base depressed the conversion (entry 7), indicating that the base could break the ester bond. Two other bases, i.e., Cs_2_CO_3_ and NaH, afforded the products (entries 8 and 9) with similar yields to entry 5. When NaOH was used as the base, only a trace amount of the target product, **3aa**, was detected by high performance liquid chromatography (HPLC), instead of generating a large amount of 3-phenoxypropan-1-ol. (entry 10).

**Scheme 3:**
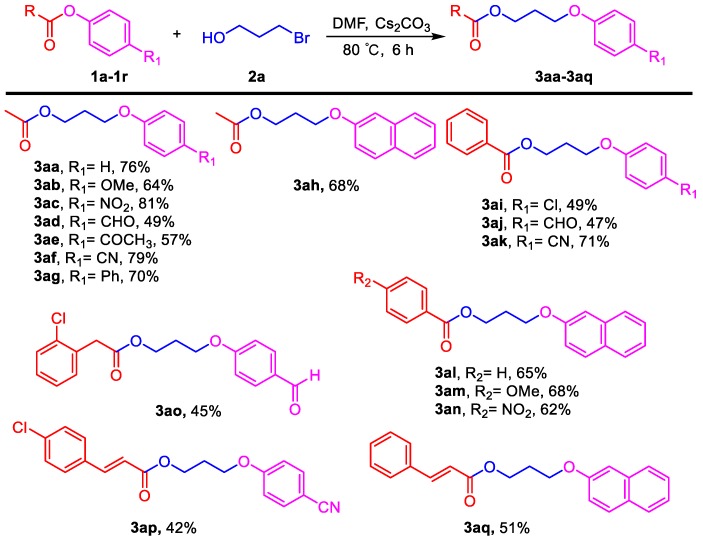
Scope of the reaction of phenol esters.

Next, we investigated the application range of this reaction by using a variety of phenol esters and bromoalkyl alcohols under the optimized conditions: 0.5 mmol of **1** and 0.525 mmol of **2** in DMF at 80 °C for 6 h under an Ar atmosphere (Scheme 3). Both electron-rich and electron-withdrawing substituents produced the desired aryloxyalkyl esters in good yields (**3ab** and **3ac**). It is noteworthy that substrates bearing functional groups (e.g., aldehyde, ketone, nitrile, or halogen) also showed good performance in this reaction (**3ad**–**ai**). To further extend the utility of this reaction, we then put our effort to the types of acid esters from the common acetates. It was observed that various substituted phenyl benzoates reacted well, leading to the formation of the corresponding aryloxyalkyl esters (**3aj**–**an**) in yields of 47–71%. Notably, chlorophenylacetate moiety was tolerated, providing the target product, **3ao**, in 45% yield. Delightfully, we found that even cinnamate derivatives under the reaction conditions could give the corresponding products (**3ap** and **3aq**) in moderate to good yields (with increased base loading). Changing the starting material from phenyl ester to alkyl ester, however, resulted in a large amount of alkyl ester remaining and no target product, which might be because the ester bond of alkyl ester was difficult to be broken (see Scheme S1 in Supplementary Materials).

**Scheme 4:**
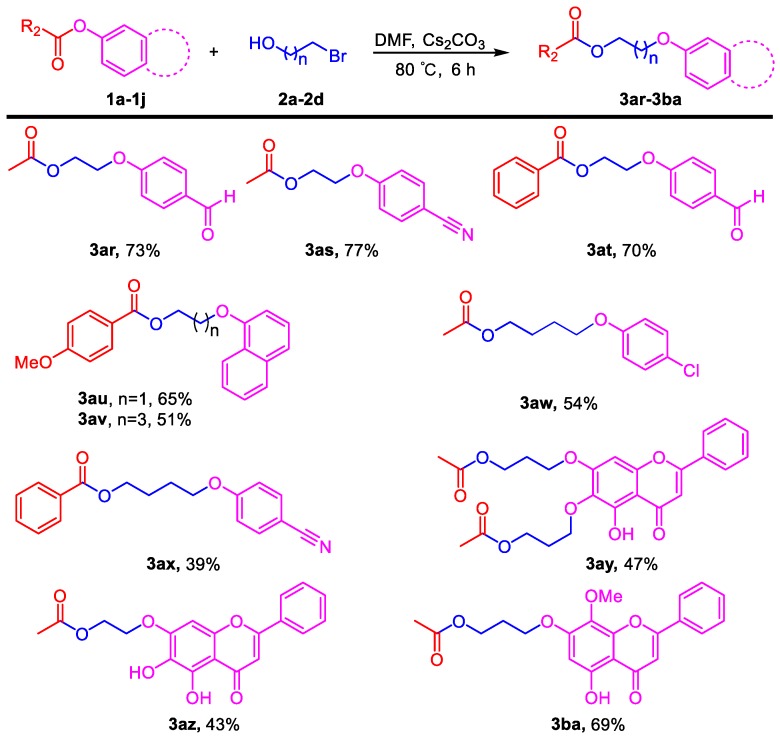
Scope of the reaction of halogenated alcohols and application to the synthesis of flavonoid derivatives.

Furthermore, the diversity of halohydrins was also studied (Scheme 4). 2-Bromoethanol (**2b**), 4-bromobutanol (**2c**), and 5-bromopentanol (**2d**) performed similarly to produce the corresponding products, **3ar**–**ax**, in moderate to high yields. For the synthesis of compound **3ar**, the usage of 2-bromoethanol, 2-chloroethanol, or 2-iodoethanol contributed similar yields, wherein the use of 2-iodoethanol slightly increased the yield of **3ar**. The longer alkyl chain resulted in lower yield. The scope of the present investigation was extended to flavone derivatives. Baicalein and wogonin were applicable under our reaction conditions, providing the target products (**3ay**, **3az** and **3ba**) in moderate yields.

**Scheme 5:**
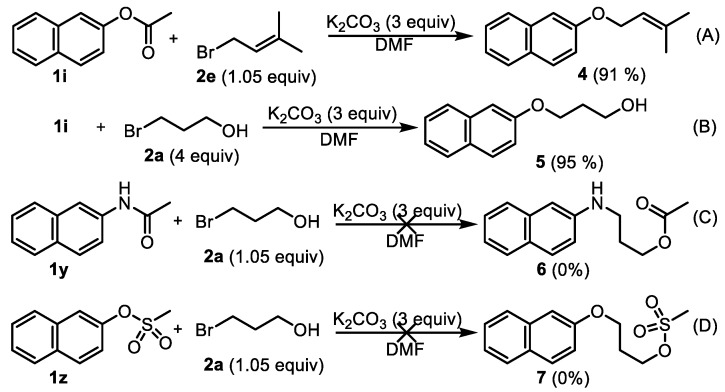
Control experiments to investigate the mechanism.

A series of control experiments were carried out to elucidate the reaction mechanism (Scheme 5). Firstly, as 3-methyl-2-butenyl bromide (**2e**) was used to substitute 3-bromoethanol (**2a**), the product, 1-isopentenyl naphthol (**4**)**,** was obtained instead of the target product (Scheme 5A). Secondly, when the amount of 3-bromopropanol (**2a**) was increased from 1.05 equivalents to 4 equivalents, 3-(2-naphthoxy)propan-1-ol (**5**) was obtained, which suggested that the generated acetyl reactive intermediate might not be selective in reacting with the alcoholic hydroxyl group (Scheme 5B). No reaction was observed when no potassium carbonate was used, indicating that the phenolic ester bond was firstly broken by potassium carbonate. Further, sulfonates and aromatic amides were found to be incompatible with the reaction conditions and resulted in nothing even under refluxing conditions, suggesting that potassium carbonate could not attack the carbonyl group of the acylamine bonds because the polarization of the carbonyl group is weaker compared with the ester carbonyl group (Scheme 5C,D). However, when the strong base, NaH, was used, the deprotonation of **2a** formed an oxygen negative intermediate, which would attack the carbonyl group at a high temperature.

**Scheme 6:**
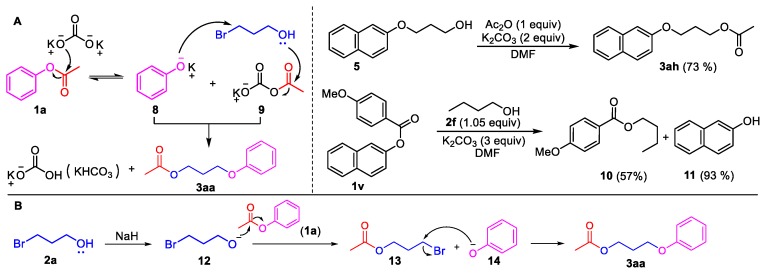
Proposed mechanism for the synthesis of aryloxyalkyl ester derivatives in the presence of potassium carbonate (**A**), or in the presence of NaH (**B**).

Based on the above experiments, we propose a plausible mechanism as shown in Scheme 4. Initially, in the presence of potassium carbonate (Scheme 6A), phenyl acetate (**1a**) is transformed into phenol salt **8** and intermediate **9**, exhibiting similar chemical properties with anhydrides [39]. Subsequently, the two-step reaction is carried out separately. On one hand, phenol salt **8** is allowed to react with bromopropanol to form a phenolic ester by S_N_2 nucleophilic substitutions. On the other hand, propanol OH with a nucleophilicity can attack the carbonyl carbon of the intermediate **9**, and release a molecule of potassium bicarbonate to form an ester bond. To verify the speculation that this reaction had two separate steps, intermediate **5** was employed to react with acetic anhydride (1 equiv), which could be transformed to **3ah** in an isolated yield of 73%. The 1-butanol (**2f**) was used to react with substrate **1v** under the standard conditions, resulting in the products **10** and **11**. Based on these observations, we came to the conclusion that the oxygen atom of the alcoholic hydroxyl group attacked the carbonyl carbon atom to form the ester bond. Additionally, when NaH was used as a base, the reaction might be carried out according to the following route (Scheme 6B): In the presence of NaH, 3-bromopropanol (**2a**) deprotonated to form the oxygen negative intermediate, **12**, which then nucleophilically attacked the phenolic ester bond of the compound, **1a**, to form the intermediates, **13** and **14**. Subsequently, intermediates **13** and **14** underwent a S_N_2 nucleophilic substitution reaction to produce the target compound, **3aa**.

**Scheme 7:**
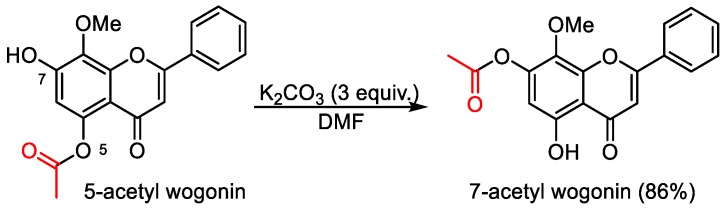
The acyl of 5-*O*-wogonin was transferred to the 7-position, forming 7-acetyl wogonin in the presence of potassium carbonate.

Given the low selectivity of the intermediate **9** reacting with the alcoholic hydroxyl group, we wondered whether the hydroxyl group in the own phenolic ester structure would also be reactive substrates without additional halogenated alcohols. 5-Acetyl wogonin was used as a substrate to study the intramolecular nucleophilic substitution reaction (Scheme 7). Gratifyingly, the desired target product, 7-acetyl wogonin, was obtained in good yields without 5-acetyl wogonin remaining under standard conditions. A possible explanation for the acyl transfer phenomenon was that the acetyl group was detached from the 5-*O*-wogonin in the presence of potassium carbonate, subsequently attacked by the highly nucleophilic 7-OH of wogonin to give 7-acetyl wogonin. The reason might be the formation of the intramolecular hydrogen bond at 5-position with para-carbonyl group endowed it with weaker acidity than 7-OH of wogonin, leading the acetyl group to easily binding to 7-OH.

## 3. Materials and Methods

### 3.1. General

All reactions were monitored by thin layer chromatography (TLC) on WhatmanPartisil^®^ K6F TLC plates (silica gel 60 Å, 0.25 mm thickness, Qingdao Haiyang Chemical Plant, Qingdao, China) and visualized using a UV lamp (254 or 365 nm, Shanghai Guanghao Analysis Instrument Co., Ltd., Shanghai, China) or by use of one of the following visualization reagents: Products were isolated by column chromatography (Merck silica gel 100–200 mesh, Merck, Darmstadt, Germany). Yields refer to chromatographically and spectroscopically homogenous materials unless noted otherwise. ^1^H- and ^13^C-NMR spectra were recorded on Bruker 300 MHz spectrometers (Bruker, Karlsruhe, Germany).

### 3.2. General Procedure for the Aryloxyalkyl Esters from Phenolic Esters *(**3aa**–**3aq**)*

Phenyl esters **1a**–**1c** (0.5 mmol) were loaded into a flask (10 mL). DMF (2 mL) and Cs_2_CO_3_ (488 mg, 1.5 mmol, 3.0 equiv) were then added, which was followed by the addition of 3-bromoethanol (**2a**, 0.525 mmol, 1.05 equiv). Then the reaction mixture was stirred at 80 °C for 6 h under an Ar atmosphere. After completion of the reaction, as confirmed by TLC, the reaction mixture was cooled down to room temperature and 10 mL of CH_2_Cl_2_ (DCM) and 10 mL of water were added. After separation of the dichloromethane layer from the water, the aqueous phase was extracted with CH_2_Cl_2_ (2 × 5 mL) again. The combined organic layers were then dried over anhydrous Na_2_SO_4_, filtered, and concentrated under vacuum to yield the crude product. The crude product was purified by silica gel column chromatography to obtain the desired pure compound.

*3-Phenoxypropyl acetate* (**3aa**). Compound **3aa** (CAS: 58883-98-0) was prepared following the general procedure described above and purified by silica gel column chromatography (DCM:MeOH 200:1) to provide the pure compound (73 mg, 76%) as a colorless oil. ^1^H-NMR (300 MHz, CDCl_3_, δ ppm): 7.33 (t, *J* = 8.2 Hz, 2H), 6.93–6.99 (m, 1H), 6.93 (d, *J* = 8.6 Hz, 2H), 4.31 (t, *J* = 6.1 Hz, 2H), 4.07 (t, *J* = 6.1 Hz, 2H), 2.13–2.19 (m, 2H), 2.09 (s, 3H); ^13^C-NMR (75 MHz, CDCl_3_, δ ppm): 170.7, 158.2, 129.1, 120.3, 113.9, 63.6, 60.9, 28.1, 20.4; MS (ESI): *m*/*z* = 193.1 [M − H]^−^; R_f_ = 0.5 (DCM:MeOH 100:1).

*3-(4-Methoxyphenoxy)propyl acetate* (**3ab**). Compound **3ab** was prepared following the general procedure described above and purified by silica gel column chromatography (DCM:MeOH 200:1) to provide the pure compound (71 mg, 64%) as a colorless oil. ^1^H-NMR (300 MHz, CDCl_3_, δ ppm): 6.85 (s, 4H, Ar-H), 4.27 (t, *J* = 6.1 Hz, 2H), 4.01 (t, *J* = 6.1 Hz, 2H), 3.78 (s, 3H, -OCH_3_), 2.08–2.14 (m, 2H), 2.07 (s, 3H, -COCH_3_); ^13^C-NMR (75 MHz, CDCl_3_, δ ppm): 196.8, 171.0, 162.6, 130.6, 130.4, 114.1, 64.6, 61.0, 28.4, 26.3, 20.9; HRMS (ESI): *m*/*z* calcd. for C_12_H_17_O_4_: 225.1121, found: 225.1119 [M + H]^+^; R_f_ = 0.5 (DCM:MeOH 100:1).

*3-(4-Nitrophenoxy)propyl acetate* (**3ac**). Compound **3ac** (CAS: 99986-51-3) was prepared following the general procedure described above and purified by silica gel column chromatography (DCM:MeOH 100:1) to provide the pure compound (96 mg, 81%) as a light yellow oil. ^1^H-NMR (300 MHz, CDCl_3_, δ ppm): 8.17 (d, *J* = 8.1 Hz, 2H), 6.5 (d, *J* = 8.1 Hz, 2H), 4.26 (t, *J* = 6.1 Hz, 2H), 4.14 (t, *J* = 6.1 Hz, 2H), 2.12–2.20 (m, 2H), 2.05 (s, 3H); ^13^C-NMR (75 MHz, CDCl_3_, δ ppm): 170.4, 163.2, 140.9, 125.3, 113.9, 64.7, 60.3, 27.8, 20.4; MS (ESI): *m/z =* 238.1 [M − H]^−^; R_f_ = 0.2 (DCM:MeOH 100:1).

*3-(4-Formylphenoxy)propyl acetate* (**3ad**). Compound **3ad** was prepared following the general procedure described above and purified by silica gel column chromatography (DCM:MeOH 50:1) to provide the pure compound (54 mg, 49%) as a colorless oil. ^1^H-NMR (300 MHz, CDCl_3_, δ ppm): 9.83 (s, 1H, -CHO), 7.78 (d, *J* = 8.1 Hz, 2H), 6.97 (d, *J* = 8.1 Hz, 2H), 4.11 (t, *J* = 6.1 Hz, 2H), 4.04 (t, *J* = 6.1 Hz, 2H), 2.02 (s, 3H, -COCH_3_), 1.77–1.89 (m, 2H); ^13^C-NMR (75 MHz, CDCl_3_, δ ppm): 190.3, 170.6, 163.4, 131.5, 129.3, 114.1, 67.1, 63.4, 24.7, 20.4; MS (ESI): *m*/*z* = 221.1 [M − H]^−^; HRMS (ESI): *m*/*z* calcd. for C_12_H_15_O_4_: 223.0965, found: 223.0961 [M + H]^+^; R_f_ = 0.3 (DCM:MeOH 50:1).

*3-(4-Acetylphenoxy)propyl acetate* (**3ae**). Compound **3ae** (CAS: 146274-20-6) was prepared following the general procedure described above and purified by silica gel column chromatography (DCM:MeOH 100:1) to provide the pure compound (66 mg, 57%) as a colorless oil. ^1^H-NMR (300 MHz, CDCl_3_, δ ppm): 7.94 (d, *J* = 8.1 Hz, 2H), 6.95 (d, *J* = 8.1 Hz, 2H), 4.27 (t, *J* = 6.1 Hz, 2H), 4.12 (t, *J* = 6.1 Hz, 2H), 2.56 (s, 3H), 2.13–2.17 (m, 2H), 2.06 (s, 3H); ^13^C-NMR (75 MHz, CDCl_3_, δ ppm): 196.8, 171.0, 162.7, 130.6, 130.4, 114.1, 64.6, 61.0, 28.4, 26.3, 20.9; MS (ESI): *m*/*z* = 235.1 [M + H]^+^; R_f_ = 0.3 (DCM:MeOH 100:1).

*3-(4-Cyanophenoxy)propyl acetate* (**3af**). Compound **3af** was prepared following the general procedure described above and purified by silica gel column chromatography (DCM:MeOH 100:1) to provide the pure compound (86 mg, 79%) as a colorless oil. ^1^H-NMR (300 MHz, CDCl_3_, δ ppm): 7.58 (d, *J* = 8.2 Hz, 2H), 6.96 (d, *J* = 8.2 Hz, 2H), 4.26 (t, *J* = 6.1 Hz, 2H), 4.09 (t, *J* = 6.1 Hz, 2H), 2.07–2.18 (m, 2H), 2.04 (s, 3H); ^13^C-NMR (75 MHz, CDCl_3_, δ ppm): 161.5, 150.4, 133.4, 118.6, 114.6, 103.5, 64.2, 60.4, 27.8, 20.4; HRMS (ESI): *m*/*z* calcd. for C_12_H_14_NO_4_: 220.0968, found: 220.0972 [M + H]^+^; R_f_ = 0.3 (DCM:MeOH 100:1).

*3-([1,1′-Biphenyl]-4-yloxy)propyl acetate* (**3ag**). Compound **3ag** (CAS: 1417800-59-9) was prepared following the general procedure described above and purified by silica gel column chromatography (DCM:MeOH 100:1) to provide the pure compound (94 mg, 70%) as a light yellow solid. ^1^H-NMR (300 MHz, CDCl_3_, δ ppm): 7.58 (d, *J* = 8.2 Hz, 2H), 7.47 (t, *J* = 6.2 Hz, 2H), 7.34–7.41 (m, 3H), 7.10 (t, *J* = 8.2 Hz, 1H), 7.05 (t, *J* = 8.2 Hz, 1H), 4.22 (t, *J* = 6.1 Hz, 2H), 4.09 (t, *J* = 6.1 Hz, 2H), 2.09–2.13 (m, 2H), 2.05 (s, 3H); ^13^C-NMR (75 MHz, CDCl_3_, δ ppm): 170.5, 155.1, 137.9, 130.5, 129.0, 128.1, 127.4, 126.4, 120.7, 112.1, 64.3, 60.8, 28.1, 20.4; MS (ESI): *m*/*z* = 293.2 [M + Na]^+^; R_f_ = 0.5 (DCM:MeOH 100:1).

*3-(Naphthalen-2-yloxy)propyl acetate* (**3ah**). Compound **3ah** (CAS: 1174495-88-5) was prepared following the general procedure described above and purified by silica gel column chromatography (DCM:MeOH 100:1) to provide the pure compound (83 mg, 68%) as a yellow solid. ^1^H-NMR (300 MHz, CDCl_3_, δ ppm): 7.78–7.84 (m, 3H), 7.51 (t, *J* = 6.2 Hz, 1H), 7.40 (t, *J* = 6.2 Hz, 1H), 7.18–7.23 (m, 2H), 4.36 (t, *J* = 6.1 Hz, 2H), 4.17 (t, *J* = 6.1 Hz, 2H), 2.17–2.25 (m, 2H), 2.12 (s, 3H); ^13^C-NMR (75 MHz, CDCl_3_, δ ppm): 170.6, 156.3, 134.1, 129.0, 128.5, 127.2, 126.3, 125.9, 123.2, 118.4, 106.1, 63.8, 60.92, 28.1, 20.5; MS (ESI): *m*/*z* = 243.1 [M − H]^−^; R_f_ = 0.6 (DCM:MeOH 100:1).

*3-(4-Chlorophenoxy)propyl benzoate* (**3ai**). Compound **3ai** was prepared following the general procedure described above and purified by silica gel column chromatography (DCM:MeOH 100:1) to provide the pure compound (71 mg, 49%) as a light yellow solid. ^1^H-NMR (300 MHz, CDCl_3_, δ ppm): 8.08 (d, *J* = 8.2 Hz, 2H), 7.58 (t, *J* = 6.2 Hz, 1H), 7.46 (t, *J* = 6.2 Hz, 2H), 7.24 (d, *J* = 8.2 Hz, 2H), 6.85 (d, *J* = 8.2 Hz, 2H), 4.55 (t, *J* = 6.1 Hz, 2H), 4.11 (t, *J* = 6.1 Hz, 2H), 2.23–2.31 (m, 2H); ^13^C-NMR (75 MHz, CDCl_3_, δ ppm): 165.9, 156.9, 132.5, 129.6, 129.0, 128.8, 127.9, 125.1, 115.2, 64.3, 61.2, 28.2; HRMS (ESI): *m*/*z* calcd. for C_16_H_16_ClO_3_: 291.0782, found: 291.0779 [M + H]^+^; R_f_ = 0.3 (DCM:MeOH 100:1).

*3-(4-Formylphenoxy)propyl benzoate* (**3aj**). Compound **3aj** (CAS: 864845-56-7) was prepared following the general procedure described above and purified by silica gel column chromatography (DCM:MeOH 50:1) to provide the pure compound (67 mg, 47%) as a yellow solid. ^1^H-NMR (300 MHz, CDCl_3_, δ ppm): 9.88 (s, 1H, -CHO), 8.04 (d, *J* = 8.6 Hz, 2H), 7.84 (d, *J* = 8.2 Hz, 2H), 7.57 (t, *J* = 6.2 Hz, 1H), 7.42 (t, *J* = 8.2 Hz, 2H), 7.01 (d, *J* = 8.2 Hz, 2H), 4.54 (t, *J* = 6.1 Hz, 2H), 4.22 (t, *J* = 6.1 Hz, 2H), 2.26–2.33 (m, 2H); ^13^C-NMR (75 MHz, CDCl_3_, δ ppm): 190.3, 159.2, 132.5, 131.5, 129.0, 127.9, 114.2, 64.4, 61.0, 28.1; MS (ESI): *m*/*z* = 283.1 [M − H]^−^; R_f_ = 0.2 (DCM:MeOH 50:1).

*3-(4-Cyanophenoxy)propyl benzoate* (**3ak**). Compound **3ak** was prepared following the general procedure described above and purified by silica gel column chromatography (DCM:MeOH 100:1) to provide the pure compound (100 mg, 71%) as yellow solids. ^1^H-NMR (300 MHz, CDCl_3_, δ ppm): 8.07 (d, *J* = 8.6 Hz, 2H), 7.61 (t, *J* = 6.2 Hz, 1H), 7.46 (t, *J* = 8.2 Hz, 2H), 7.24 (d, *J* = 8.2 Hz, 2H), 6.86 (d, *J* = 8.2 Hz, 2H), 4.55 (t, *J* = 6.1 Hz, 2H), 4.11 (t, *J* = 6.1 Hz, 2H), 2.23–2.32 (m, 2H); ^13^C-NMR (75 MHz, CDCl_3_, δ ppm): 165.8, 161.5, 133.4, 132.5, 129.5, 129.0, 127.9, 118.7, 114.7, 103.4, 64.4, 61.0, 28.0; HRMS (ESI): *m*/*z* calcd. for C_17_H_16_NO_3_: 282.1125, found: 282.1129 [M + H]^+^; R_f_ = 0.6 (DCM:MeOH 50:1).

*3-(Naphthalen-2-yloxy)propyl benzoate* (**3al**). Compound **3al** was prepared following the general procedure described above and purified by silica gel column chromatography (DCM:MeOH 100:1) to provide the pure compound (99 mg, 65%) as yellow solids. ^1^H-NMR (300 MHz, DMSO-*d*_6_, δ ppm): ^1^H-NMR (300 MHz, CDCl_3_, δ ppm): 8.05 (d, *J* = 8.6 Hz, 2H), 7.78–7.85 (m, 3H), 7.60 (t, *J* = 6.2 Hz, 1H), 7.51 (t, *J* = 6.2 Hz, 1H), 7.37 (t, *J* = 6.2 Hz, 1H), 7.26 (d, *J* = 8.2 Hz, 2H), 6.93 (d, *J* = 8.2 Hz, 2H), 4.62 (t, *J* = 6.1 Hz, 2H), 4.29 (t, *J* = 6.1 Hz, 2H), 2.31–2.40 (m, 2H); ^13^C-NMR (75 MHz, CDCl_3_, δ ppm): 166.1, 162.4, 133.9, 131.7, 129.1, 128.2, 127.6, 127.1, 126.8, 126.2, 123.6, 122.7, 118.9, 113.2, 106.3, 63.8, 61.4, 27.9; HRMS (ESI): *m*/*z* calcd. for C_20_H_19_O_3_: 307.1329, found: 307.1335 [M + H]^+^; R_f_ = 0.4 (DCM:MeOH 100:1).

*3-(Naphthalen-2-yloxy)propyl 4-methoxybenzoate* (**3am**). Compound **3am** was prepared following the general procedure described above and purified by silica gel column chromatography (DCM:MeOH 100:1) to provide the pure compound (114 mg, 68%) as yellow solids. ^1^H-NMR (300 MHz, CDCl_3_, δ ppm): 8.07 (d, *J* = 8.6 Hz, 2H), 7.76–7.83 (m, 3H), 7.49 (t, *J* = 6.2 Hz, 1H), 7.39 (t, *J* = 6.2 Hz, 1H), 7.23 (d, *J* = 8.2 Hz, 2H), 6.95 (d, *J* = 8.2 Hz, 2H), 4.60 (t, *J* = 6.1 Hz, 2H), 4.27 (t, *J* = 6.1 Hz, 2H), 3.86 (s, 3H, -OCH_3_), 2.36–2.42 (m, 2H); ^13^C-NMR (75 MHz, CDCl_3_, δ ppm): 165.8, 162.9, 156.3, 134.1, 131.2, 129.0, 128.5, 127.2, 126.3, 125.9, 123.2, 122.1, 118.5, 113.2, 106.1, 64.0, 61.1, 54.9, 28.3; HRMS (ESI): *m*/*z* calcd. for C_21_H_21_O_4_: 337.1434, found: 337.1431 [M + H]^+^; R_f_ = 0.3 (DCM:MeOH 100:1).

*3-(Naphthalen-2-yloxy)propyl 4-nitrobenzoate* (**3an**). Compound **3an** was prepared following the general procedure described above and purified by silica gel column chromatography (DCM:MeOH 50:1) to provide the pure compound (108 mg, 62%) as yellow solids. ^1^H-NMR (300 MHz, CDCl_3_, δ ppm): 8.28 (d, *J* = 8.6 Hz, 2H), 8.21 (d, *J* = 8.6 Hz, 2H), 7.72–7.80 (m, 3H), 7.46 (t, *J* = 6.2 Hz, 1H), 7.37 (t, *J* = 6.2 Hz, 1H), 7.17 (d, *J* = 8.2 Hz, 2H), 4.66 (t, *J* = 6.1 Hz, 2H), 4.28 (t, *J* = 6.1 Hz, 2H), 2.36–2.42 (m, 2H); ^13^C-NMR (75 MHz, CDCl_3_, δ ppm): 164.1, 156.1, 149.9, 133.9, 130.5, 130.2, 129.0, 128.5, 127.1, 126.2, 125.9, 123.2, 123.0, 118.2, 106.0, 63.7, 62.4, 28.1; HRMS (ESI): *m*/*z* calcd. for C_20_H_18_NO_5_: 352.1179, found: 352.1175 [M + H]^+^; R_f_ = 0.2 (DCM:MeOH 100:1).

*3-(4-Formylphenoxy)propyl 2-(2-chlorophenyl)acetate* (**3ao**). Compound **3ao** was prepared following the general procedure described above and purified by silica gel column chromatography (DCM:MeOH 50:1) to provide the pure compound (74 mg, 45%) as yellow solids. ^1^H-NMR (300 MHz, CDCl_3_, δ ppm): 9.87 (s, 2H, -CHO), 7.83 (d, *J* = 8.6 Hz, 2H), 7.35 (t, *J* = 6.2 Hz, 1H), 7.26–7.29 (m, 1H), 7.19–7.23 (m, 2H), 6.96 (d, *J* = 8.2 Hz, 2H), 4.33 (t, *J* = 6.1 Hz, 2H), 4.06 (t, *J* = 6.1 Hz, 2H), 3.78 (s, 2H), 2.12–2.16 (m, 2H); ^13^C-NMR (75 MHz, CDCl_3_, δ ppm): 190.2, 169.9, 163.2, 131.8, 131.4, 130.9, 129.5, 129.4, 128.9, 128.2, 126.4, 114.2, 64.0, 60.9, 38.7, 27.8; HRMS (ESI): *m*/*z* calcd. for C_18_H_18_ClO_4_: 333.0888, found: 333.0892 [M + H]^+^; R_f_ = 0.4 (DCM:MeOH 50:1).

*3-(4-Cyanophenoxy)propyl (E)-3-(4-chlorophenyl)acrylate* (**3ap**). Compound **3ap** was prepared following the general procedure described above and purified by silica gel column chromatography (DCM:MeOH 40:1) to provide the pure compound (71 mg, 42%) as yellow solids. ^1^H-NMR (300 MHz, CDCl_3_, δ ppm): 7.63 (d, *J* = 15.6 Hz, 1H), 7.58 (d, *J* = 8.1 Hz, 2H), 7.43 (d, *J* = 8.2 Hz, 2H), 7.32 (d, *J* = 8.2 Hz, 2H), 6.95 (d, *J* = 8.1 Hz, 2H), 6.39 (d, *J* = 15.6 Hz, 1H), 4.40 (t, *J* = 6.1 Hz, 2H), 4.13 (t, *J* = 6.1 Hz, 2H), 2.17–2.6 (m, 2H); ^13^C-NMR (75 MHz, CDCl_3_, δ ppm): 166.5, 162.0, 143.6, 136.2, 133.9, 132.7, 129.2, 129.1, 119.2, 118.3, 115.1, 104.0, 64.8, 61.3, 28.4; HRMS (ESI): *m*/*z* calcd. for C_19_H_17_ClNO_3_: 342.0891, found: 342.0896 [M + H]^+^; R_f_ = 0.3 (DCM:MeOH 50:1).

*3-(Naphthalen-2-yloxy)propyl cinnamate* (**3aq**). Compound **3aq** was prepared following the general procedure described above and purified by silica gel column chromatography (DCM:MeOH 50:1) to provide the pure compound (84 mg, 51%) as yellow solids. ^1^H-NMR (300 MHz, DMSO-*d*_6_, δ ppm): ^1^H-NMR (300 MHz, CDCl_3_, δ ppm): 7.74–7.85 (m, 4H), 7.53–7.59 (m, 2H), 7.49 (t, *J* = 6.2 Hz, 1H), 7.36–7.47 (m, 4H), 7.20–7.24 (m, 2H), 6.52 (d, *J* = 15.6 Hz, 1H), 4.53 (t, *J* = 6.1 Hz, 2H), 4.27 (t, *J* = 6.1 Hz, 2H), 2.27–2.37 (m, 2H); ^13^C-NMR (75 MHz, CDCl_3_, δ ppm): 166.5, 156.3, 144.5, 134.1, 133.8, 129.8, 128.9, 128.4, 127.6, 127.3, 127.2, 126.3, 125.9, 123.2, 118.4, 117.5, 106.1, 63.9, 61.0, 28.3; HRMS (ESI): *m*/*z* calcd. for C_22_H_21_O_3_: 333.1485, found: 333.1487 [M + H]^+^; R_f_ = 0.4 (DCM:MeOH 50:1).

### 3.3. General Procedure for the Reaction of Halogenated Alcohols and Application to the Synthesis of Flavonoid Derivatives *(**3ar**–**3ba**)*

Phenyl esters **1a**–**1j** (0.5 mmol) were loaded into a flask (10 mL). DMF (4 mL) and Cs_2_CO_3_ (488 mg, 1.5 mmol, 3.0 equiv) were then added, which was followed by the addition of bromohydrin (0.525 mmol, 1.05 equiv). Then, the reaction mixture was stirred at 80 °C for 6 h under an Ar atmosphere. After completion of the reaction, as confirmed by TLC, the reaction mixture was cooled down to room temperature and 10 mL of EtOAc and 10 mL of water were added. After separation of the EtOAc layer from the water, the aqueous phase was extracted with EtOAc (2 × 5 mL) again. The combined organic layers were then dried over anhydrous Na_2_SO_4_, filtered, and concentrated under vacuum to yield the crude product. The crude product was purified by silica gel column chromatography to obtain the desired pure compound.

*2-(4-Formylphenoxy)ethyl acetate* (**3ar**). Compound **3ar** (CAS: 151270-72-3) was prepared following the general procedure described above and purified by silica gel column chromatography (DCM:MeOH 100:1) to provide the pure compound (76 mg, 73%) as a colorless oil. ^1^H-NMR (300 MHz, CDCl_3_, δ ppm): 9.84 (s, 1H, -CHO), 7.79 (d, *J* = 8.6 Hz, 2H), 6.99 (d, *J* = 8.6 Hz, 2H), 4.41 (t, *J* = 6.1 Hz, 2H), 4.22 (t, *J* = 6.1 Hz, 2H), 2.06 (s, 3H); ^13^C-NMR (75 MHz, CDCl_3_, δ ppm): 170.2, 162.8, 131.3, 129.6, 121.8, 114.2, 65.6, 61.8, 20.2; MS (ESI): *m*/*z* = 207.1 [M − H]^−^; R_f_ = 0.3 (DCM:MeOH 50:1).

*2-(4-Cyanophenoxy)ethyl acetate* (**3as**). Compound **3as** (CAS: 150195-05-4) was prepared following the general procedure described above and purified by silica gel column chromatography (DCM:MeOH 100:1) to provide the pure compound (79 mg, 77%) as a colorless oil. ^1^H-NMR (300 MHz, CDCl_3_, δ ppm): 7.56 (d, *J* = 8.6 Hz, 2H), 6.95 (d, *J* = 8.6 Hz, 2H), 4.41 (t, *J* = 6.1 Hz, 2H), 4.20 (t, *J* = 6.1 Hz, 2H), 2.07 (s, 3H); ^13^C-NMR (75 MHz, CDCl_3_, δ ppm): 170.3, 161.2, 133.4, 118.5, 114.7, 103.7, 65.6, 61.7, 20.3; MS (ESI): *m*/*z* = 204.1 [M − H]^−^; R_f_ = 0.3 (DCM:MeOH 100:1).

*2-(4-Formylphenoxy)ethyl benzoate* (**3at**). Compound **3at** was prepared following the general procedure described above and purified by silica gel column chromatography (DCM:MeOH 100:1) to provide the pure compound (94 mg, 70%) as a colorless oil. ^1^H-NMR (300 MHz, CDCl_3_, δ ppm): 9.91 (s, 1H, -CHO), 8.07 (d, *J* = 8.1 Hz, 2H), 7.87 (d, *J* = 8.1 Hz, 2H), 7.59 (t, *J* = 8.1 Hz, 1H), 7.43–7.48 (m, 2H), 7.07 (d, *J* = 8.1 Hz, 2H), 4.72 (t, *J* = 6.1 Hz, 2H), 4.41 (t, *J* = 6.1 Hz, 2H); ^13^C-NMR (75 MHz, CDCl_3_, δ ppm): 165.9, 163.0, 132.7, 131.9, 131.5, 129.8, 129.2, 127.9, 115.8, 114.3, 65.7, 62.4; HRMS (ESI): *m*/*z* calcd. for C_16_H_15_O_4_: 271.0965, found: 271.0971 [M + H]^+^; R_f_ = 0.2 (DCM:MeOH 50:1).

*2-(Naphthalen-1-yloxy)ethyl 4-methoxybenzoate* (**3au**). Compound **3au** was prepared following the general procedure described above and purified by silica gel column chromatography (DCM:MeOH 100:1) to provide the pure compound (104 mg, 65%) as light yellow solids. ^1^H-NMR (300 MHz, CDCl_3_, δ ppm): 8.10 (d, *J* = 8.1 Hz, 2H), 7.77–7.84 (m, 3H), 7.51 (t, *J* = 8.1 Hz, 1H), 7.40 (t, *J* = 8.1 Hz, 1H), 7.25 (m, 1H), 6.93 (d, *J* = 8.1 Hz, 2H), 4.75 (t, *J* = 6.1 Hz, 2H), 4.42 (t, *J* = 6.1 Hz, 2H), 3.85 (s, 3H, -OCH_3_); ^13^C-NMR (75 MHz, CDCl_3_, δ ppm): 166.3, 163.5, 156.6, 134.5, 131.8, 129.6, 129.1, 127.7, 126.8, 126.5, 123.9, 122.3, 118.9, 113.7, 106.9, 66.1, 63.2, 55.4; HRMS (ESI): *m*/*z* calcd. for C_20_H_19_O_4_: 323.1278, found: 323.1273 [M + H]^+^; R_f_ = 0.4 (DCM:MeOH 50:1).

*4-(Naphthalen-1-yloxy)butyl 4-methoxybenzoate* (**3av**). Compound **3av** was prepared following the general procedure described above and purified by silica gel column chromatography (DCM:MeOH 100:1) to provide the pure compound (89 mg, 51%) as light yellow solids. ^1^H-NMR (300 MHz, CDCl_3_, δ ppm): 8.02 (d, *J* = 8.2 Hz, 2H), 7.79 (d, *J* = 8.1 Hz, 2H), 7.73 (d, *J* = 8.1 Hz, 2H), 7.46 (t, *J* = 8.1 Hz, 1H), 7.36 (t, *J* = 8.1 Hz, 1H), 7.18 (m, 1H), 6.91 (d, *J* = 8.2 Hz, 2H), 4.43 (t, *J* = 6.1 Hz, 2H), 4.18 (t, *J* = 6.1 Hz, 2H), 3.86 (s, 3H, -OCH_3_), 2.03–2.05 (m, 4H); ^13^C-NMR (75 MHz, CDCl_3_, δ ppm): 165.9, 162.8, 156.3, 147.0, 134.0, 131.0, 128.9, 127.1, 126.2, 125.8, 123.0, 122.2, 118.4, 113.0, 106.1, 66.8, 63.9, 54.9, 25.5, 25.1; HRMS (ESI): *m*/*z* calcd. for C_22_H_23_O_4_: 351.1591, found: 351.1587 [M + H]^+^; R_f_ = 0.4 (DCM:MeOH 50:1).

*4-(4-Chlorophenoxy)butyl acetate* (**3aw**). Compound **3aw** was prepared following the general procedure described above and purified by silica gel column chromatography (DCM:MeOH 100:1) to provide the pure compound (65 mg, 54%) as a colorless oil. ^1^H-NMR (300 MHz, CDCl_3_, δ ppm): 7.23 (d, *J* = 8.2 Hz, 2H), 6.82 (d, *J* = 8.2 Hz, 2H), 4.15 (t, *J* = 6.1 Hz, 2H), 3.95 (t, *J* = 6.1 Hz, 2H), 2.07 (s, 3H), 1.82–1.86 (m, 4H); ^13^C-NMR (75 MHz, CDCl_3_, δ ppm): 157.0, 128.7, 124.9, 116.1, 115.2, 67.0, 63.6, 25.2, 24.8, 20.4; HRMS (ESI): *m*/*z* calcd. for C_12_H_16_O_3_Cl: 243.0782, found: 243.0786 [M + H]^+^; R_f_ = 0.5 (DCM:MeOH 100:1).

*5-(4-Cyanophenoxy)pentyl benzoate* (**3ax**). Compound **3ax** was prepared following the general procedure described above and purified by silica gel column chromatography (DCM:MeOH 100:1) to provide the pure compound (60 mg, 39%) as a colorless oil. ^1^H-NMR (300 MHz, CDCl_3_, δ ppm): 8.05 (d, *J* = 8.2 Hz, 2H), 7.55–7.59 (m, 3H), 7.45 (t, *J* = 6.3 Hz, 1H), 6.94 (d, *J* = 8.2 Hz, 2H), 4.38 (t, *J* = 6.1 Hz, 2H), 4.04 (t, *J* = 6.1 Hz, 2H), 1.83–1.95 (m, 4H), 1.60–1.70 (m, 2H); ^13^C-NMR (75 MHz, CDCl_3_, δ ppm): 166.6, 162.2, 133.9, 132.9, 129.5, 128.3, 119.3, 115.1, 68.0, 64.6, 28.6, 28.4, 22.6; HRMS (ESI): *m*/*z* calcd. for C_19_H_20_O_3_N: 310.1438, found: 310.142 [M + H]^+^; R_f_ = 0.6 (DCM:MeOH 50:1).

*((5-Hydroxy-4-oxo-2-phenyl-4H-chromene-6,7-diyl)bis(oxy))bis(propane-3,1-diyl) diacetate* (**3ay**). Compound **3ay** was prepared from 6,7-diacetyl baicalein following the general procedure described above and purified by silica gel column chromatography (DCM:MeOH 50:1) to provide the pure compound (110 mg, 47%) as yellow solids. ^1^H-NMR (300 MHz, CDCl_3_, δ ppm): 12.67 (s, 1H, -OH), 7.90 (d, *J* = 8.1 Hz, 2H), 7.51–7.60 (m, 3H), 6.69 (s, 1H), 6.57 (s, 1H), 4.37 (t, *J* = 6.1 Hz, 2H), 4.33 (t, *J* = 6.1 Hz, 2H), 4.18 (t, *J* = 6.1 Hz, 2H), 4.15 (t, *J* = 6.1 Hz, 2H), 2.21–2.30 (m, 2H), 2.13 (m, 2H), 2.10 (s, 3H), 2.08 (s, 3H); HRMS (ESI): *m*/*z* calcd. for C_25_H_27_O_9_: 471.1650, found: 471.1659 [M + H]^+^; R_f_ = 0.6 (DCM:MeOH 30:1).

*2-((5,6-Dihydroxy-4-oxo-2-phenyl-4H-chromen-7-yl)oxy)ethyl acetate* (**3az**). Compound **3az** was prepared from 7-acetyl baicalein following the general procedure described above and purified by silica gel column chromatography (DCM:MeOH 40:1) to provide the pure compound (76 mg, 43%) as yellow solids. ^1^H-NMR (300 MHz, DMSO-*d*_6_, δ ppm): 12.53 (s, 1H, -OH), 8.81 (s, 1H, -OH), 8.06–8.11 (m, 2H), 7.55–7.63 (m, 3H), 7.00 (s, 1H), 6.98 (s, 1H), 4.39 (t, *J* = 6.1 Hz, 2H), 4.35 (t, *J* = 6.1 Hz, 2H), 2.07 (s, 3H); ^13^C-NMR (75 MHz, DMSO-*d*_6_, δ ppm): 182.2, 170.3, 163.1, 153.2, 149.5, 146.2, 131.9, 130.7, 130.2, 129.0, 126.2, 105.4, 104.6, 92.1, 66.9, 62.2, 20.6; HRMS (ESI): *m*/*z* calcd. for C_19_H_17_O_7_: 357.0969, found: 357.0975 [M + H]^+^; R_f_ = 0.3 (DCM:MeOH 30:1).

*3-((5-Hydroxy-8-methoxy-4-oxo-2-phenyl-4H-chromen-7-yl)oxy)propyl acetate* (**3ba**). Compound **3ba** was prepared from 7-acetyl wogonin following the general procedure described above and purified by silica gel column chromatography (DCM:MeOH 50:1) to provide the pure compound (132 mg, 69%) as yellow solids. ^1^H-NMR (300 MHz, CDCl_3_, δ ppm): 12.56 (s, 1H, -OH), 7.95 (t, *J* = 6.1 Hz, 2H), 7.55 (m, 3H), 6.69 (s, 1H), 6.44 (s, 1H), 4.33 (t, *J* = 6.1 Hz, 2H), 4.19 (t, *J* = 6.1 Hz, 2H), 3.94 (s, 3H, -OCH_3_), 2.19–2.28 (m, 2H), 2.10 (s, 3H); ^13^C-NMR (75 MHz, CDCl_3_, δ ppm): 182.7, 178.0, 164.1, 163.9, 157.9, 1574, 157.3, 131.9, 131.2, 129.1, 126.3, 126.2, 105.3, 96.5, 65.5, 61.6, 60.9, 28.4, 20.9; HRMS (ESI): *m*/*z* calcd. for C_21_H_21_O_7_: 385.1282, found: 385.1278 [M + H]^+^; R_f_ = 0.3 (DCM:MeOH 50:1).

### 3.4. Control Experiments to Investigate the Mechanism

*2-((3-Methylbut-2-en-1-yl)oxy)naphthalene* (**4**). Phenyl esters **1i** (0.5 mmol) were loaded into a flask (10 mL). DMF (2 mL) and K_2_CO_3_ (207 mg, 1.5 mmol, 3.0 equiv) were then added, which was followed by the addition of 3-methyl-2-butenyl bromide **2e** (0.525 mmol, 1.05 equiv). Then, the reaction mixture was stirred at 80 °C for 6 h under an Ar atmosphere. After completion of the reaction, as confirmed by TLC, the reaction mixture was cooled down to room temperature and 10 mL of CH_2_Cl_2_ and 10 mL of water were added. After separation of the dichloromethane layer from the water, the aqueous phase was extracted with CH_2_Cl_2_ (2 × 5 mL) again. The combined organic layers were then dried over anhydrous Na_2_SO_4_, filtered, and concentrated under vacuum to yield the crude product. The crude product was purified by silica gel column chromatography with hexane/EtOAc (8:1 *v*/*v*) to afford the desired compound, **4** (CAS: 23676-18-8), as a slightly yellow solid (96 mg, 91% yield). ^1^H-NMR (300 MHz, CDCl_3_, δ ppm): 7.94–8.01 (m, 3H), 7.69 (t, *J* = 6.1 Hz, 1H), 7.58 (t, *J* = 6.1 Hz, 1H), 7.49 (dd, *J* = 8.1 Hz, 1.8 Hz, 1H), 7.40 (d, *J* = 1.8 Hz, 1H), 5.85 (t, *J* = 6.2 Hz, 1H, -CH=C), 4.83 (d, *J* = 9.1 Hz, 2H, -CH_2_-), 2.06 (s, 3H, -CH_3_), 1.99 (s, 3H, -CH_3_); ^13^C-NMR (75 MHz, CDCl_3_, δ ppm): 156.7, 137.6, 134.4, 129.2, 127.4, 126.5, 126.1, 123.3, 119.6, 118.9, 115.2, 106.4, 64.4, 25.6, 17.9; MS (ESI): *m*/*z* = 213.1 [M + H]^+^; R_f_ = 0.7 (DCM:MeOH 100:1).

*3-(Naphthalen-2-yloxy)propan-1-ol* (**5**). Phenyl ester **1i** (0.5 mmol) was loaded into a flask (10 mL). DMF (2 mL) and K_2_CO_3_ (207 mg, 1.5 mmol, 3.0 equiv) were then added, which was followed by the addition of 3-bromo-1-propanol **2a** (2.0 mmol, 4 equiv). Then, the reaction mixture was stirred at 80 °C for 6 h under an Ar atmosphere. After completion of the reaction, as confirmed by TLC, the reaction mixture was cooled down to room temperature and 10 mL of CH_2_Cl_2_ and 10 mL of water were added. After separation of the dichloromethane layer from the water, the aqueous phase was extracted with CH_2_Cl_2_ (2 × 5 mL) again. The combined organic layers were then dried over anhydrous Na_2_SO_4_, filtered, and concentrated under vacuum to yield the crude product. The crude product was purified by silica gel column chromatography with hexane/EtOAc (4:1 *v*/*v*) to afford the desired compound, **5** (CAS: 7598-29-0), as a slightly yellow oil (96 mg, 95% yield). ^1^H-NMR (300 MHz, CDCl_3_, δ ppm): 7.74–7.82 (m, 3H), 7.48 (t, *J* = 6.2 Hz, 1H), 7.38 (t, *J* = 6.2 Hz, 1H), 7.19–7.24 (m, 2H), 4.63 (br s, 1H, -OH), 4.22 (t, *J* = 6.1 Hz, 2H), 4.05 (t, *J* = 6.1 Hz, 2H), 2.16–2.19 (m, 2H); MS (ESI): *m*/*z* = 203.1 [M + H]^+^; R_f_ = 0.4 (DCM:MeOH 50:1).

*Butyl 4-methoxybenzoate* (**10**). Compound **10** (CAS: 6946-35-6) was prepared from **1v** and 1-butanol (**2f**), following the above general procedure and purified by silica gel column chromatography (DCM:MeOH 100:1) to provide the pure compound (59 mg, 57%) as a light oil. ^1^H-NMR (300 MHz, CDCl_3_, δ ppm): 8.01 (d, *J* = 8.1 Hz, 1H), 6.92 (d, *J* = 8.1 Hz, 1H), 4.30 (t, *J* = 6.2 Hz, 2H), 3.85 (s, 3H, -OCH_3_), 1.74 (m, 2H), 1.49 (m, 2H), 0.98 (t, *J* = 6.2 Hz, 3H); ^13^C-NMR (75 MHz, CDCl_3_, δ ppm): 165.9, 162.7, 131.0, 122.4, 113.0, 64.0, 54.8, 30.3, 18.7, 13.2; MS (ESI): *m*/*z* = 209.1 [M + H]^+^; R_f_ = 0.8 (DCM:MeOH 100:1).

*5-Hydroxy-8-methoxy-4-oxo-2-phenyl-4H-chromen-7-yl acetate (7-acetyl wogonin).* 5-Acetyl wogonin (65 mg, 0.2 mmol) were loaded into a flask (25 mL). DMF (1.5 mL) and K_2_CO_3_ (82 mg, 0.6 mmol, 3.0 equiv) was added. Then, the reaction mixture was stirred at 80 °C for 6 h under an Ar atmosphere. After completion of the reaction, as confirmed by TLC, the reaction mixture was cooled down to room temperature and 5 mL of CH_2_Cl_2_ and 5 mL of water were added. After separation of the CH_2_Cl_2_ layer from the water, the aqueous phase was extracted with CH_2_Cl_2_ (2 × 3 mL) again. The combined organic layers were then dried over anhydrous Na_2_SO_4_, filtered, and concentrated under vacuum to yield the crude product. The crude product was purified by silica gel column chromatography with hexane/EtOAc (5:1 *v*/*v*) to afford 7-acyl wogonin (CAS: 95480-80-1) as slightly yellow solids (56 mg, 86% yield). ^1^H-NMR (300 MHz, DMSO-*d*_6_, δ ppm): 12.49 (s, 1H, 5-OH), 7.99–8.02 (m, 2H, H-2′, H-6′), 7.63–7.69 (m, 3H, H-3′, H-4′, H-5′), 6.73 (s, 1H, H-3), 6.38 (s, 1H, H-6), 3.81 (s, 3H, 8-OCH_3_), 2.19 (s, 3H, -CH_3_); MS (ESI): *m*/*z* = 325.1 [M − H]^−^; R_f_ = 0.3 (DCM:MeOH 100:1).

## 4. Conclusions

In summary, we have developed a facile and unified one-pot synthesis to obtain aryloxyalkyl esters from readily available phenolic esters and suitable substituted halogenated alcohols, which helps to quickly build a diversity chain of aromatic alkyl esters. In the mechanistic studies, we discovered the acyl transfer mechanism and explained the reasons for the formation of aryloxyalkyl esters. However, for secondary alcohols or tertiary alcohols, the corresponding target compounds cannot be obtained under standard reaction conditions, which can be attributed to the large steric hindrance near the hydroxyl group affecting the nucleophilic substitution reaction (S_N_2) of hydroxyl groups. In addition, secondary or tertiary alcohols are more likely to undergo elimination reactions under alkaline conditions. Further detailed mechanistic studies and applications will be reported in due course.

## Figures and Tables

**Table 1:**
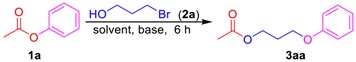
Condition optimization for aryloxyalkyl esterification. *^a^*

Entry	Base	Equiv	Solvent	Temp.	Yield (%) *^b^*
1	K_2_CO_3_	1.5	acetone	60 °C	59
2	K_2_CO_3_	1.5	THF	60 °C	51
3	K_2_CO_3_	1.5	MeCN	60 °C	54
4	K_2_CO_3_	1.5	DMF	80 °C	65
5	K_2_CO_3_	3	DMF	80 °C	72
6	-- *^c^*	--	DMF	80 °C	NR *^d^*
7	KHCO_3_	3	DMF	80 °C	13
8	Cs_2_CO_3_	3	DMF	80 °C	76
9	NaH	3	DMF	60 °C	73
10	NaOH	1.5	MeCN	60 °C	Trace

*^a^* Reaction conditions: Except where otherwise noted, all of the reactions were performed with **1a** (0.5 mmol), **2a** (0.525 mmol), and a base in the solvent (1.5 mL) for 6 h under an Ar atmosphere. *^b^* Yield of isolated product. *^c^* --: No base exists. *^d^* NR: No reaction. Thin layer chromatography (TLC) showed the presence of starting materials, **1a**, and detected no new product.

## Supplementary Materials

The following are available online, characterization data: HRMS, ^1^H-NMR, and ^13^C-NMR spectra of all new compounds.
