# Supplementary material for: Convenient Method of Synthesizing Aryloxyalkyl Esters from Phenolic Esters Using Halogenated Alcohols
Source: Molecules. 2018 Jul 13;23(7):1715. doi: 10.3390/molecules23071715 (PMC6100006; doi:10.3390/molecules23071715)
Supplement: Supplementary file 1 [file molecules-23-01715-s001.pdf]

Supporting Information

Convenient Synthesis of Aryloxyalkyl Esters from Phenolic Esters Using Halogenated Alcohol

Preparation of acetate flavonoids

5-hydroxy-4-oxo-2-phenyl-4H-chromene-6,7-diyl diacetate (6,7-diacetyl baicalein). A mixture of baicalein (1 g, 3.7 mmol) and sodium acetate (759 mg, 9.3 mmol, 2.5 equiv) were loaded into a flask (50 mL) under atmosphere of argon. Acetic anhydride (20 mL) was then added. The mixture was heated at 70 °C for 5 h and then the reaction mixture was concentrated under reduced pressure. 20 mL of EtOAc and 20 mL of water were added. The organic layer was separated and the aqueous layer was extracted with EtOAc (3 × 20 mL), and the combined organic layers were dried over anhydrous Na_2SO_4, and filtration and removal of solvent under vacuum. The crude material was purified by column chromatography (DCM: MeOH 100:1) to afford 6,7-diacetyl baicalein as slightly yellow solids (903 mg, 69%). ^{1}H NMR (300 MHz, $CDCl_3$, δ ppm): 12.83 (s, 1H, -OH), 7.92 (d, J = 8.1 Hz, 2H), 7.49-7.57 (m, 3H), 6.67 (s, 1H), 6.49 (s, 1H), 2.17 (s, 3H), 2.14 (s, 3H); MS (ESI): m/z= 353.1 $[M-H]^-$; R_f = 0.5 (DCM: MeOH 50:1).

5,6-dihydroxy-4-oxo-2-phenyl-4H-chromen-7-yl acetate (7-acetyl baicalein). A mixture of baicalein (1 g, 3.7 mmol) and sodium acetate (303 mg, 3.7 mmol, 1.0 equiv) were loaded into a flask (50 mL) under atmosphere of argon. Acetic anhydride (20 mL) was then added. The mixture was heated at 60 °C for 3 h and then the reaction mixture was concentrated under reduced pressure. 20 mL of EtOAc and 20 mL of water were added. The organic layer was separated and the aqueous layer was extracted with EtOAc (3 × 20 mL), and the combined organic layers were dried over anhydrous Na_2SO_4, and filtration and removal of solvent under vacuum. The crude material was purified by column chromatography (DCM: MeOH 80:1) to afford 7-acetyl baicalein as yellowish solids (842 mg, 73%). ^{1}H NMR (300 MHz, $CDCl_3$, δ ppm): 12.79 (s, 1H, -OH), 8.93 (s, 1H, -OH), 7.96 (d, J = 8.2 Hz, 2H), 7.50-7.59 (m, 3H), 6.57 (s, 1H), 6.38 (s, 1H), 2.20 (s, 3H); MS (ESI): m/z= 353.1 $[M-H]^-$; R_f = 0.6 (DCM: MeOH 30:1).

5-hydroxy-8-methoxy-4-oxo-2-phenyl-4H-chromen-7-yl acetate (7-acetyl wogonin). A mixture of wogonin (568 mg, 2 mmol) and sodium acetate (213 mg, 2.6 mmol, 1.3 equiv) were loaded into a flask (25 mL) under atmosphere of argon. Acetic anhydride (10 mL) was then added. The mixture was heated at 70 °C for 3 h and then the reaction mixture was concentrated under reduced pressure. 10 mL of EtOAc and 10 mL of water were added. The organic layer was separated and the aqueous layer was extracted with EtOAc (3 × 10 mL), and the combined organic layers were dried over anhydrous Na_2SO_4, and filtration and removal of solvent under vacuum. The crude material was purified by column chromatography

(DCM: MeOH 100:1) to afford 7-acetyl wogonin as yellowish solids (528 mg, 81%). ^{1}H NMR (300 MHz, $CDCl_3$, δ ppm): 12.63 (s, 1H, -OH), 7.94 (d, J = 7.9 Hz, 2H), 7.55-7.59 (m, 3H), 6.63 (s, 1H), 6.49 (s, 1H), 3.89 (s, 3H, $-OCH_3$), 2.13 (s, 3H); MS (ESI): m/z= 325.2 $[M-H]^-$; R_f = 0.5 (DCM: MeOH 50:1).

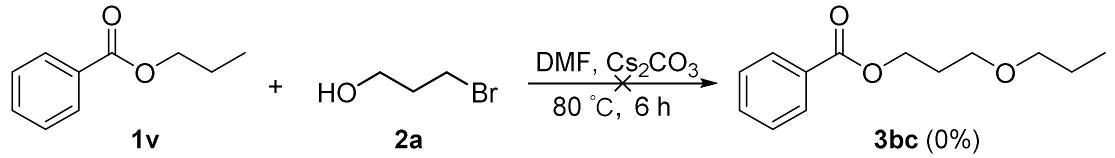

Scheme s1. Standard conditions: alkyl ester (**1v**, 82 mg, 0.5 mmol), 3-bromo-1-propanol (**2a**, 48 μL, 0.525 mmol), Cs_2CO_3 (489 mg, 1.5 mmol), 80 ℃ in DMF (1.5 mL) for 6 hrs under an Ar atmosphere. Isolated yields.

NMR spectra:

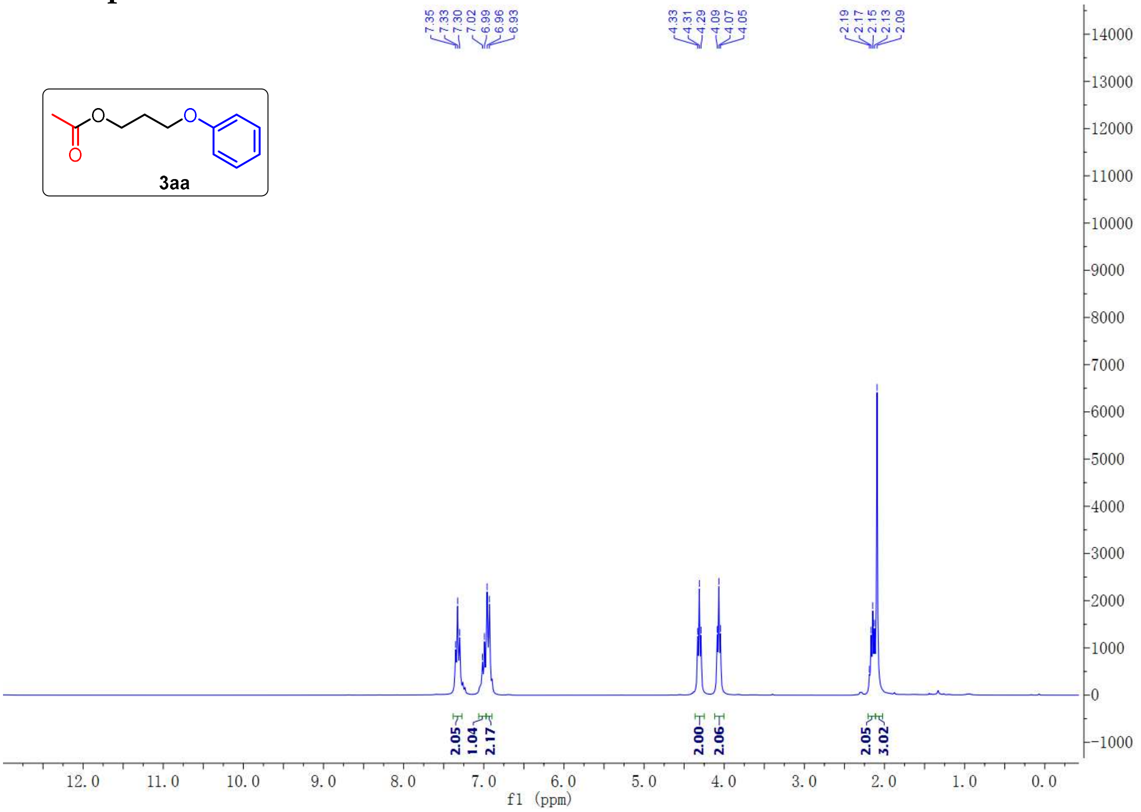

7.35
7.33
7.30
7.02
6.99
6.96
6.93
4.33
4.31
4.29
4.09
4.07
4.05
2.19
2.17
2.15
2.13
2.09
3aa
2.05
1.04
2.17
2.00
2.06
2.05
3.02
f1 (ppm)

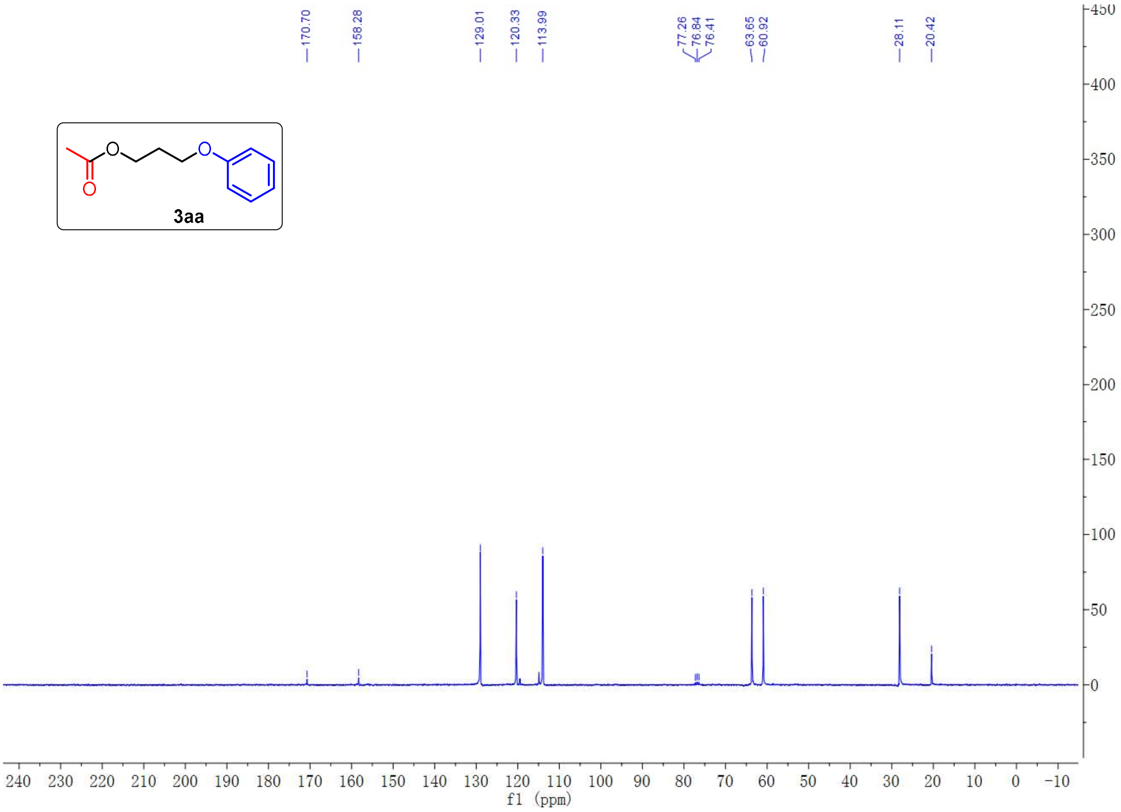

170.70
158.28
129.01
120.33
113.99
77.26
76.84
76.41
63.65
60.92
28.11
20.42
3aa
f1 (ppm)

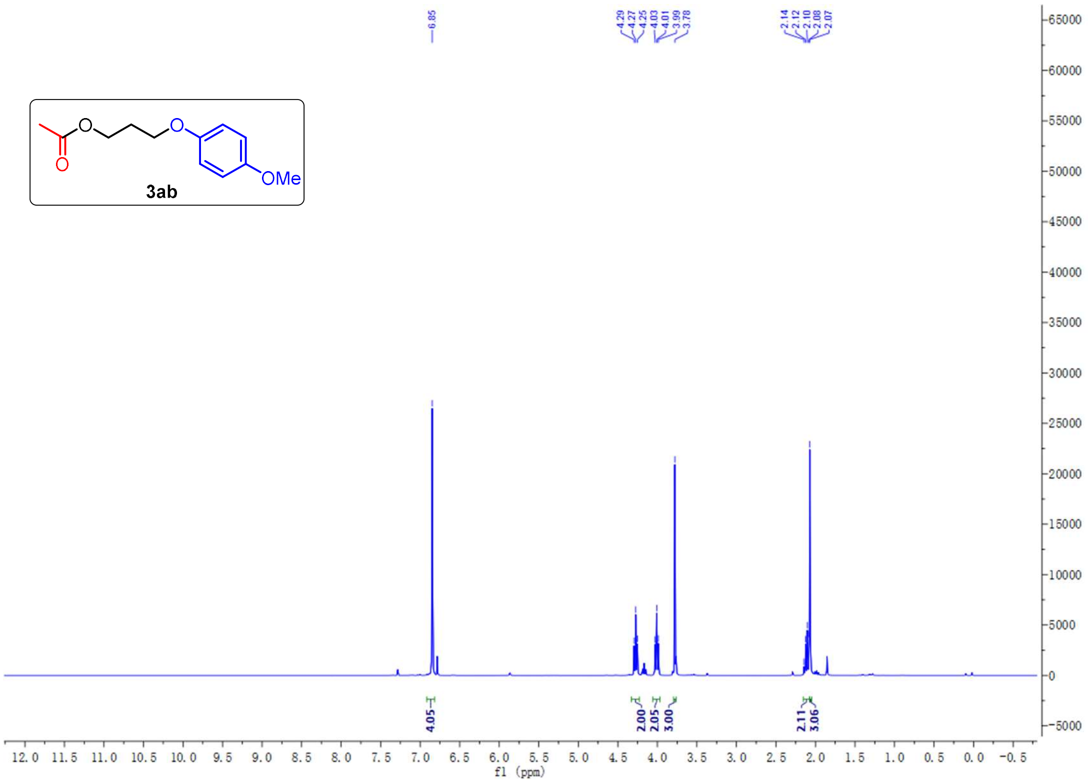
3ab
OMe
f1 (ppm)

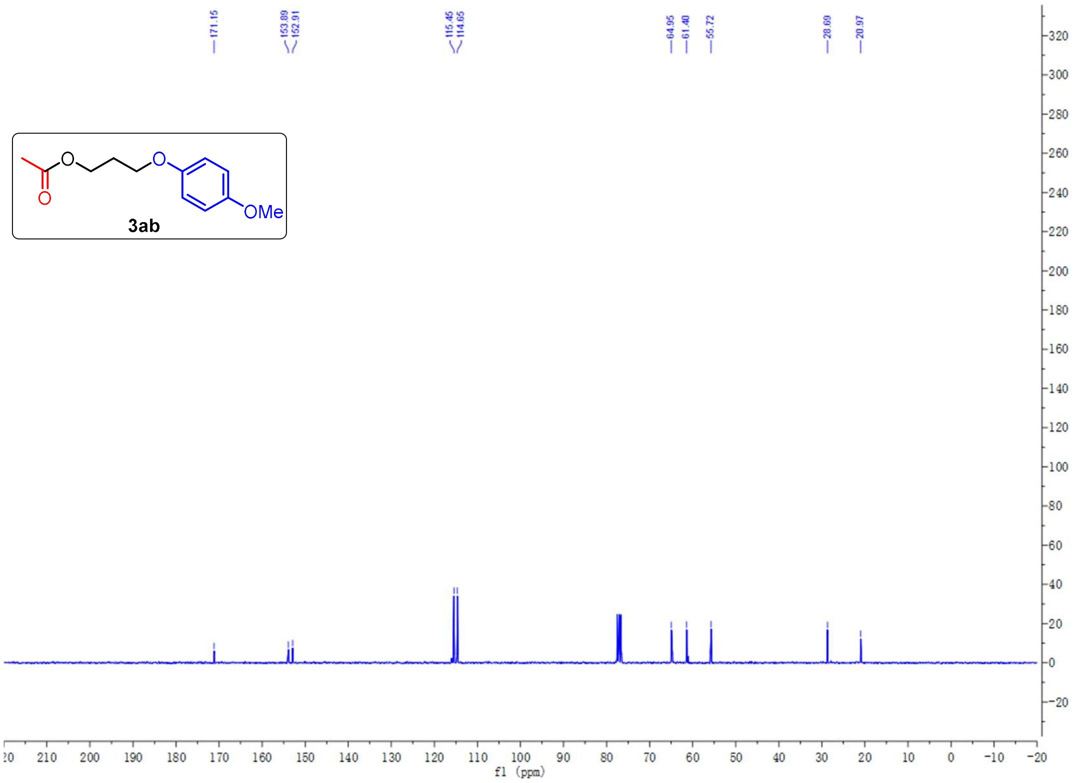
3ab
OMe
f1 (ppm)

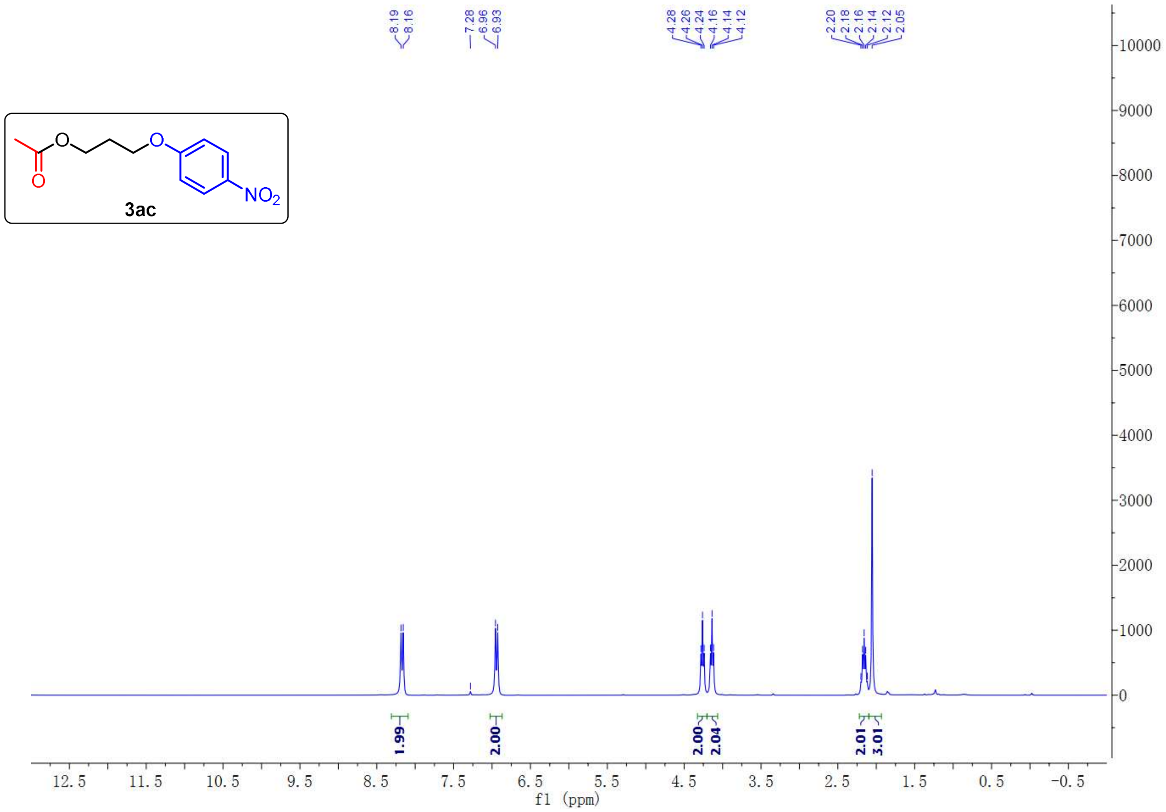
3ac
NO2
8.19
8.16
7.28
6.96
6.93
4.28
4.26
4.24
4.16
4.14
4.12
2.20
2.18
2.16
2.14
2.12
2.05
1.99
2.00
2.00
2.04
2.01
3.01
f1 (ppm)

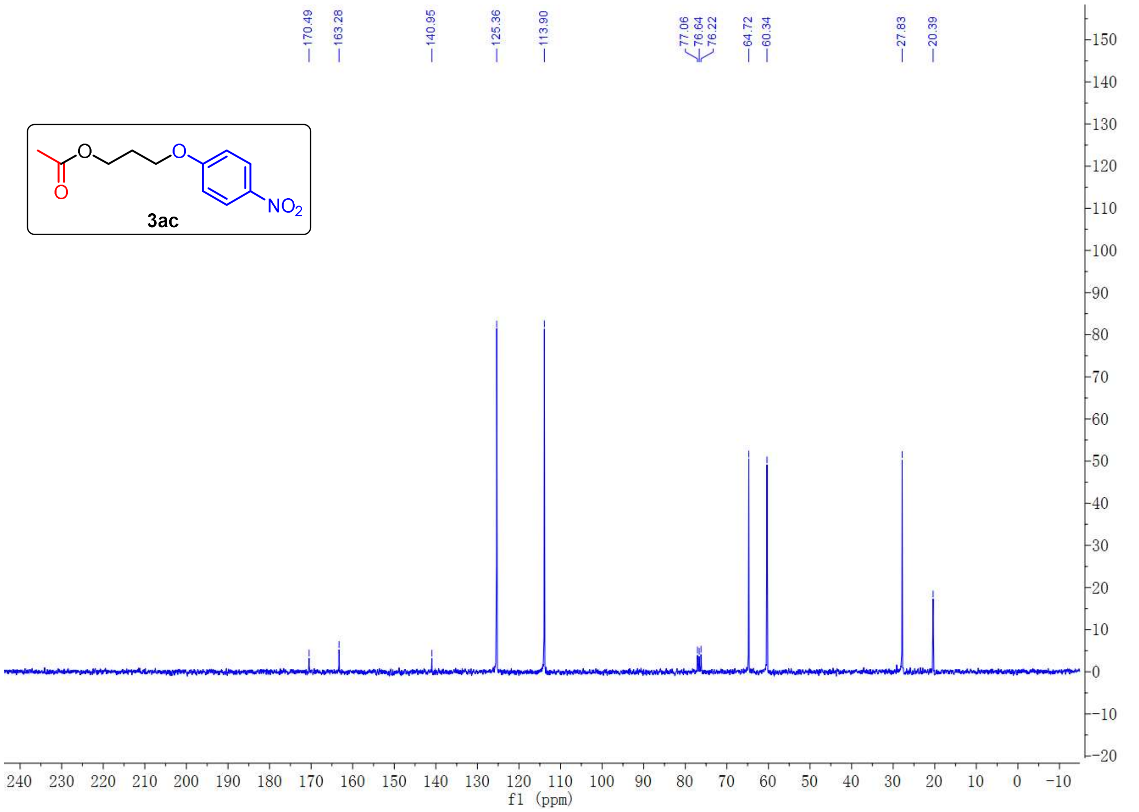
3ac
NO2
170.49
163.28
140.95
125.36
113.90
77.06
76.64
76.22
64.72
60.34
27.83
20.39
f1 (ppm)

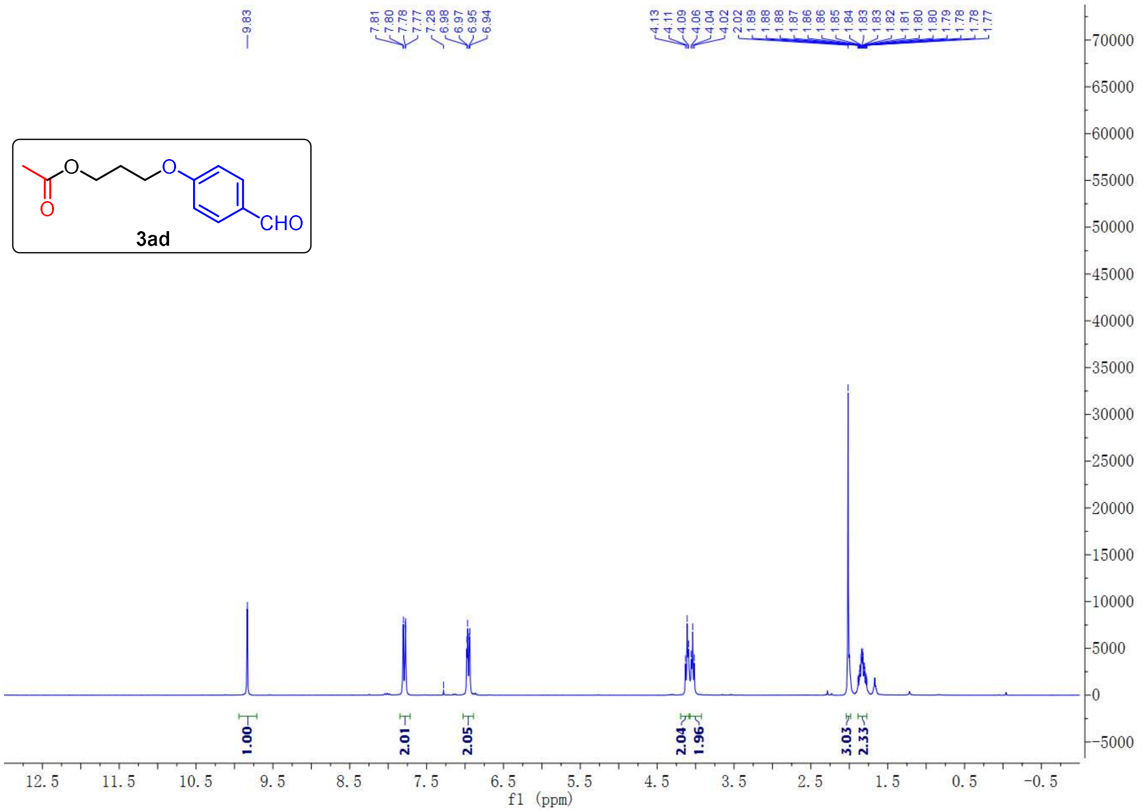

9.83
7.81
7.80
7.78
7.77
7.28
6.98
6.97
6.95
6.94
4.13
4.11
4.09
4.06
4.04
4.02
2.02
1.89
1.88
1.88
1.87
1.86
1.86
1.85
1.84
1.83
1.83
1.82
1.81
1.80
1.80
1.79
1.78
1.78
1.77
O
O
O
CHO
3ad
1.00
2.01
2.05
2.04
1.96
3.03
2.33
f1 (ppm)

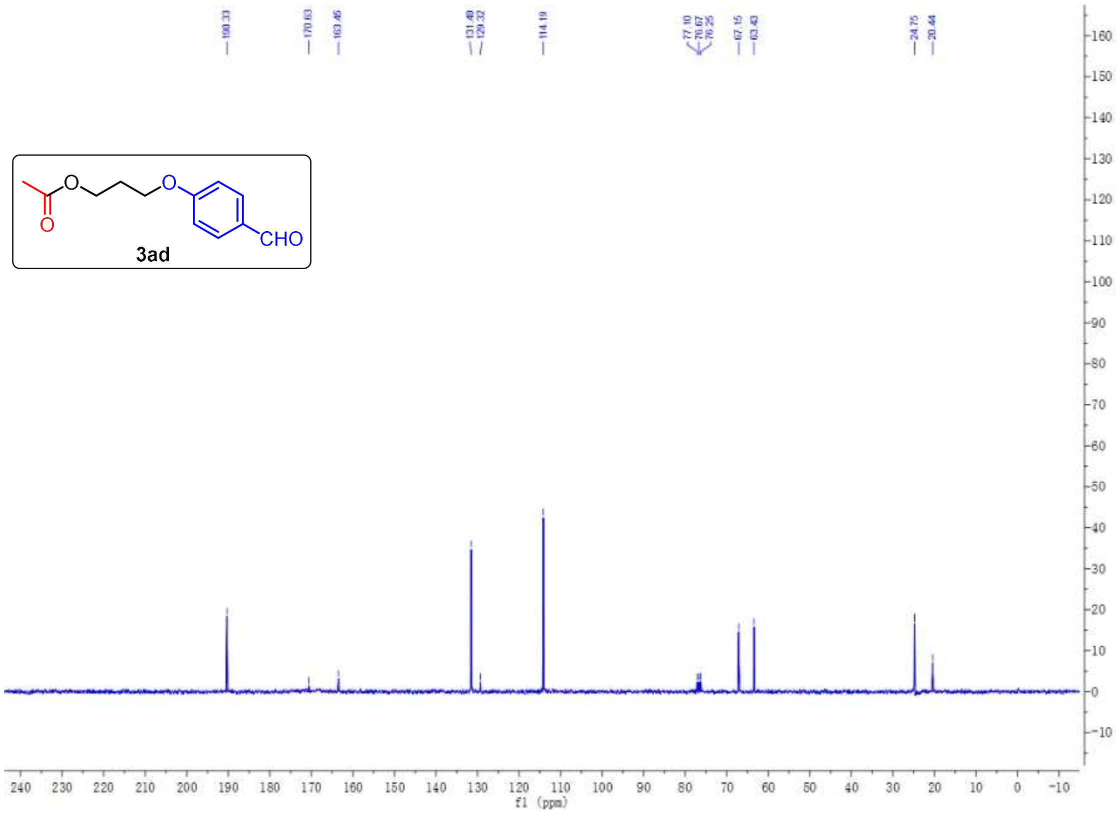

190.33
170.63
163.45
131.49
129.32
114.19
77.10
76.67
76.25
67.15
63.43
24.75
20.44
O
O
O
CHO
3ad
f1 (ppm)

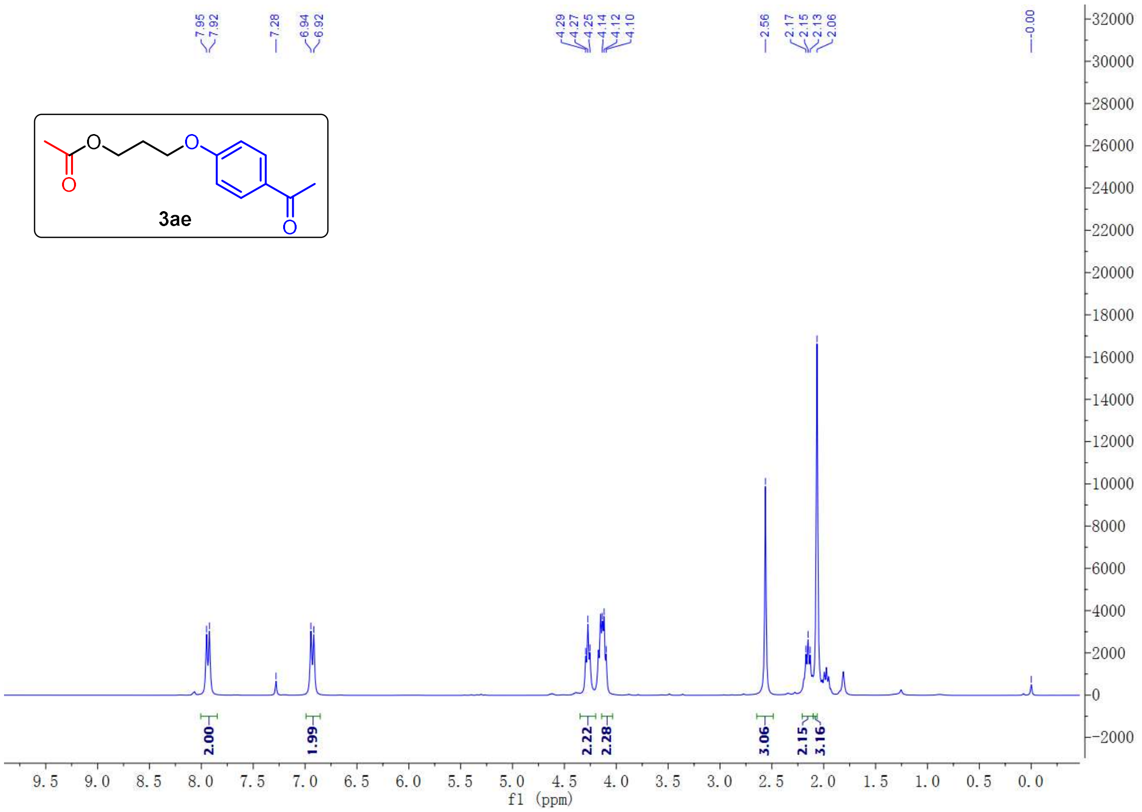
3ae
7.95
7.92
7.28
6.94
6.92
4.29
4.27
4.25
4.14
4.12
4.10
2.56
2.17
2.15
2.13
2.06
0.00
2.00
1.99
2.22
2.28
3.06
2.15
3.16
f1 (ppm)

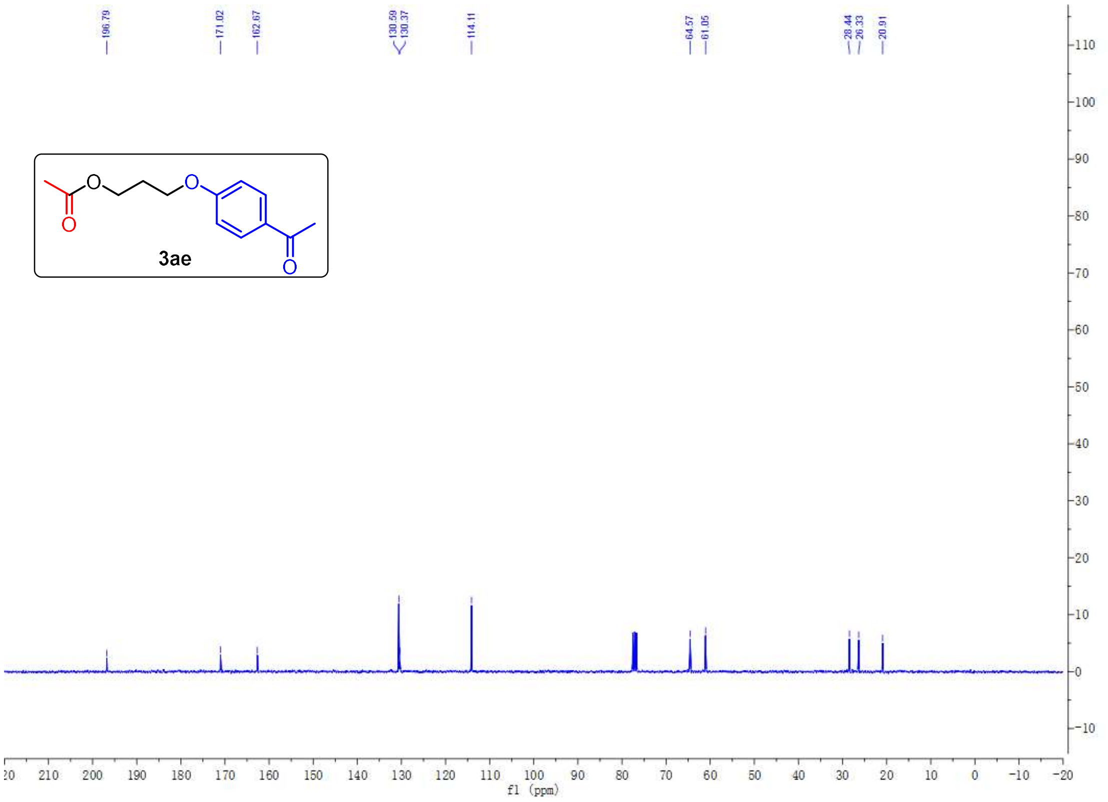
3ae
196.79
171.02
162.67
130.59
130.37
114.11
64.57
61.05
28.44
26.33
20.91
f1 (ppm)

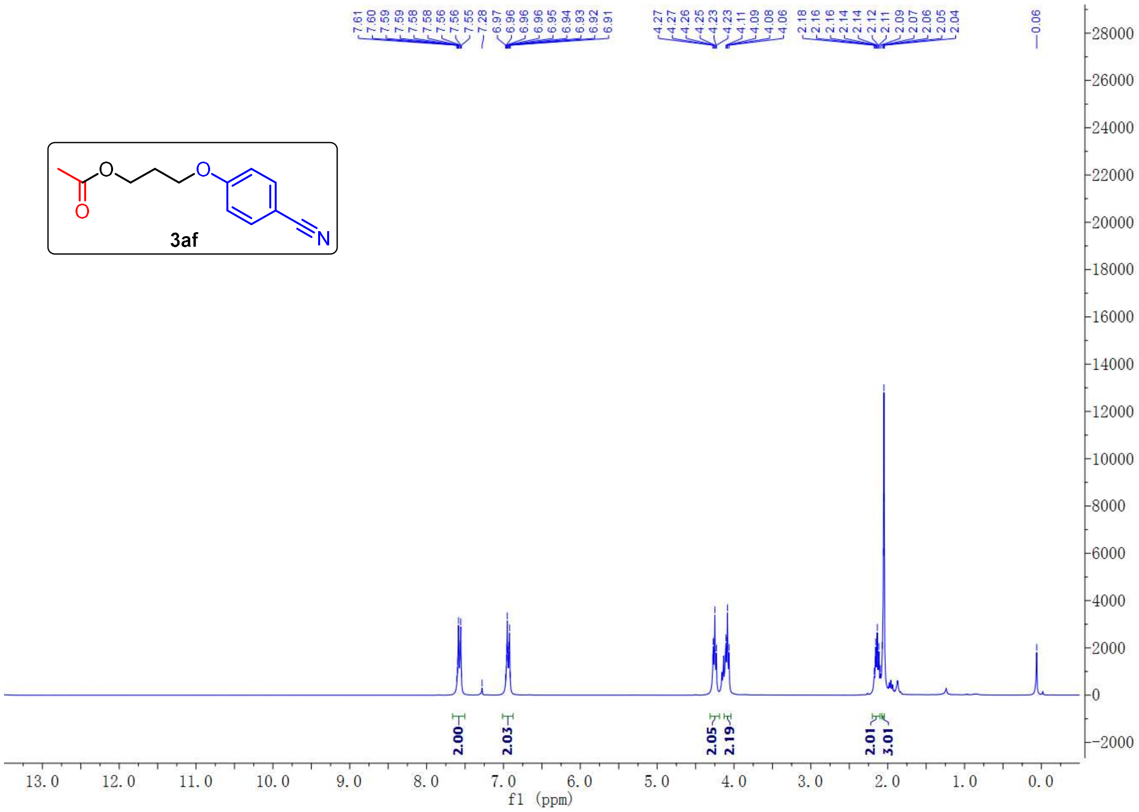
3af
f1 (ppm)

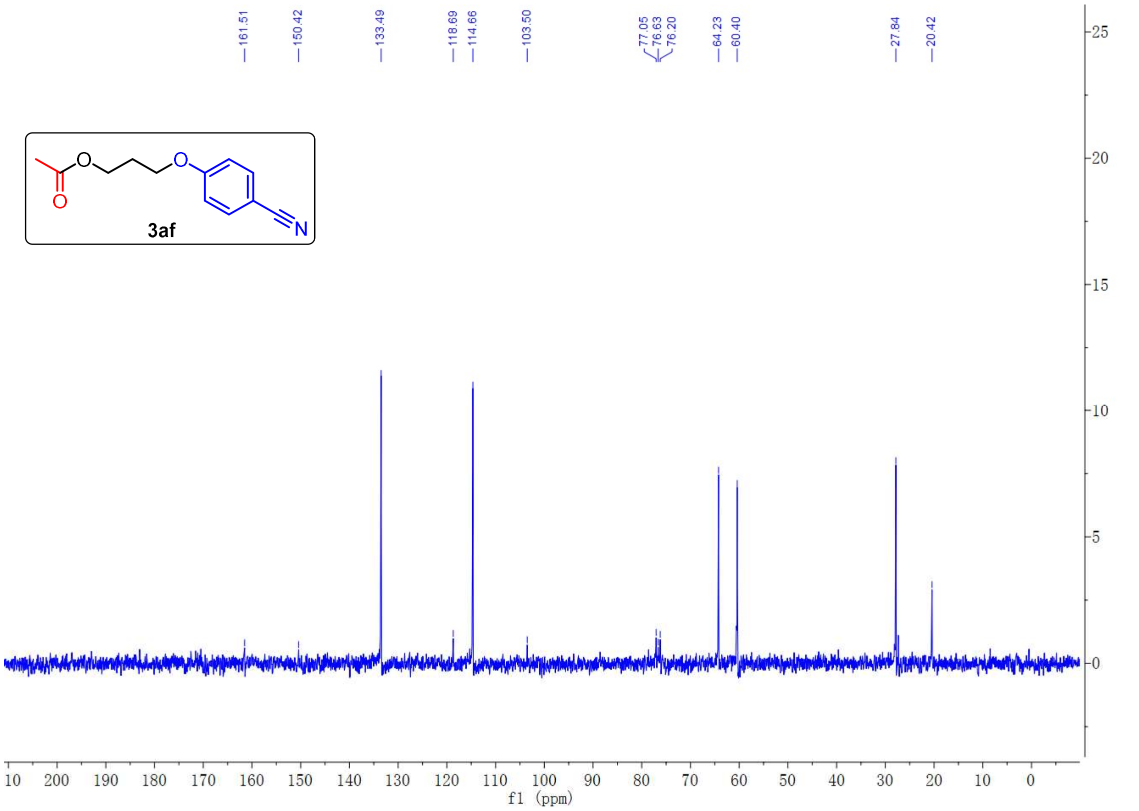
3af
161.51
150.42
133.49
118.69
114.66
103.50
77.05
76.63
76.20
64.23
60.40
27.84
20.42
f1 (ppm)

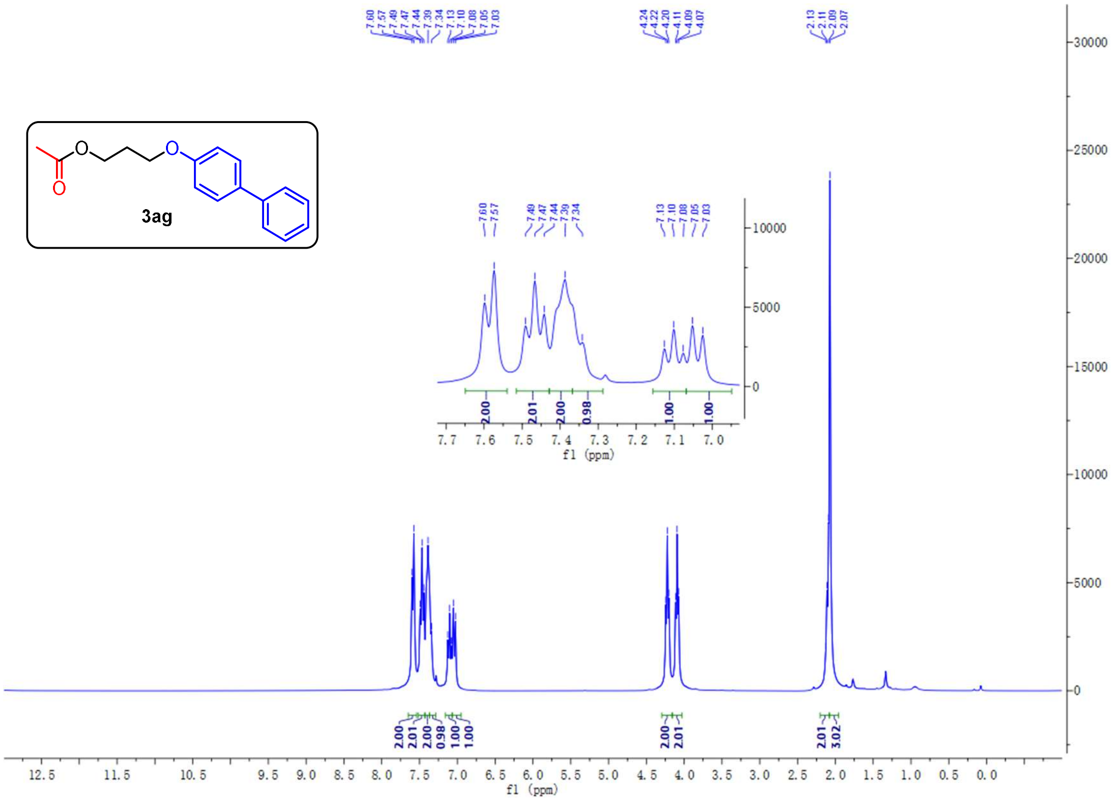
7.60 7.57 7.49 7.47 7.44 7.39 7.34 7.13 7.10 7.08 7.05 7.03
4.24 4.22 4.20 4.11 4.09 4.07
2.13 2.11 2.09 2.07
3ag
2.00 2.01 2.00 0.98 1.00 1.00
2.00 2.01
2.01 3.02
f1 (ppm)

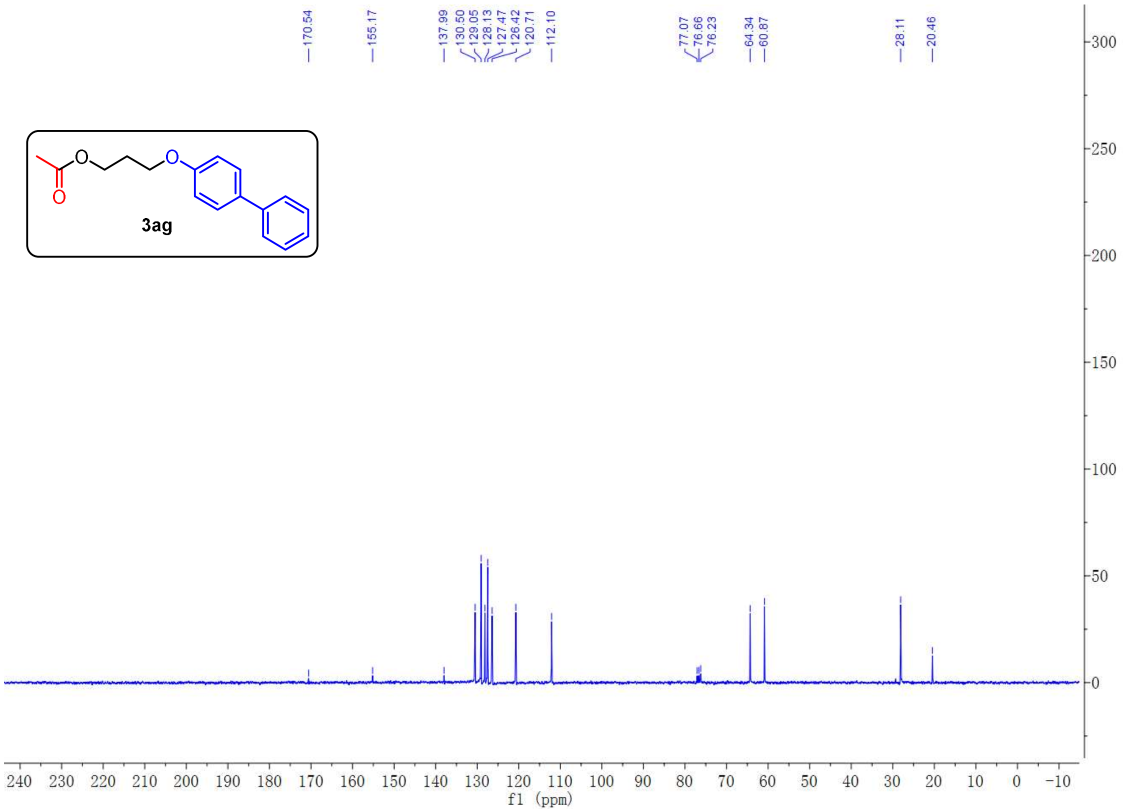
170.54
155.17
137.99 130.50 129.05 128.13 127.47 126.42 120.71
112.10
77.07 76.66 76.23
64.34 60.87
28.11
20.46
3ag
f1 (ppm)

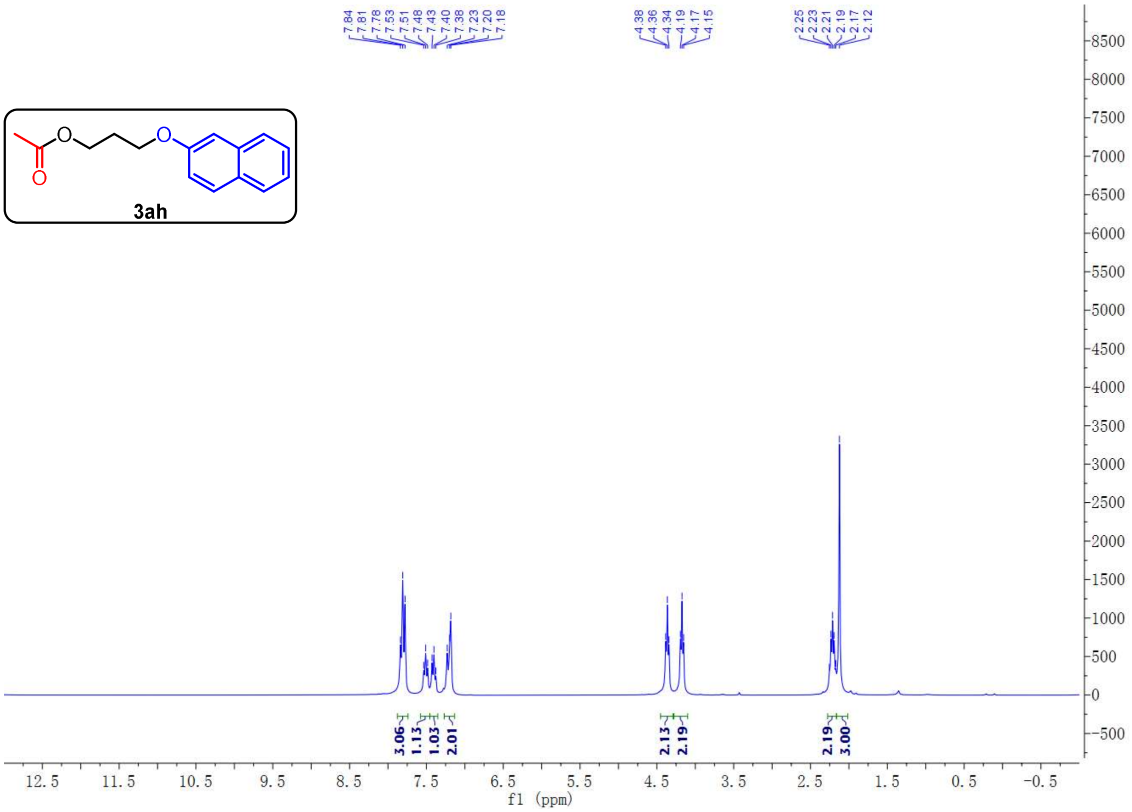
7.84 7.81 7.78 7.53 7.51 7.48 7.43 7.40 7.38 7.23 7.20 7.18
4.38 4.36 4.34 4.19 4.17 4.15
2.25 2.23 2.21 2.19 2.17 2.12
3ah
3.06 1.13 1.03 2.01
2.13 2.19
2.19 3.00
f1 (ppm)

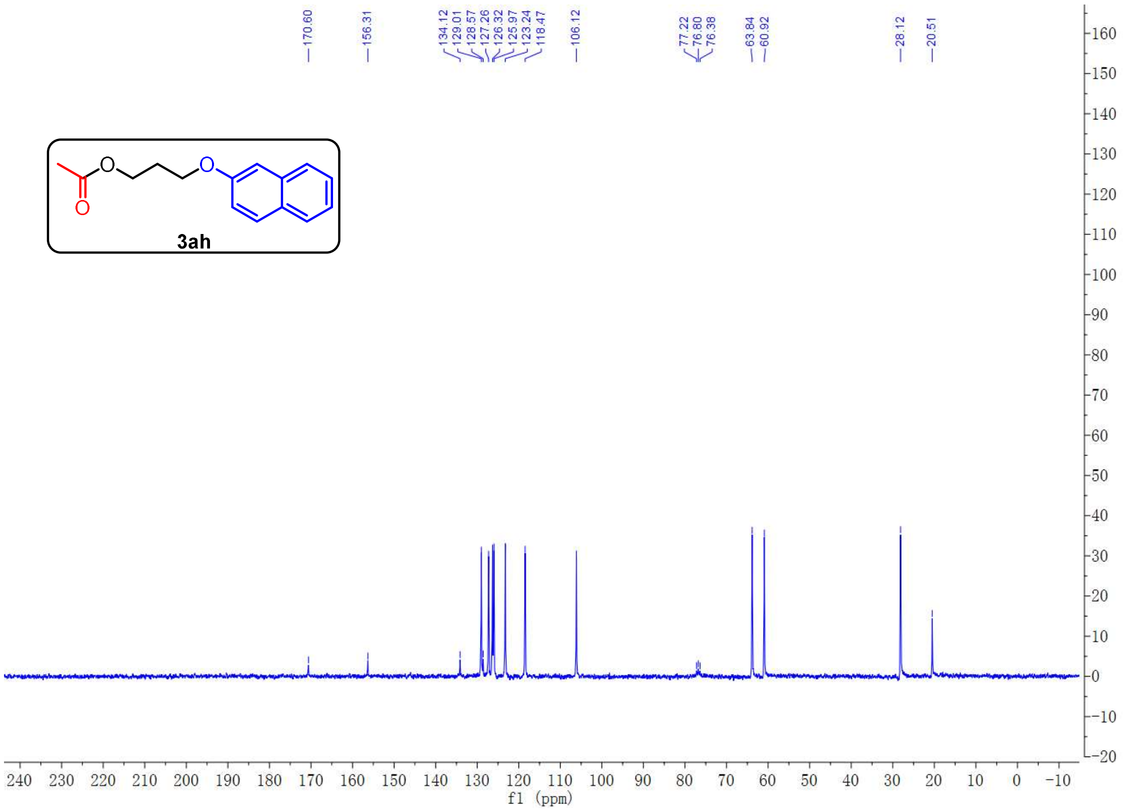
170.60
156.31
134.12 129.01 128.57 127.26 126.32 125.97 123.24 118.47
106.12
77.22 76.80 76.38
63.84 60.92
28.12
20.51
3ah
f1 (ppm)

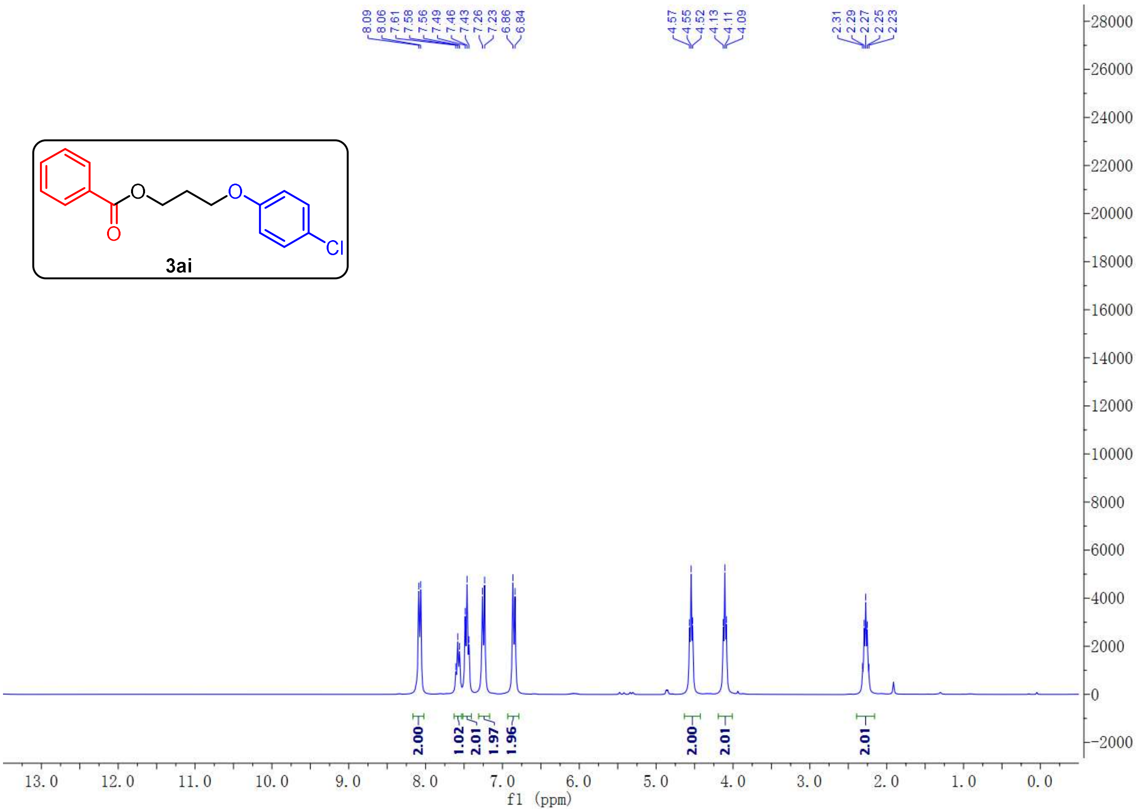
3ai
8.09 8.06 7.61 7.58 7.56 7.49 7.46 7.43 7.26 7.23 6.86 6.84
4.57 4.55 4.52 4.13 4.11 4.09
2.31 2.29 2.27 2.25 2.23
2.00 1.02 2.01 1.97 1.96 2.00 2.01 2.01
f1 (ppm)

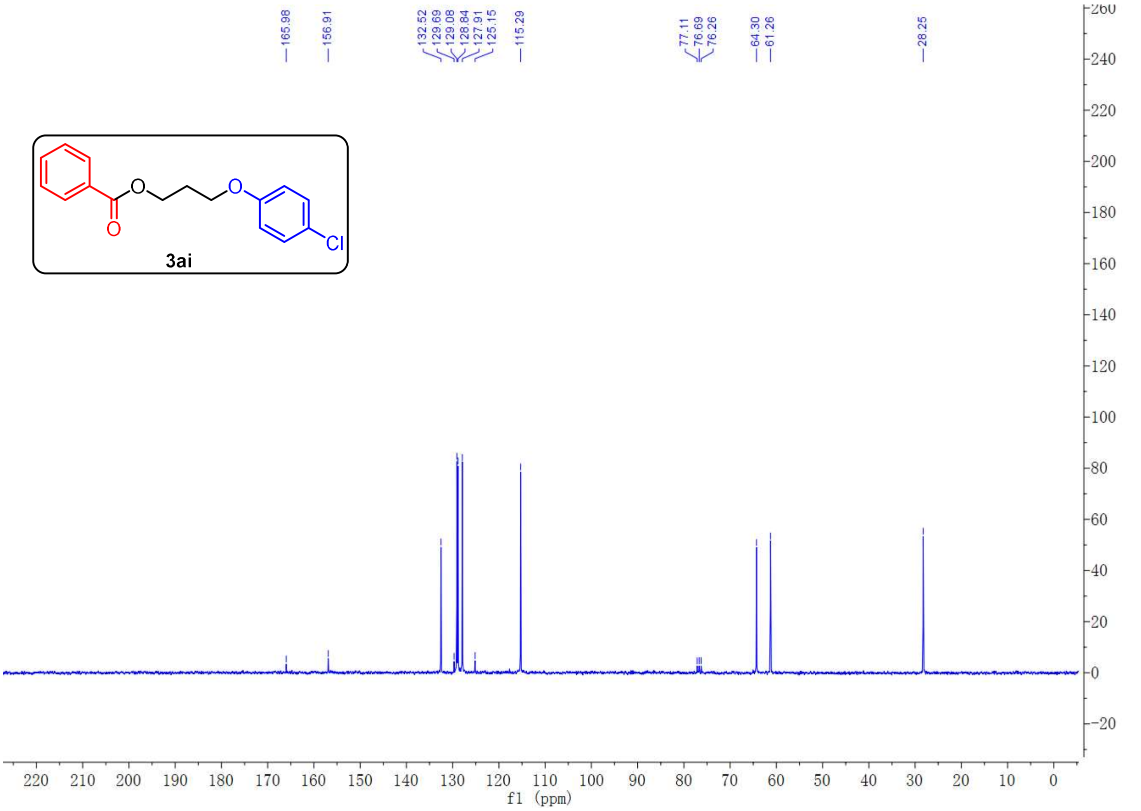
3ai
165.98
156.91
132.52 129.69 129.08 128.84 127.91 125.15
115.29
77.11 76.69 76.26
64.30 61.26
28.25
f1 (ppm)

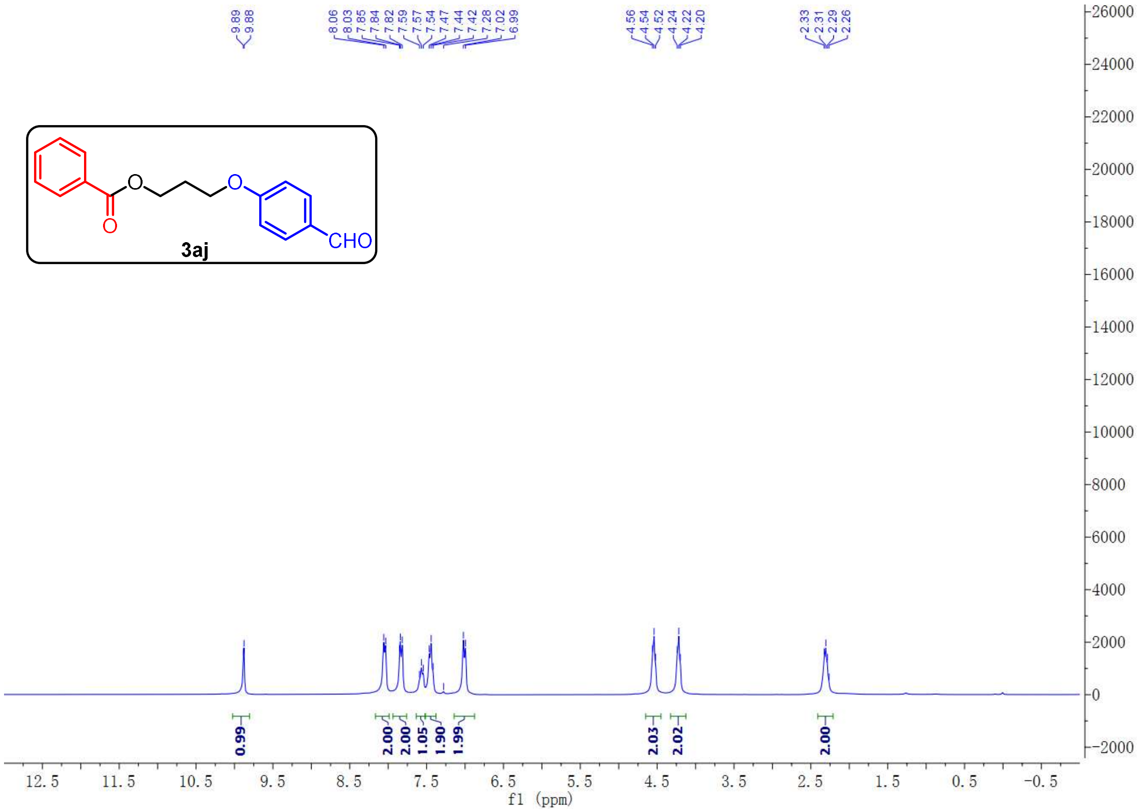
CHO
3aj
f1 (ppm)

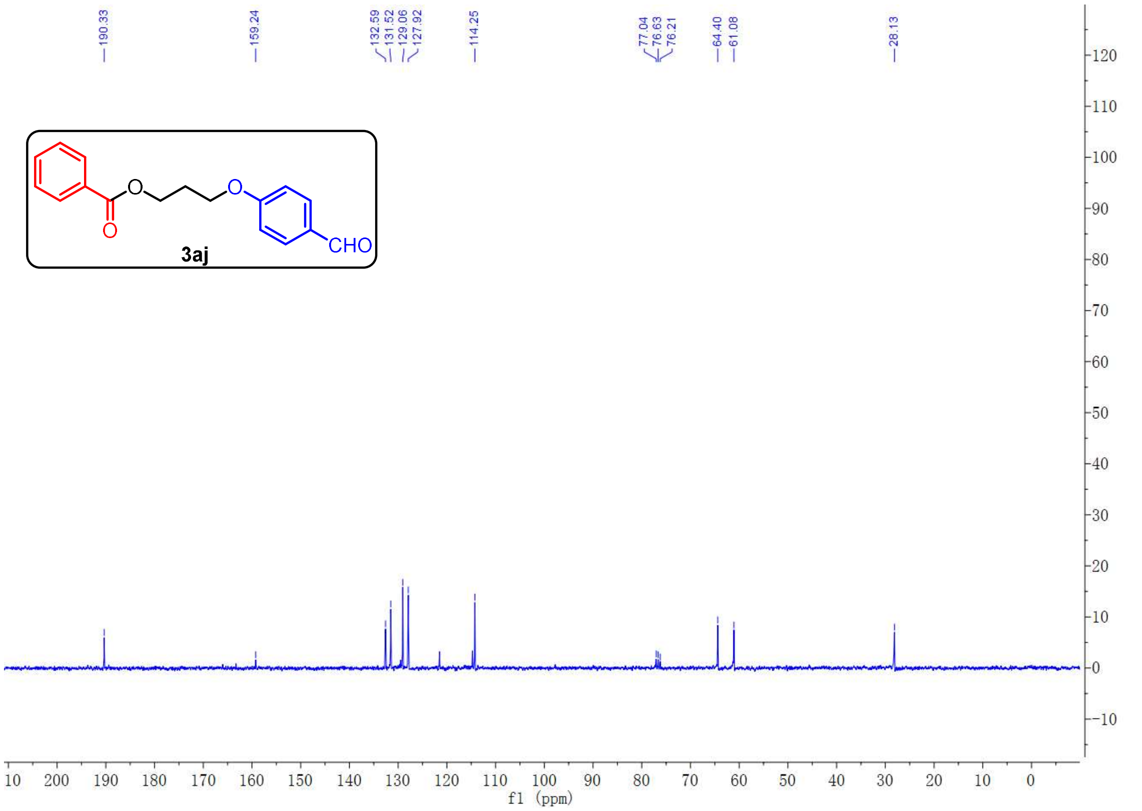
CHO
3aj
f1 (ppm)

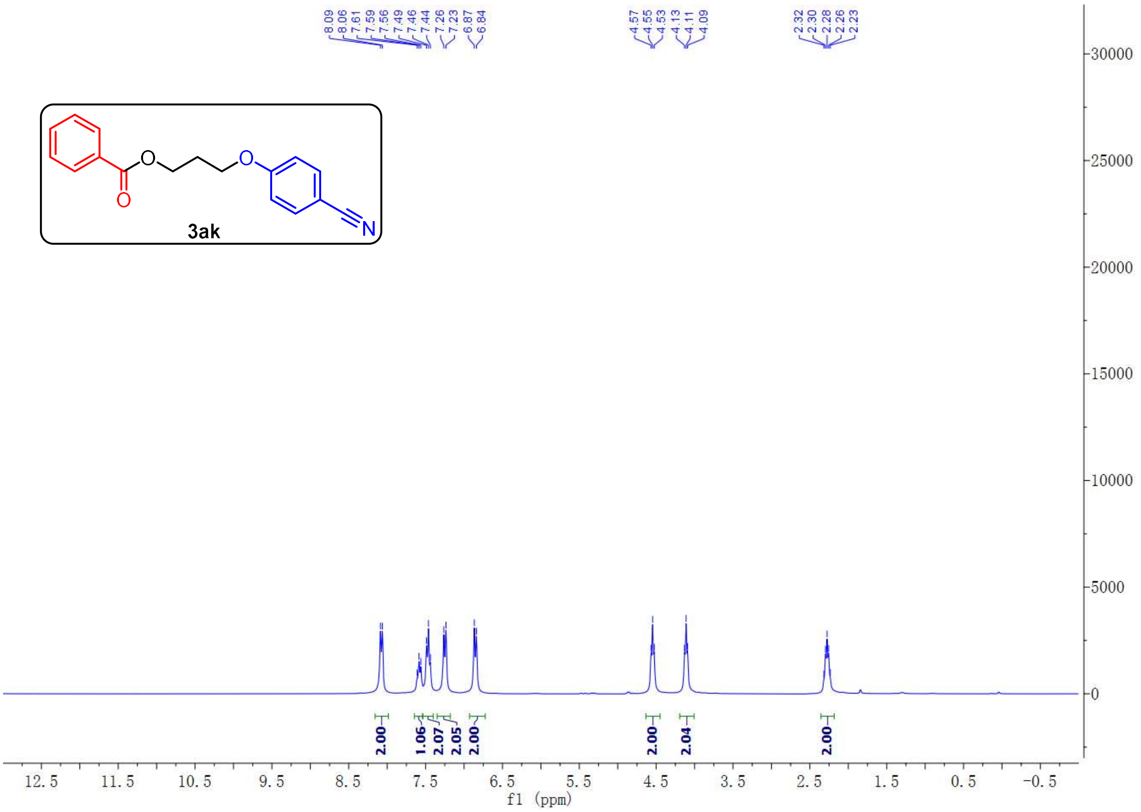
8.09
8.06
7.61
7.59
7.56
7.49
7.46
7.44
7.26
7.23
6.87
6.84
4.57
4.55
4.53
4.13
4.11
4.09
2.32
2.30
2.28
2.26
2.23
3ak
2.00
1.06
2.07
2.05
2.00
2.00
2.04
2.00
f1 (ppm)

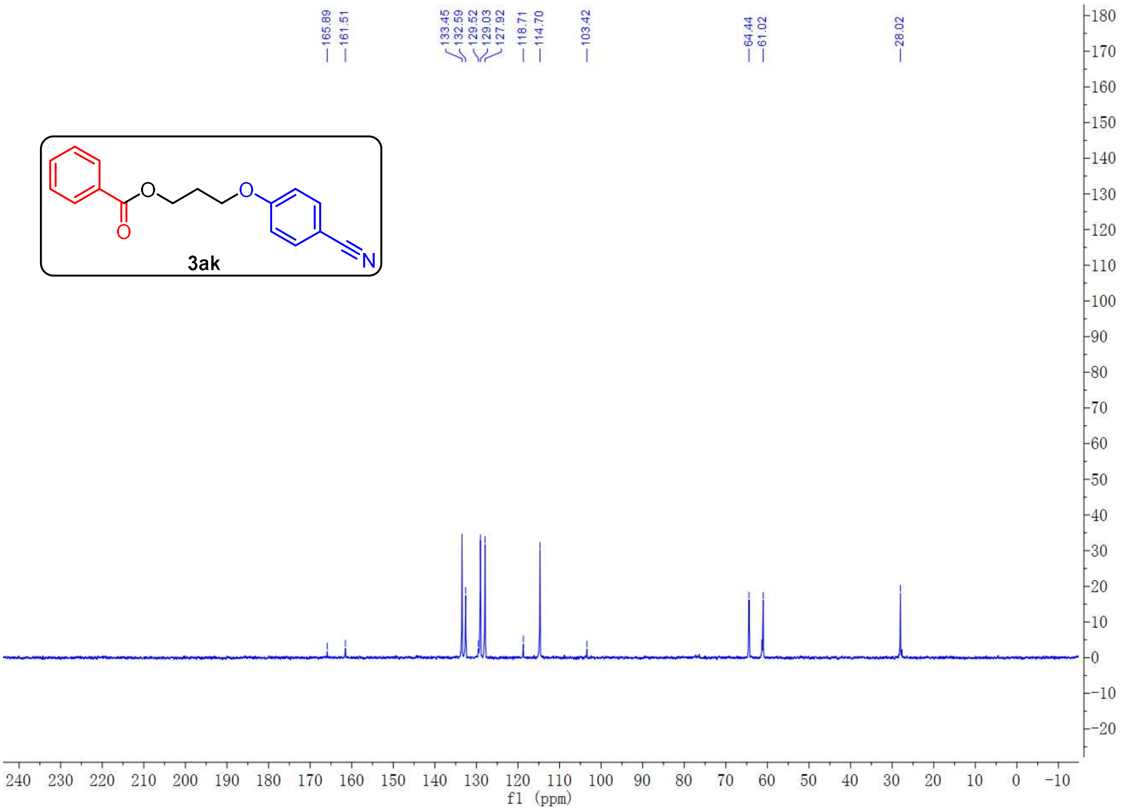
165.89
161.51
133.45
132.59
129.52
129.03
127.92
118.71
114.70
103.42
64.44
61.02
28.02
3ak
f1 (ppm)

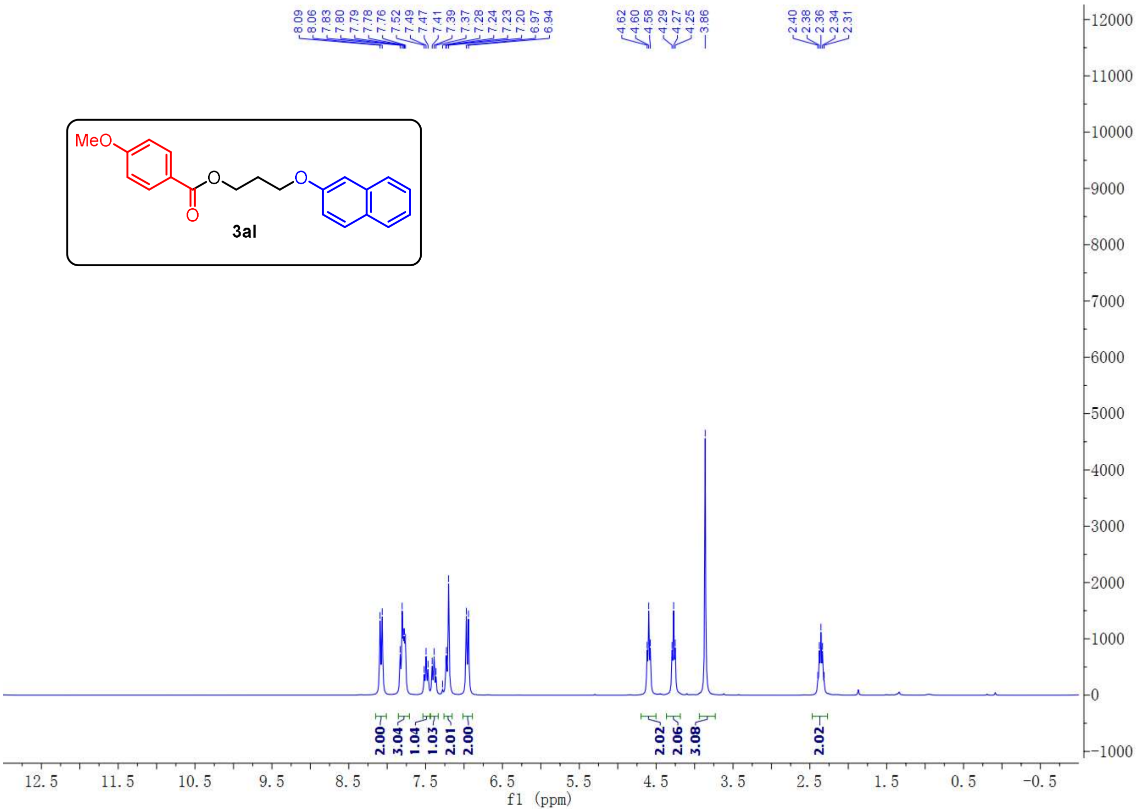
8.09
8.06
7.83
7.80
7.79
7.78
7.76
7.52
7.49
7.47
7.41
7.39
7.37
7.28
7.24
7.23
7.20
6.97
6.94
4.62
4.60
4.58
4.29
4.27
4.25
3.86
2.40
2.38
2.36
2.34
2.31
MeO
O
O
O
3al
2.00
3.04
1.04
1.03
2.01
2.00
2.02
2.06
3.08
2.02
f1 (ppm)

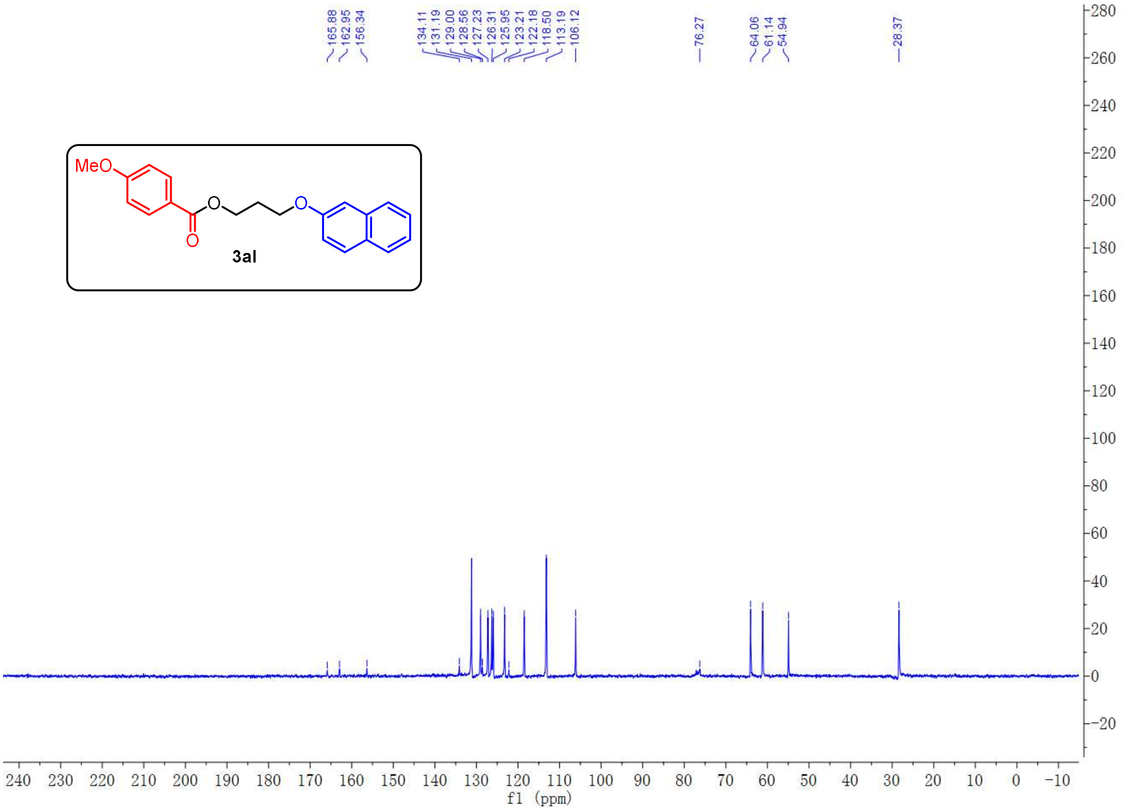
165.88
162.95
156.34
134.11
131.19
129.00
128.56
127.23
126.31
125.95
123.21
122.18
118.50
113.19
106.12
76.27
64.06
61.14
54.94
28.37
MeO
O
O
O
3al
f1 (ppm)

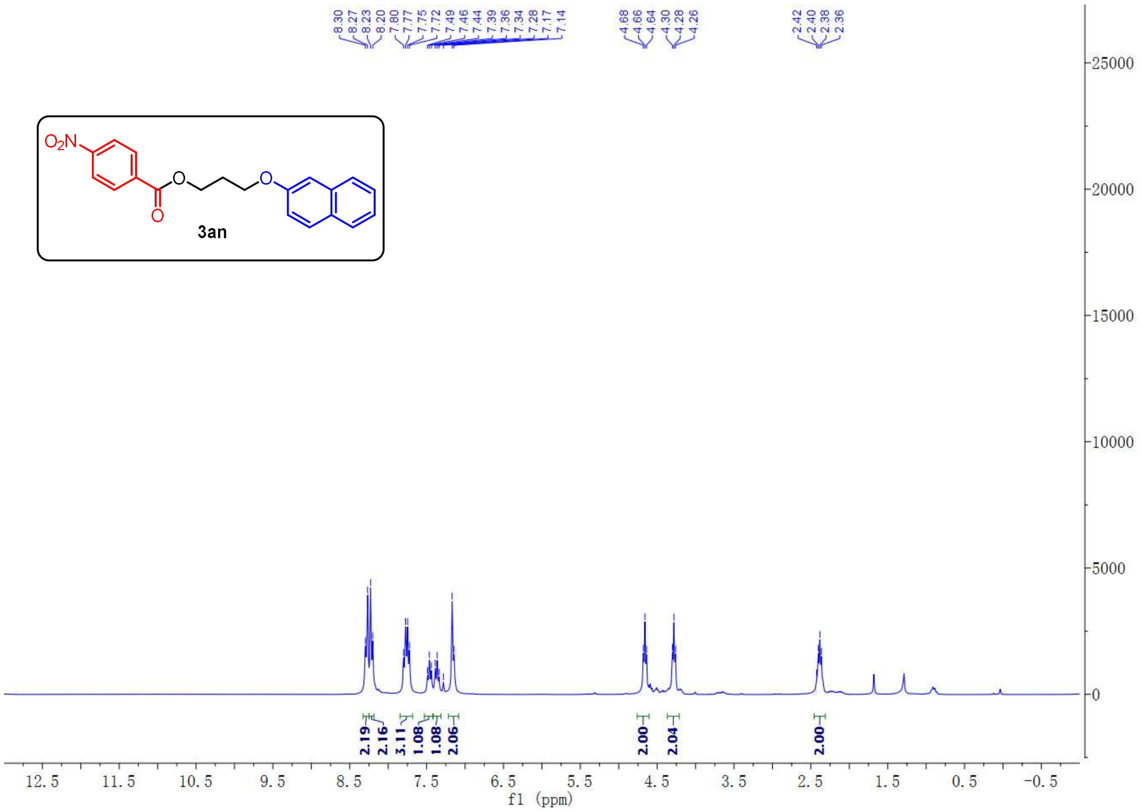
8.30 8.27 8.23 8.20 7.80 7.77 7.75 7.72 7.49 7.46 7.44 7.39 7.36 7.34 7.28 7.17 7.14
4.68 4.66 4.64 4.30 4.28 4.26
2.42 2.40 2.38 2.36
O_2N
3an
2.19 2.16 3.11 1.08 1.08 2.06 2.00 2.04 2.00
f1 (ppm)

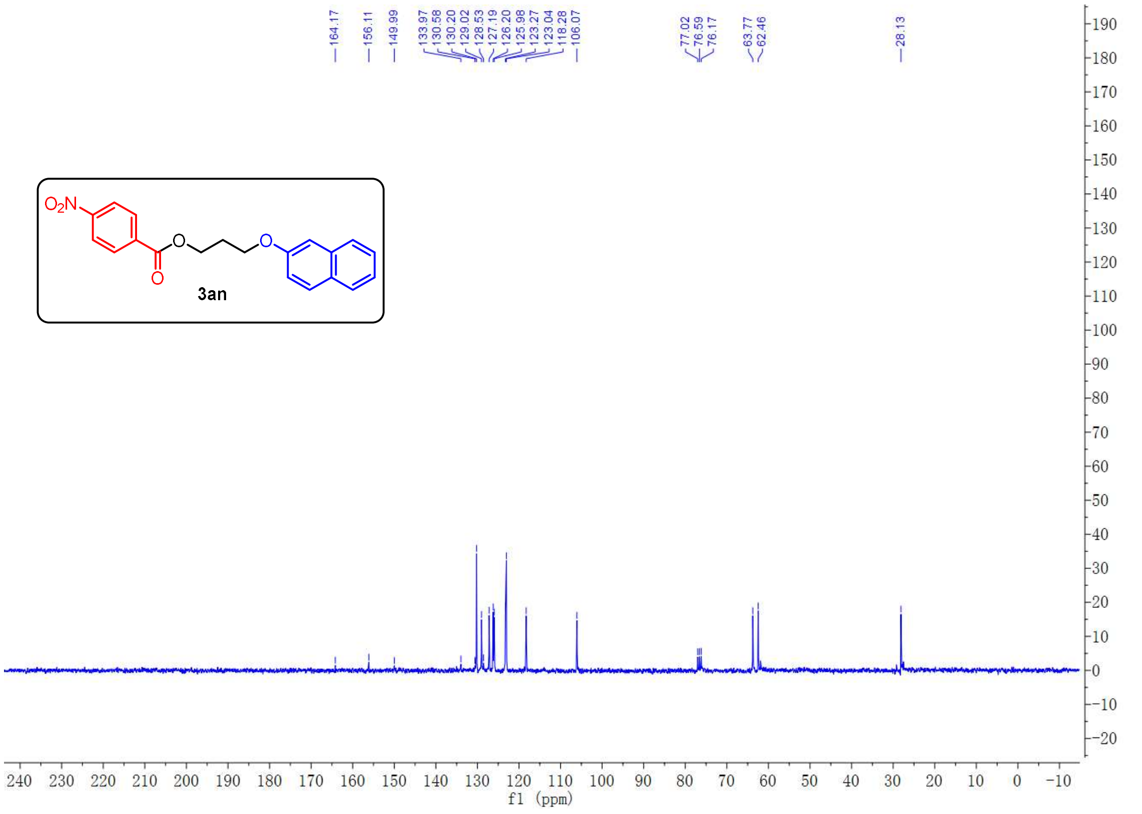
164.17
156.11
149.99
133.97 130.58 130.20 129.02 128.53 127.19 126.20 125.98 123.27 123.04 118.28
106.07
77.02 76.59 76.17
63.77 62.46
28.13
O_2N
3an
f1 (ppm)

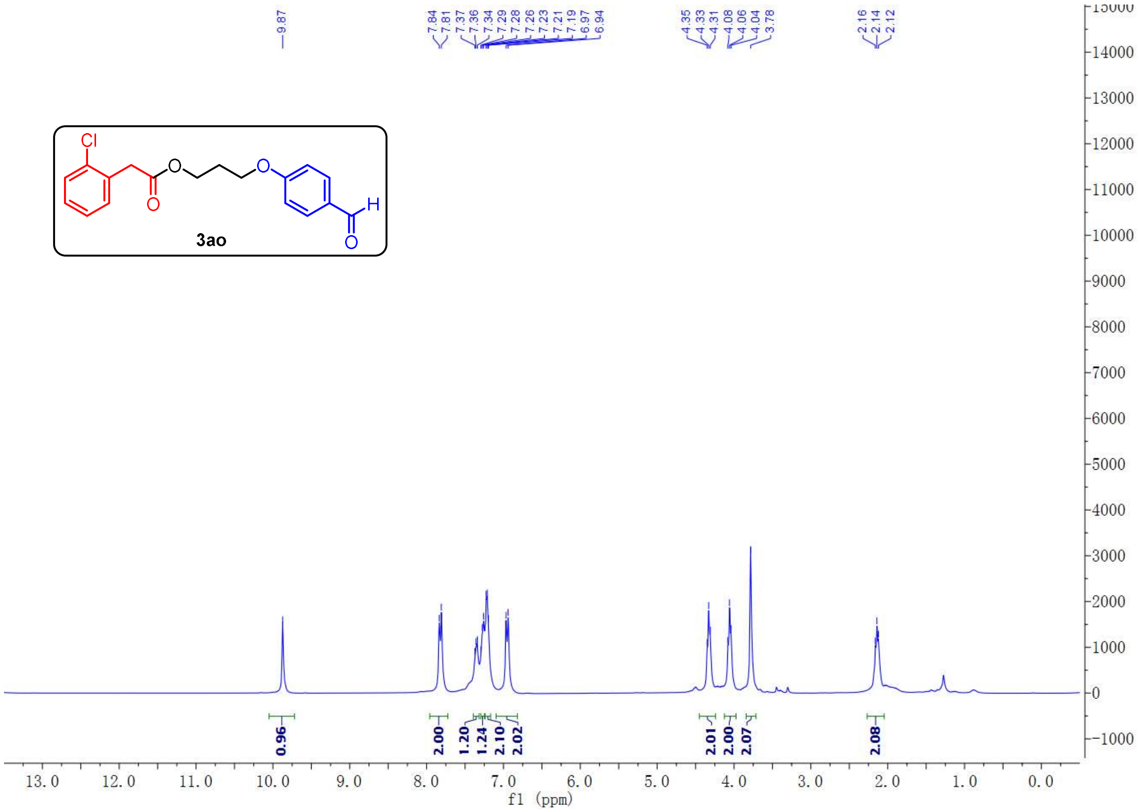
9.87
7.84 7.81 7.37 7.36 7.34 7.29 7.28 7.26 7.23 7.21 7.19 6.97 6.94
4.35 4.33 4.31 4.08 4.06 4.04 3.78
2.16 2.14 2.12
Cl
O
O
O
H
O
3ao
0.96
2.00
1.20
1.24
2.10
2.02
2.01
2.00
2.07
2.08
f1 (ppm)

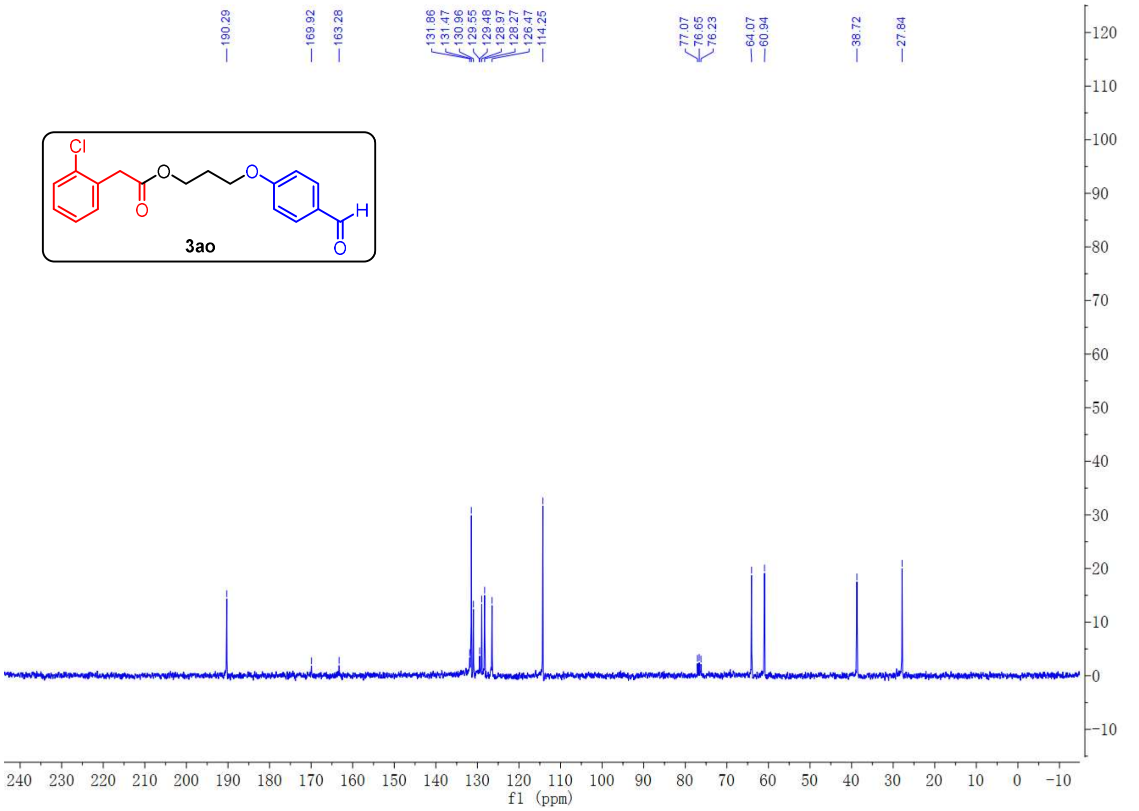
190.29
169.92
163.28
131.86 131.47 130.96 129.55 129.48 128.97 128.27 126.47
114.25
77.07 76.65 76.23
64.07
60.94
38.72
27.84
Cl
O
O
O
H
O
3ao
f1 (ppm)

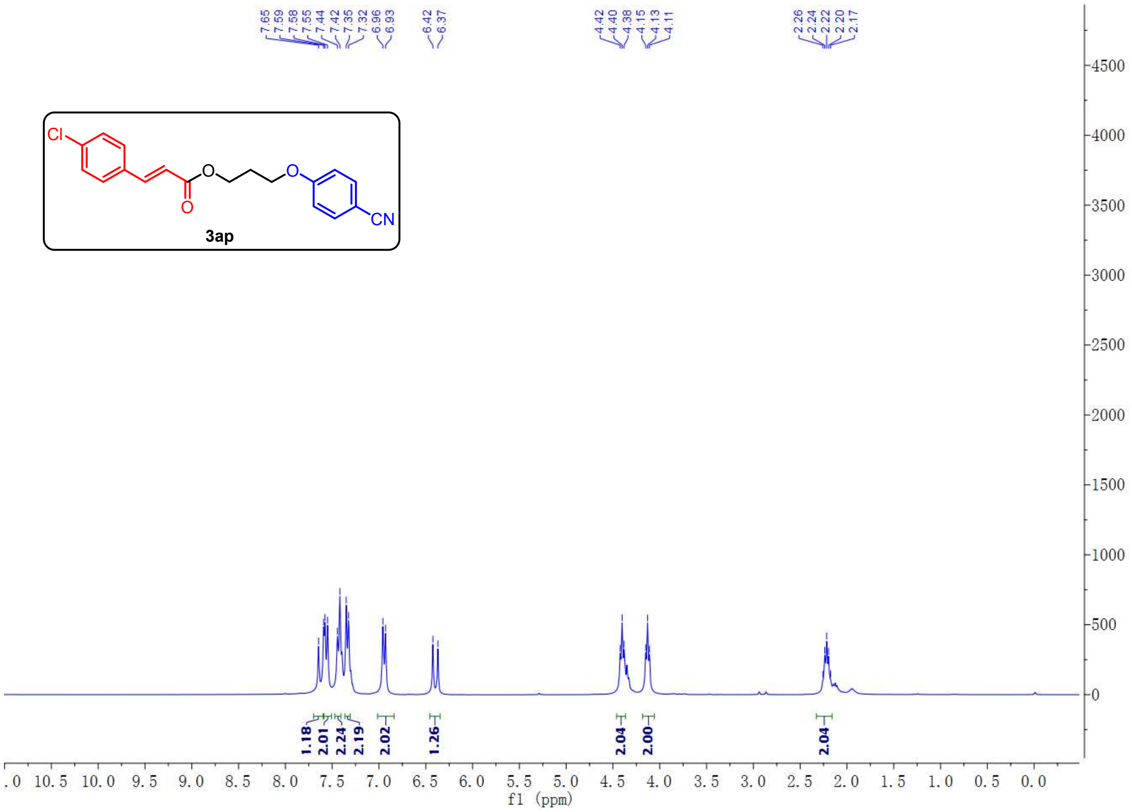
7.65
7.59
7.58
7.55
7.44
7.42
7.35
7.32
6.96
6.93
6.42
6.37
4.42
4.40
4.38
4.15
4.13
4.11
2.26
2.24
2.22
2.20
2.17
Cl
O
O
O
CN
3ap
1.18
2.01
2.24
2.19
2.02
1.26
2.04
2.00
2.04
f1 (ppm)

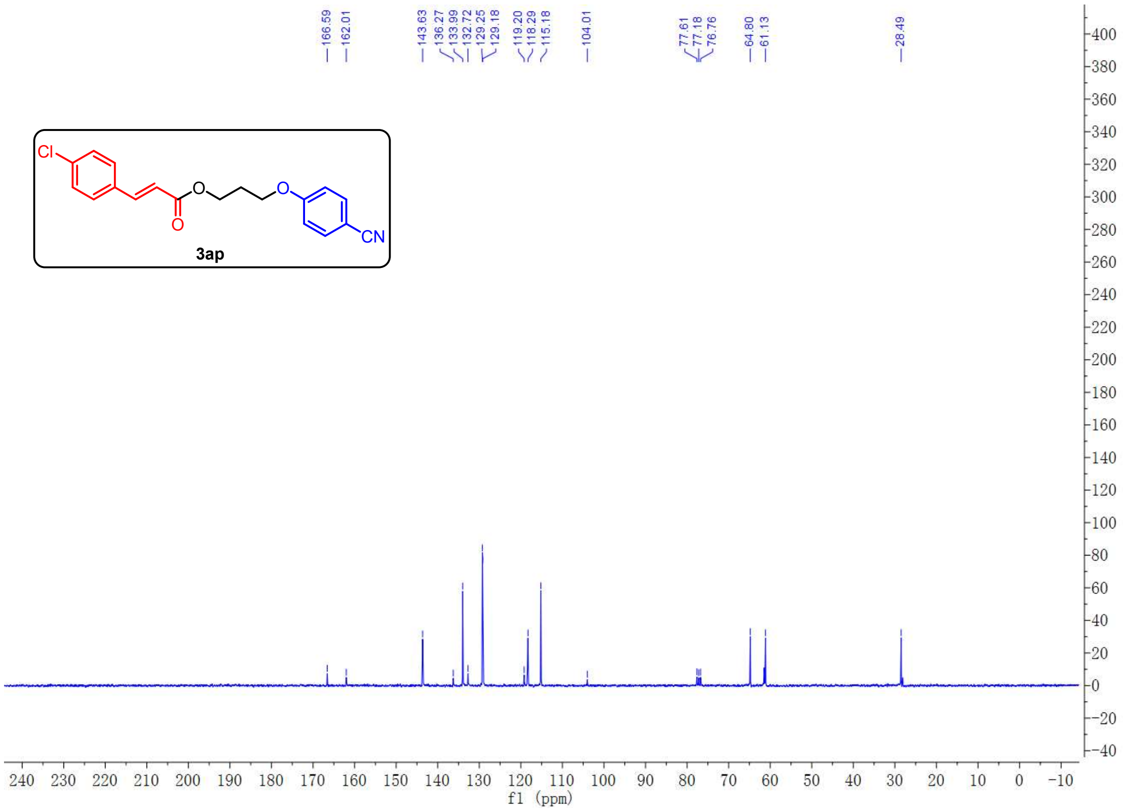
166.59
162.01
143.63
136.27
133.99
132.72
129.25
129.18
119.20
118.29
115.18
104.01
77.61
77.18
76.76
64.80
61.13
28.49
Cl
O
O
O
CN
3ap
f1 (ppm)

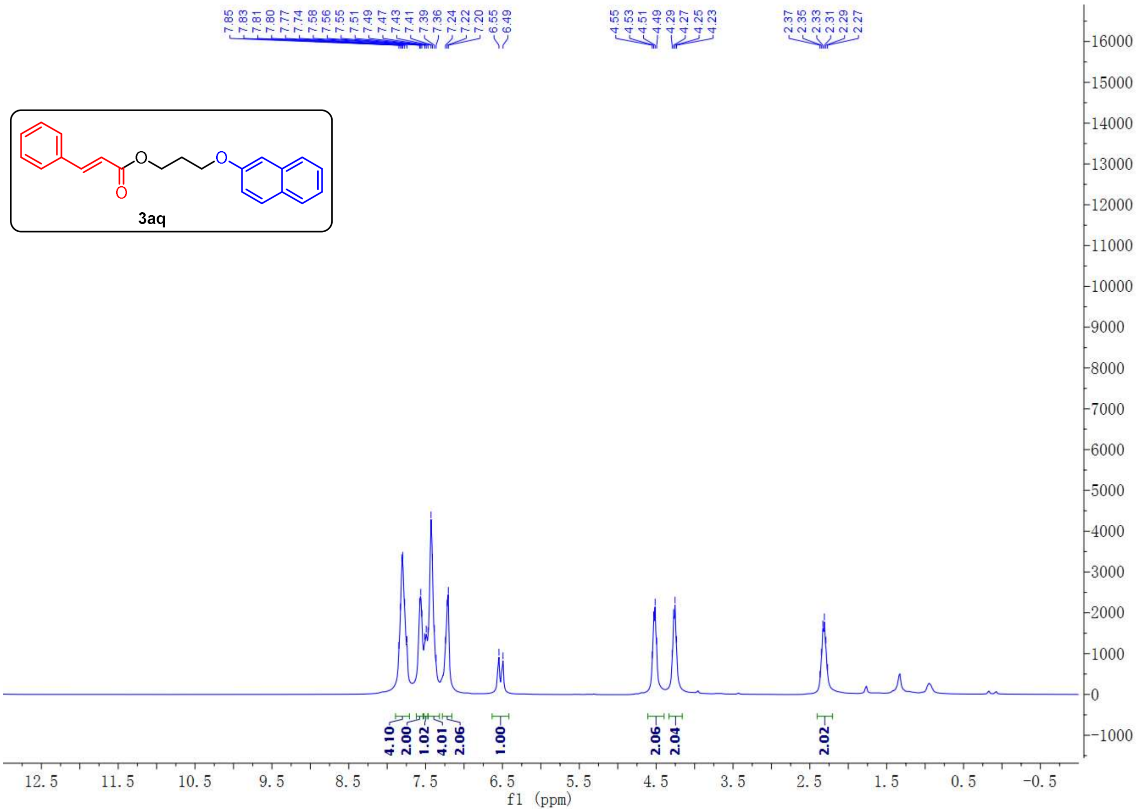
3aq
f1 (ppm)

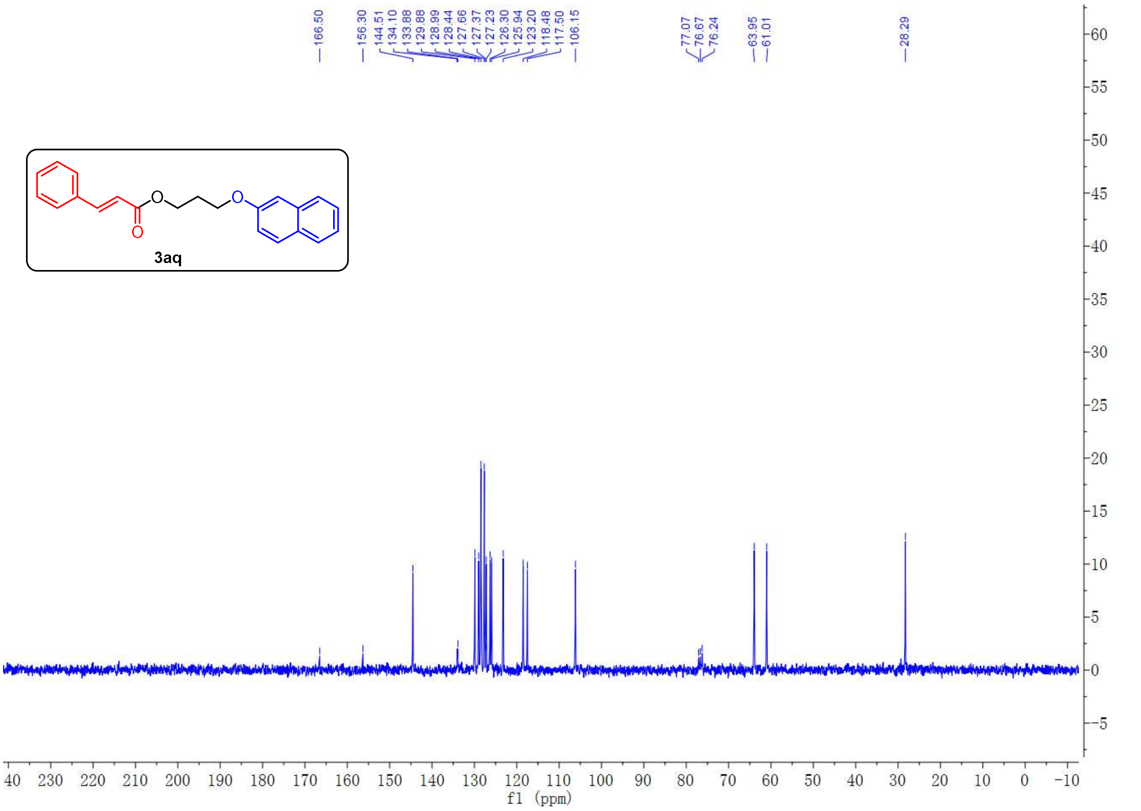
3aq
f1 (ppm)

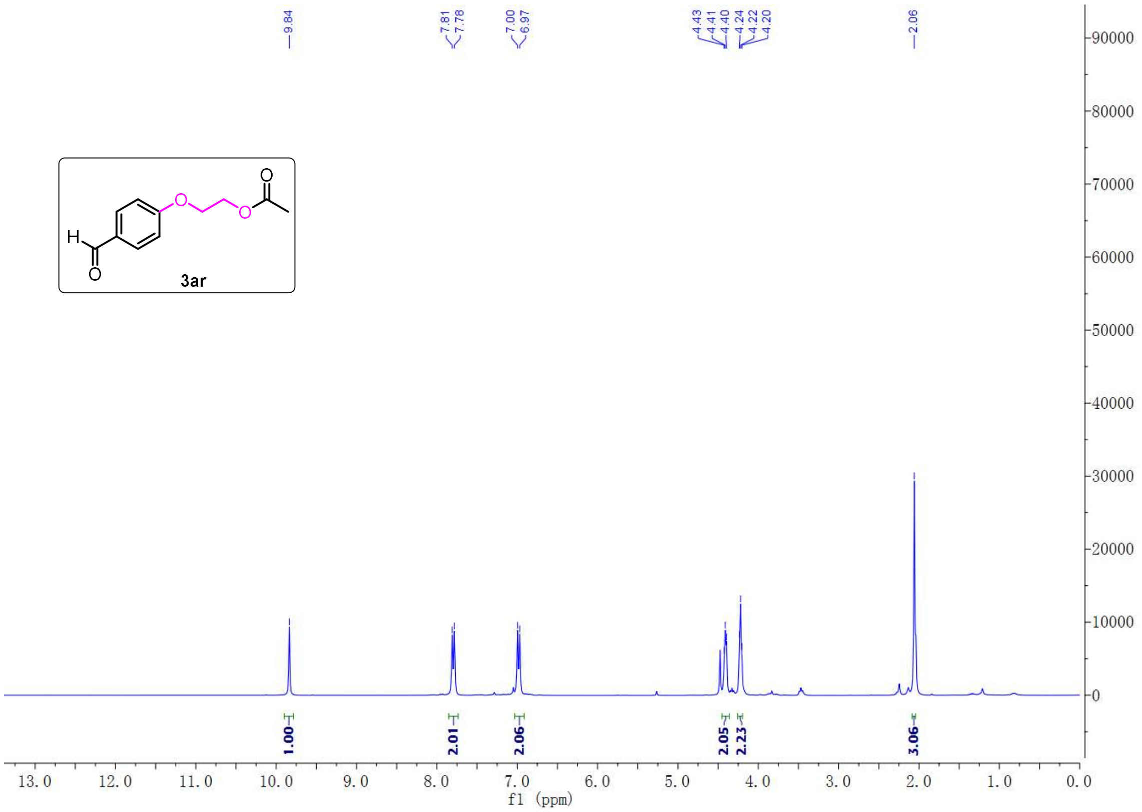
9.84
7.81
7.78
7.00
6.97
4.43
4.41
4.40
4.24
4.22
4.20
2.06
3ar
1.00
2.01
2.06
2.05
2.23
3.06
f1 (ppm)

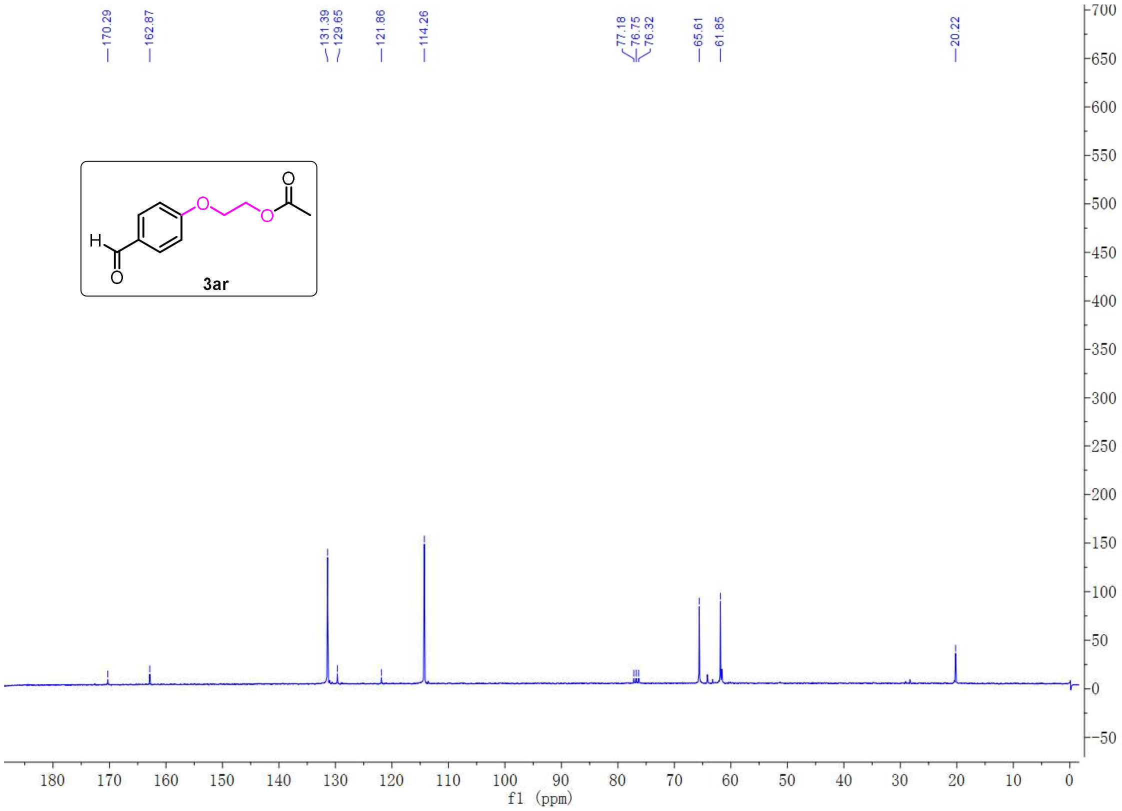
170.29
162.87
131.39
129.65
121.86
114.26
77.18
76.75
76.32
65.61
61.85
20.22
3ar
f1 (ppm)

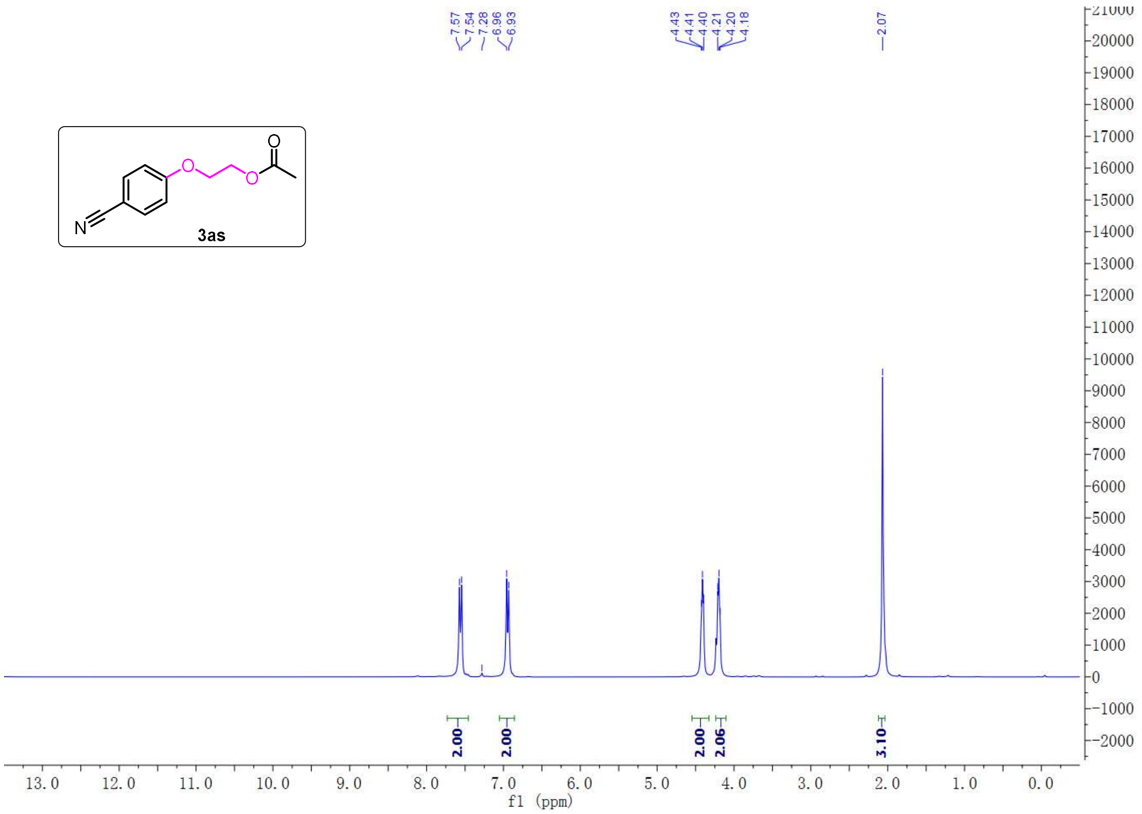
3as
7.57
7.54
7.28
6.96
6.93
4.43
4.41
4.40
4.21
4.20
4.18
2.07
2.00
2.00
2.00
2.06
3.10
f1 (ppm)

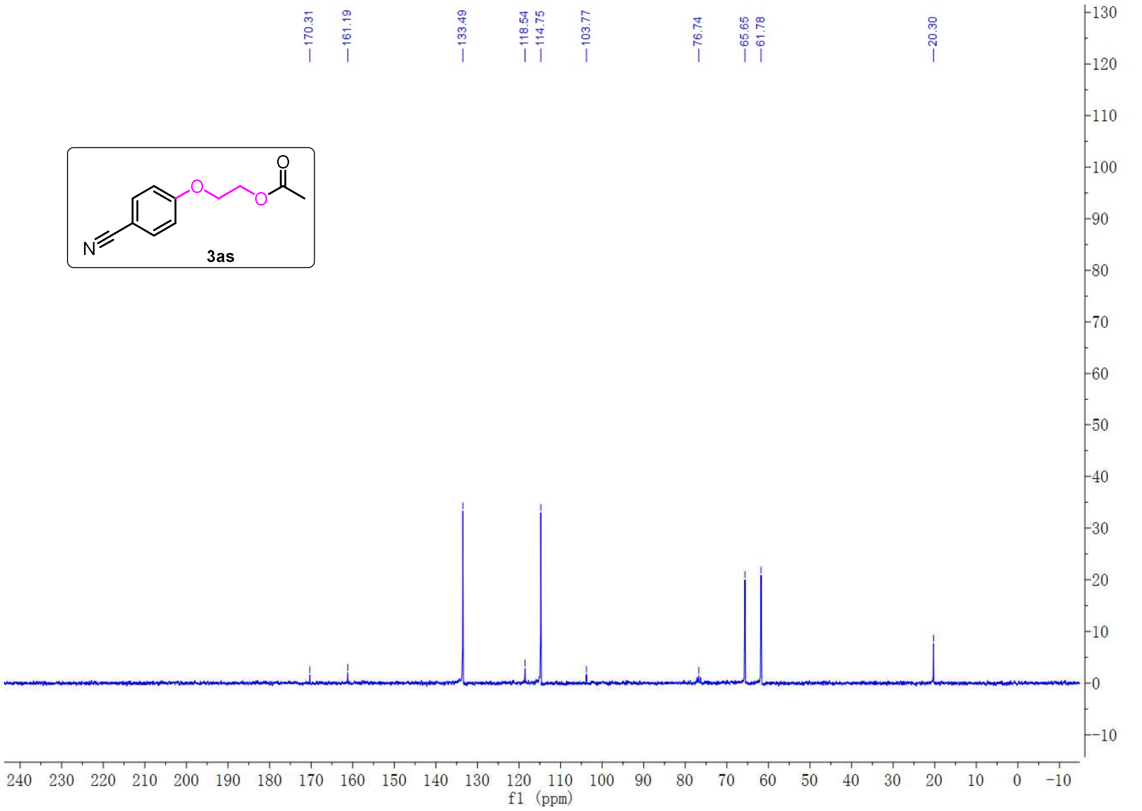
3as
170.31
161.19
133.49
118.54
114.75
103.77
76.74
65.65
61.78
20.30
f1 (ppm)

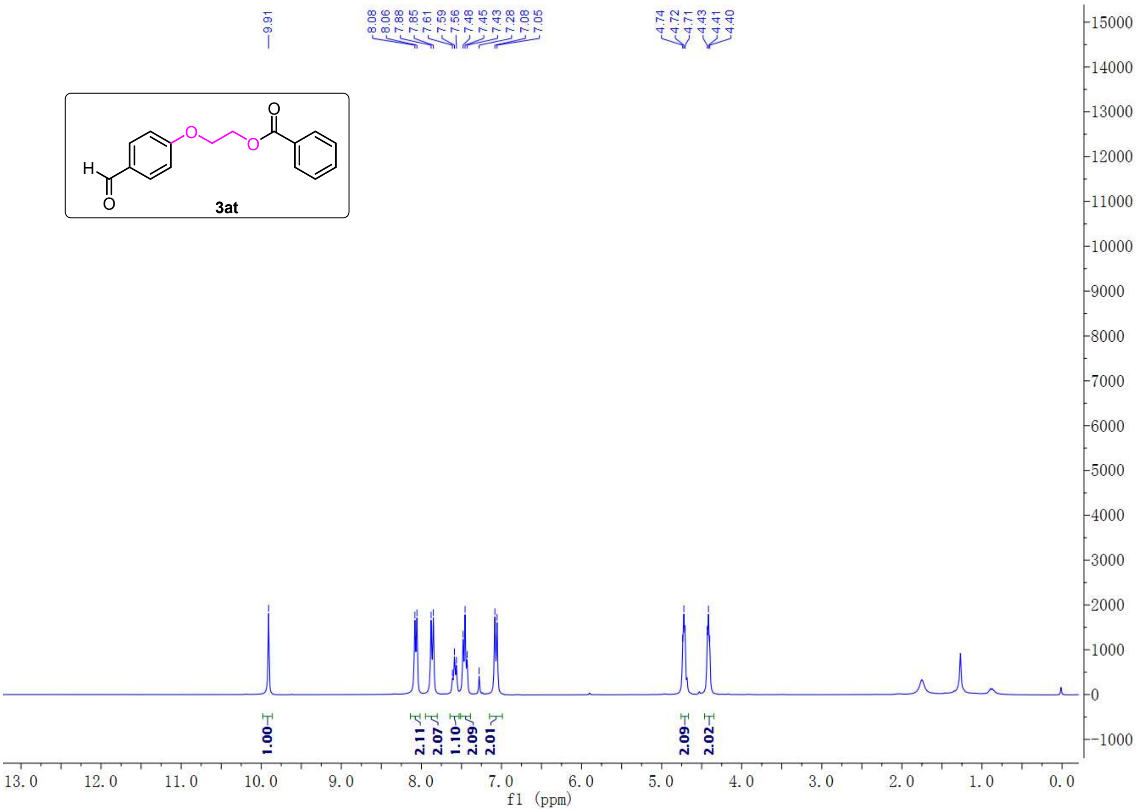

9.91
8.08 8.06 7.88 7.85 7.61 7.59 7.56 7.48 7.45 7.43 7.28 7.08 7.05
4.74 4.72 4.71 4.43 4.41 4.40
H
O
O
O
O
3at
1.00
2.11 2.07 1.10 2.09 2.01
2.09 2.02
13.0 12.0 11.0 10.0 9.0 8.0 7.0 6.0 5.0 4.0 3.0 2.0 1.0 0.0
f1 (ppm)
15000 14000 13000 12000 11000 10000 9000 8000 7000 6000 5000 4000 3000 2000 1000 0 -1000

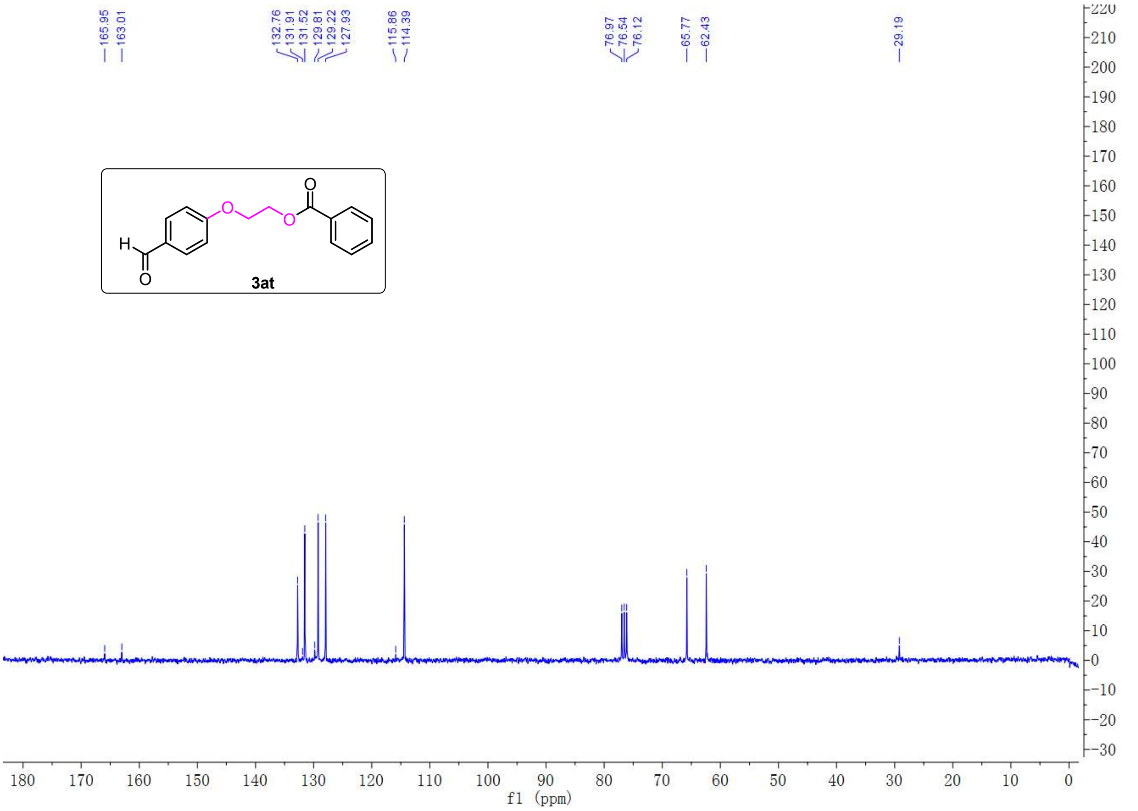

165.95 163.01
132.76 131.91 131.52 129.81 129.22 127.93
115.86 114.39
76.97 76.54 76.12
65.77 62.43
29.19
H
O
O
O
O
3at
180 170 160 150 140 130 120 110 100 90 80 70 60 50 40 30 20 10 0
f1 (ppm)
220 210 200 190 180 170 160 150 140 130 120 110 100 90 80 70 60 50 40 30 20 10 0 -10 -20 -30

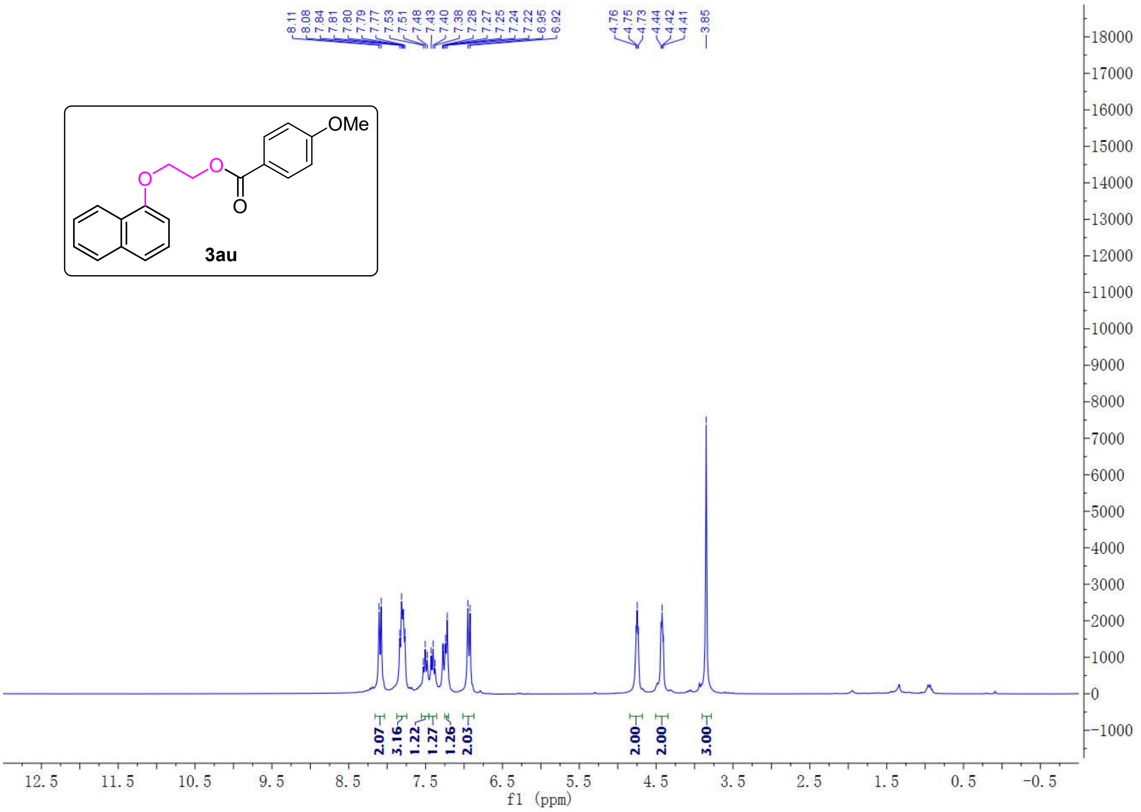
8.11 8.08 7.84 7.81 7.80 7.79 7.77 7.53 7.51 7.48 7.43 7.40 7.38 7.28 7.27 7.25 7.24 7.22 6.95 6.92
4.76 4.75 4.73 4.44 4.42 4.41
3.85
OMe
O
O
O
3au
2.07 3.16 1.22 1.27 1.26 2.03
2.00 2.00
3.00
f1 (ppm)

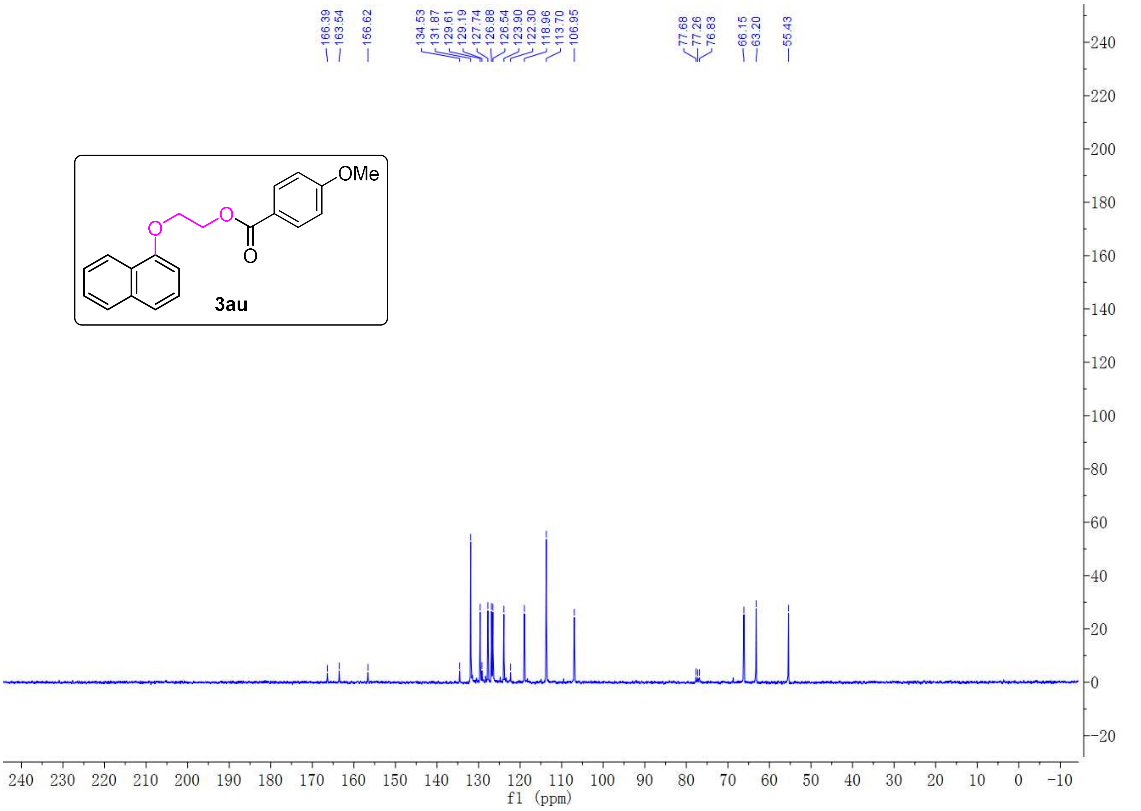
166.39 163.54
156.62
134.53 131.87 129.61 129.19 127.74 126.88 126.54 123.90 122.30 118.96 113.70 106.95
77.68 77.26 76.83
66.15 63.20
55.43
OMe
O
O
O
3au
f1 (ppm)

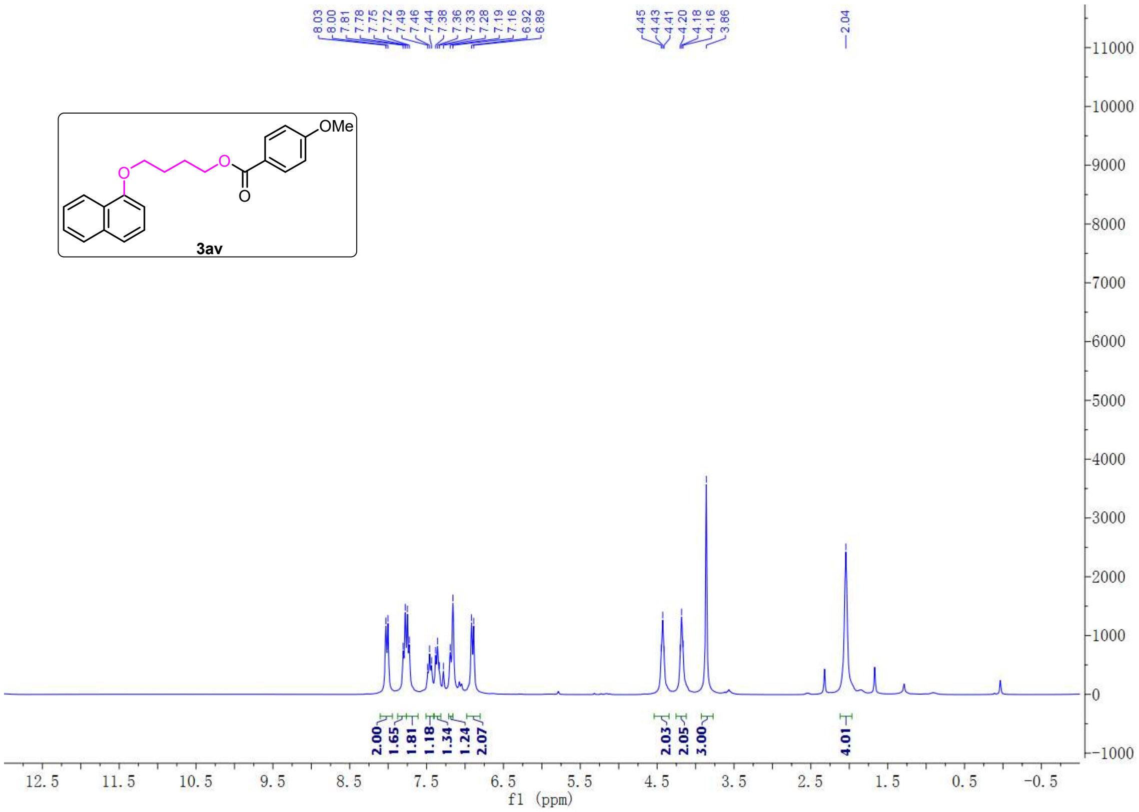
8.03
8.00
7.81
7.78
7.75
7.72
7.49
7.46
7.44
7.38
7.36
7.33
7.28
7.19
7.16
6.92
6.89
4.45
4.43
4.41
4.20
4.18
4.16
3.86
2.04
OMe
O
O
O
3av
2.00
1.65
1.81
1.18
1.34
1.24
2.07
2.03
2.05
3.00
4.01
f1 (ppm)

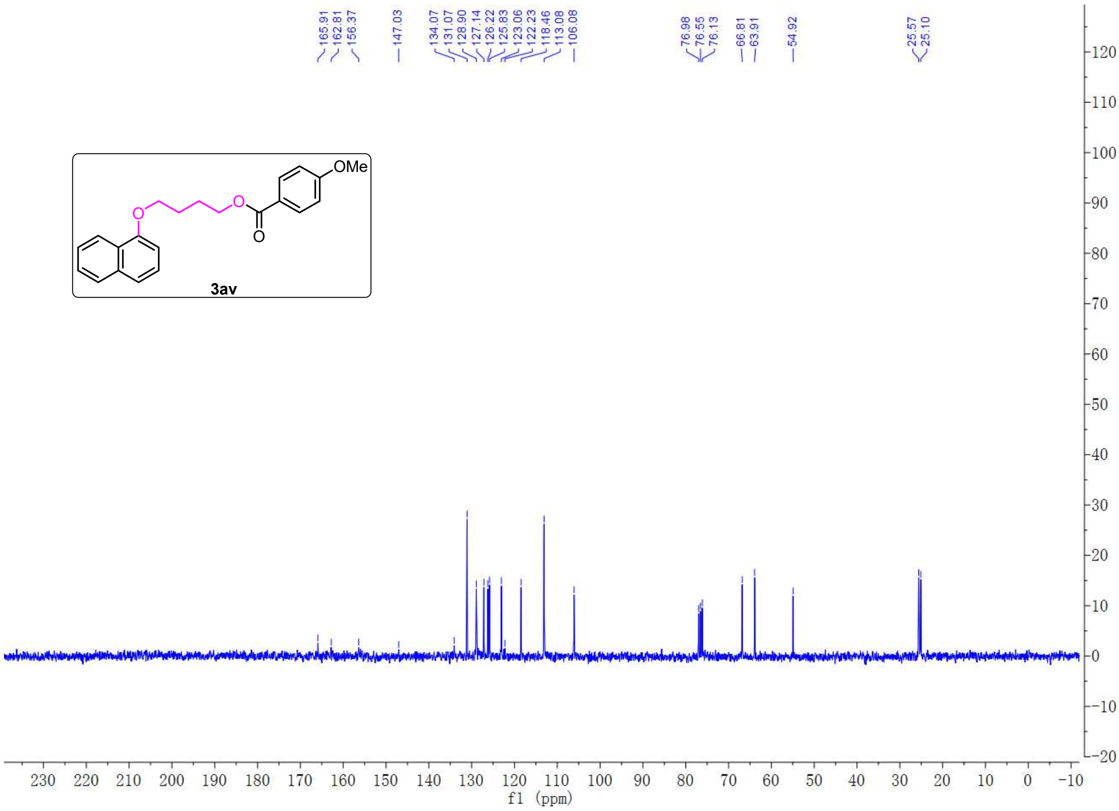
165.91
162.81
156.37
147.03
134.07
131.07
128.90
127.14
126.22
125.63
123.06
122.23
118.46
113.08
106.08
76.98
76.55
76.13
66.81
63.91
54.92
25.57
25.10
OMe
O
O
O
3av
f1 (ppm)

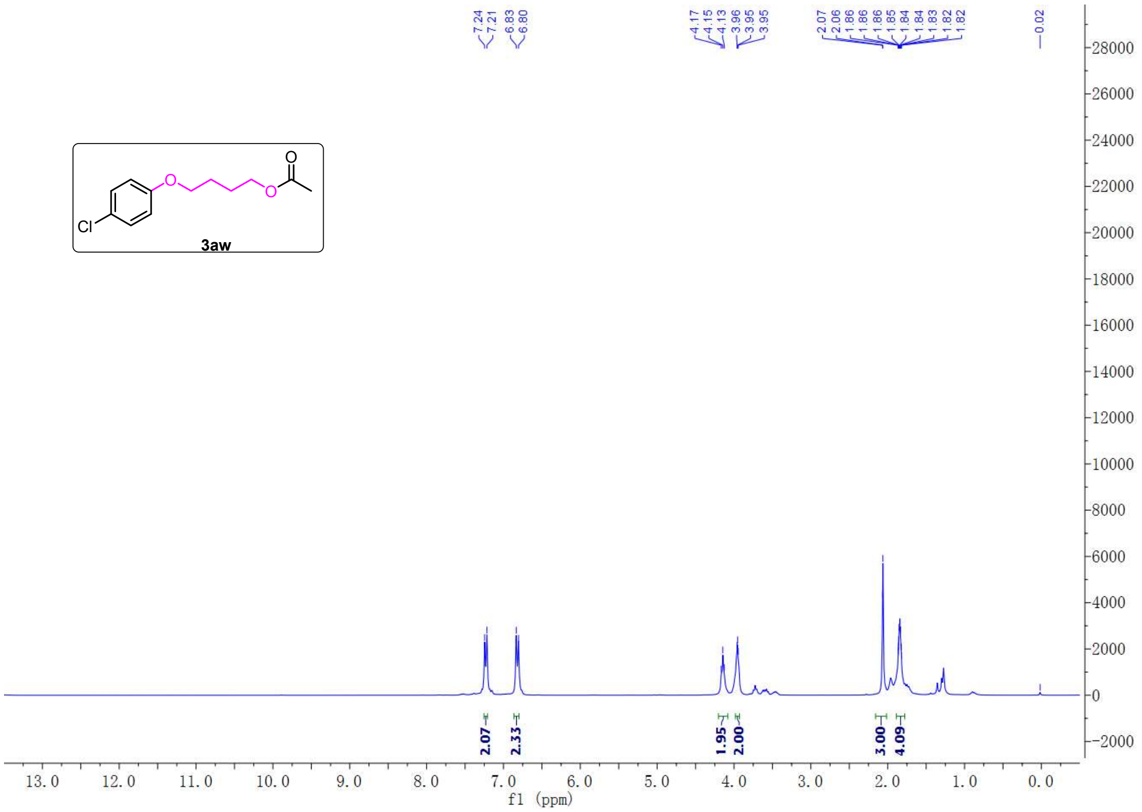

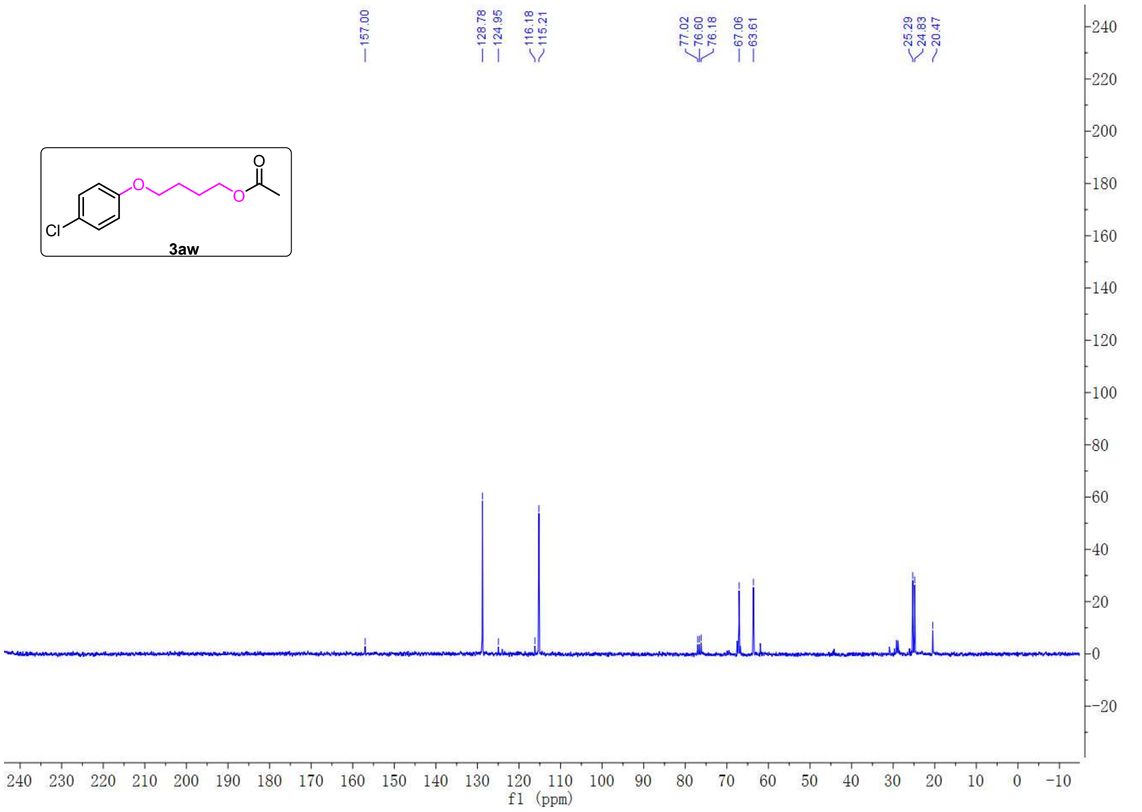

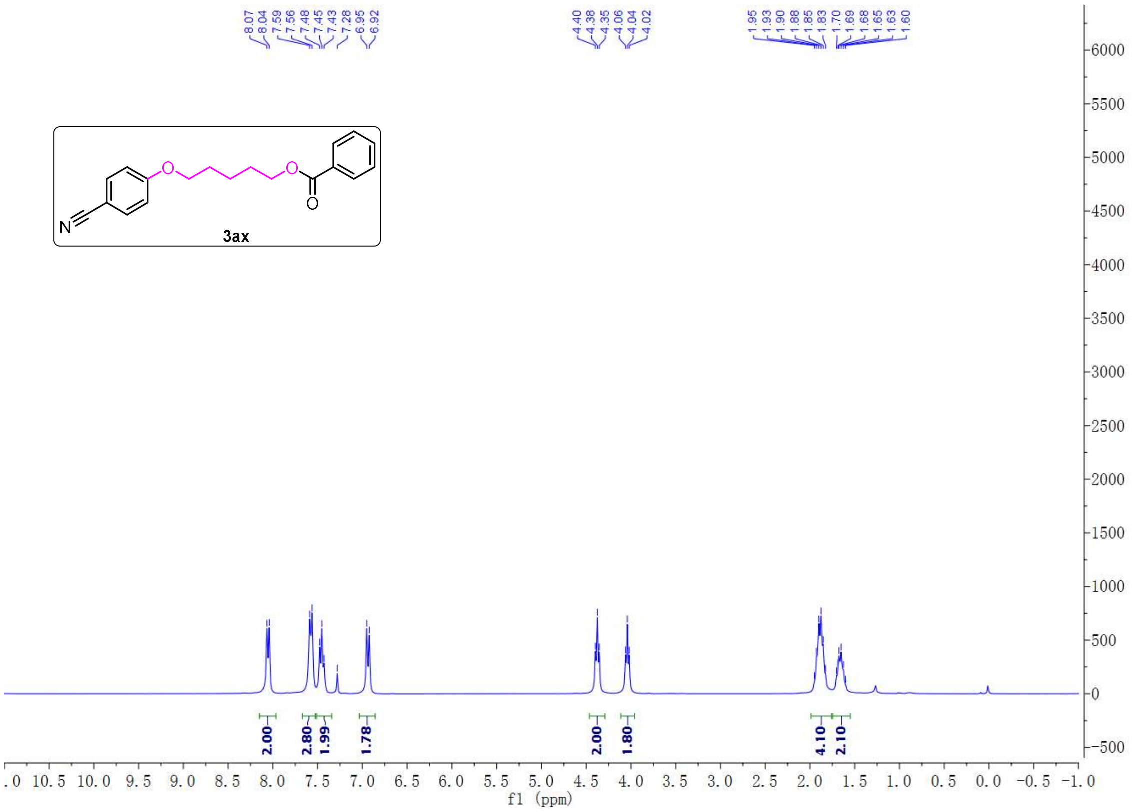
8.07
8.04
7.59
7.56
7.48
7.45
7.43
7.28
6.95
6.92
4.40
4.38
4.35
4.06
4.04
4.02
1.95
1.93
1.90
1.88
1.85
1.83
1.70
1.69
1.68
1.65
1.63
1.60
3ax
2.00
2.80
1.99
1.78
2.00
1.80
4.10
2.10
f1 (ppm)

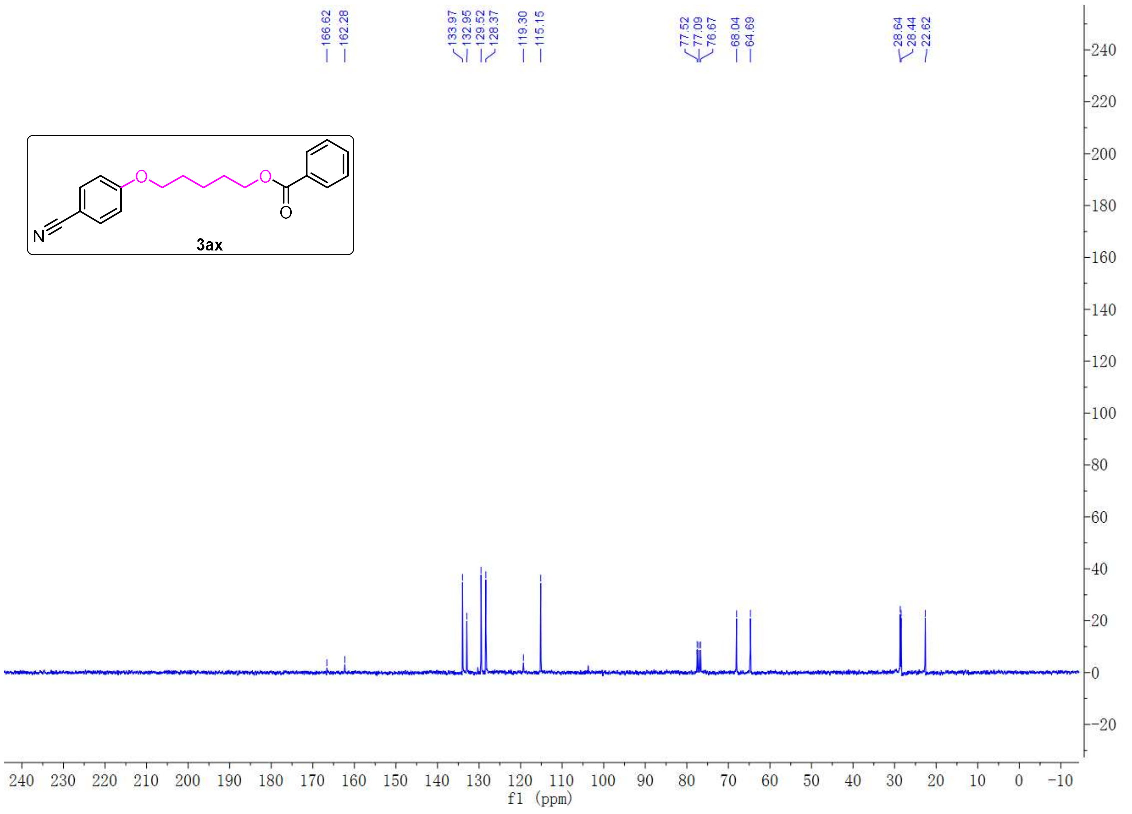
166.62
162.28
133.97
132.95
129.52
128.37
119.30
115.15
77.52
77.09
76.67
68.04
64.69
28.64
28.44
22.62
3ax
f1 (ppm)

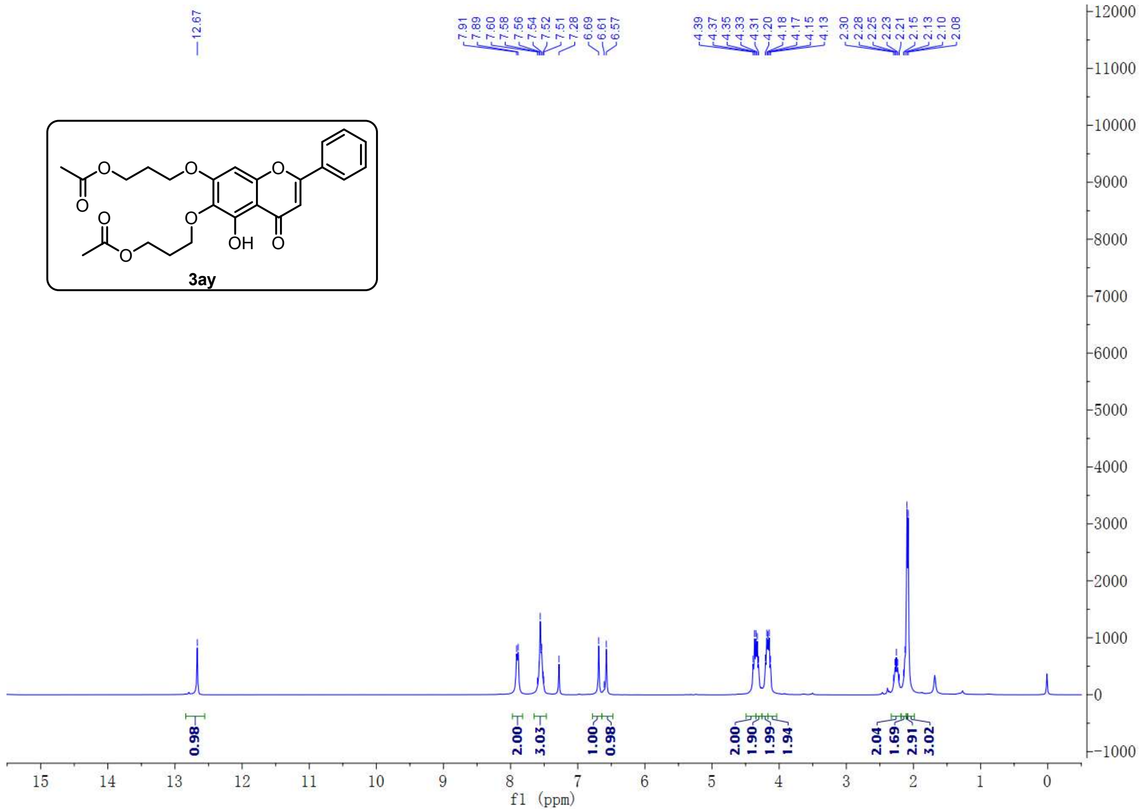
3ay
f1 (ppm)

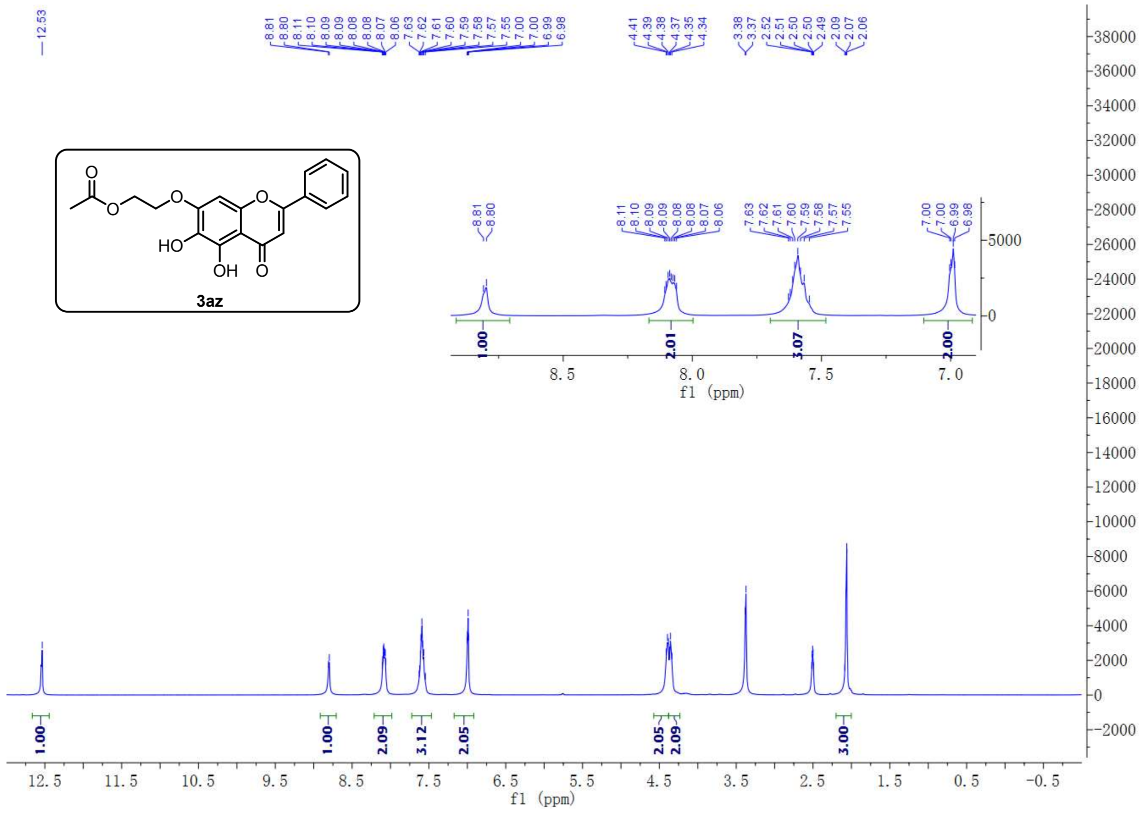
3az
f1 (ppm)

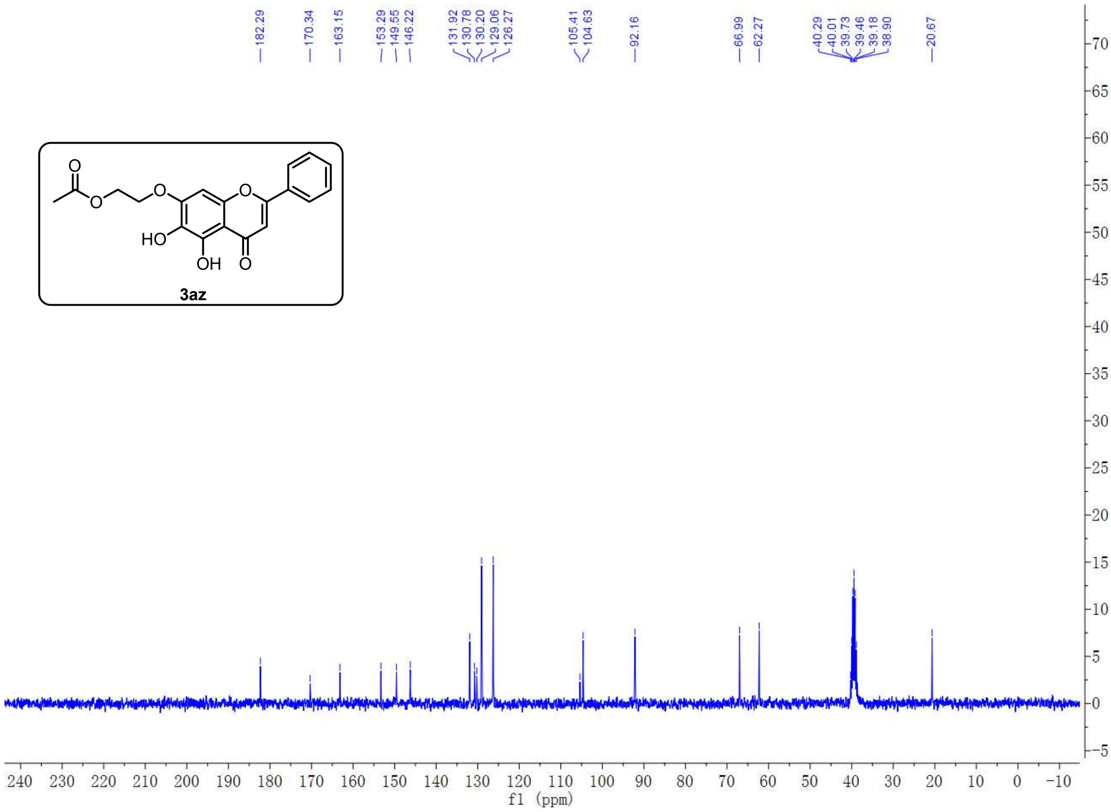

182.29
170.34
163.15
153.29
149.55
146.22
131.92
130.78
130.20
129.06
126.27
105.41
104.63
92.16
66.99
62.27
40.29
40.01
39.73
39.46
39.18
38.90
20.67
HO
OH
3az
f1 (ppm)

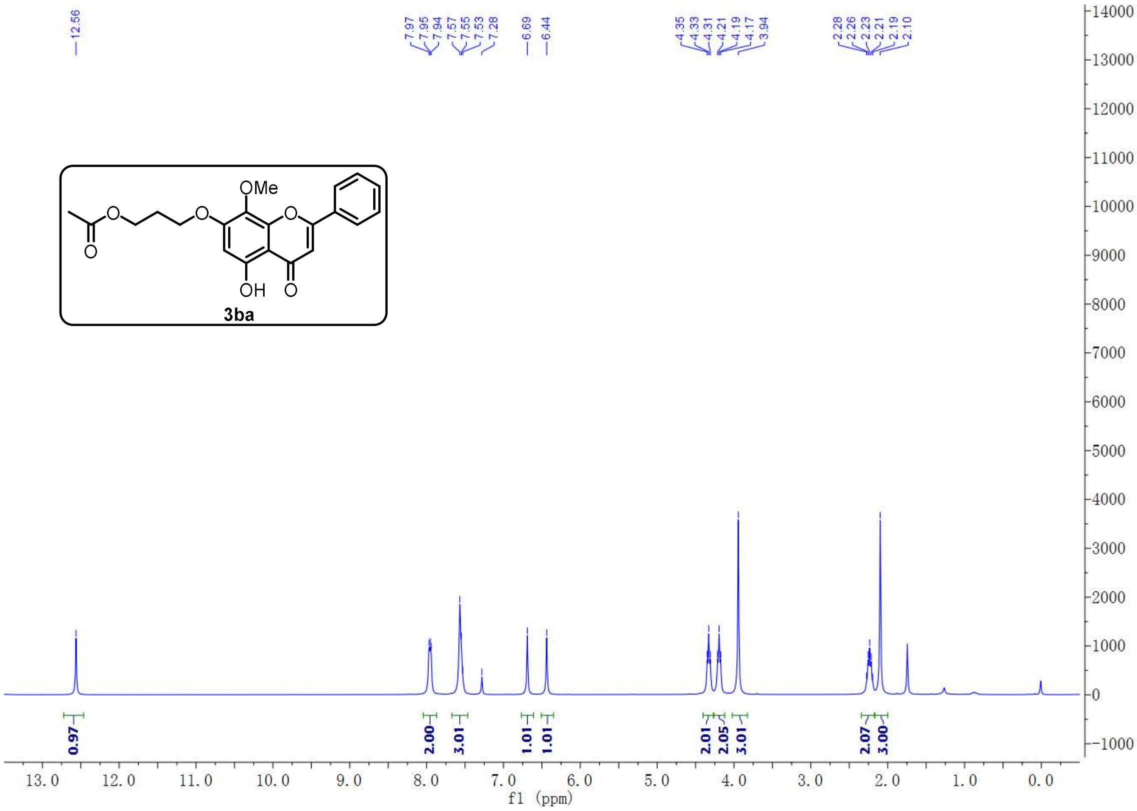

12.56
7.97
7.95
7.94
7.57
7.55
7.53
7.28
6.69
6.44
4.35
4.33
4.31
4.21
4.19
4.17
3.94
2.28
2.26
2.23
2.21
2.19
2.10
OMe
OH
3ba
0.97
2.00
3.01
1.01
1.01
2.01
2.05
3.01
2.07
3.00
f1 (ppm)

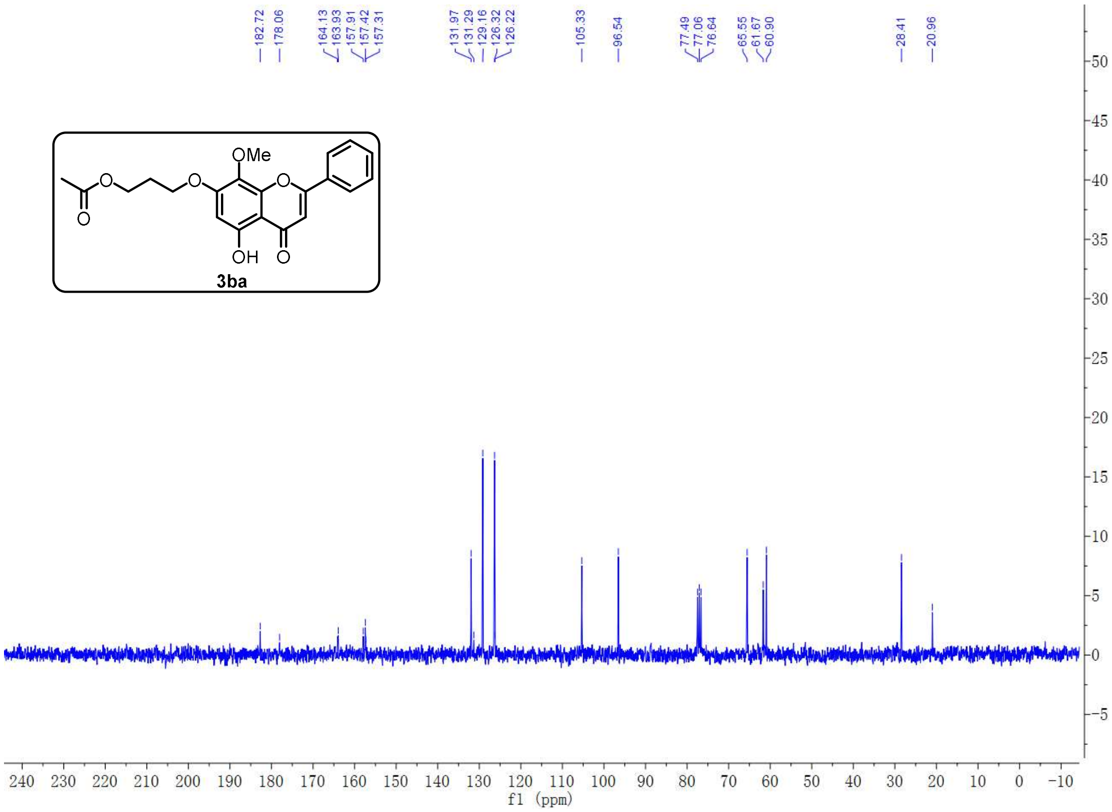
182.72
178.06
164.13
163.93
157.91
157.42
157.31
131.97
131.29
129.16
126.32
126.22
105.33
96.54
77.49
77.06
76.64
65.55
61.67
60.90
28.41
20.96
OMe
OH
3ba
f1 (ppm)
